# Comprehensive DIA‐MS Proteomics of Root Basal Nodes Elucidates Mechanisms of Salt Tolerance in Rice

**DOI:** 10.1002/pmic.70138

**Published:** 2026-04-29

**Authors:** Cheol Woo Min, Choonseok Lee, Ravi Gupta, Gi Hyun Lee, Ji Soo Kim, Na Won Park, Joong Hyoun Chin, Ki‐Hong Jung, Sun Tae Kim

**Affiliations:** ^1^ Department of Plant Bioscience Life and Industry Convergence Research Institute Pusan National University Miryang Republic of Korea; ^2^ Graduate School of Green‐Bio Science and Crop Biotech Institute Kyung Hee University Yongin Republic of Korea; ^3^ Plant Stress Physiology and Proteomics Laboratory College of General Education Kookmin University Seoul Republic of Korea; ^4^ Department of Integrative Biological Sciences and Industry College of Life Sciences Sejong University Seoul Republic of Korea

**Keywords:** DIA‐MS, *Oryza sativa*, proteomics, root basal node, salinity stress

## Abstract

Soil salinity severely affects rice growth, yield, and quality, posing a global food security challenge. Rice is particularly vulnerable to high salinity, which restricts growth and tolerance to other stresses. To address this, breeding efforts have been made in the past, leading to the generation of multi‐stress‐tolerant rice lines. A key achievement is the introgression of Submergence 1 (Sub1), Anaerobic Germination 1 (AG1), and Pi9 QTLs into the Indonesian variety Ciherang, generating the CSA‐Pi9 line with improved tolerance to submergence, salinity, and blast disease. In this study, we applied data‐independent acquisition mass spectrometry (DIA‐MS)–based proteomics of root basal nodes to explore salt tolerance in three rice cultivars, including CSA‐Pi9, CSA (Ciherang+Sub1+AG1), and Dongjin (DJ). We identified 3016 differentially modulated proteins under salinity. Functional annotation revealed that CSA‐Pi9 activates coordinated protective processes contributing to superior performance, such as sodium exclusion with potassium retention, membrane remodeling, and cuticle reinforcement via lipid and sterol metabolism, and sustained energy production with balanced sugar, nitrogen, and water metabolism, compared to the sensitive cultivar DJ under salt stress. These proteome‐wide insights highlight complex regulatory networks underlying salinity tolerance in rice and provide potential molecular targets for breeding strategies to enhance crop resilience under adverse environments.

1

The plant root system is in direct contact with the soil and is the first to experience high salinity. It is also essential for enabling the acquisition of water and nutrients from the soil, which is fundamental for sustaining plant growth [1]. Plant roots adapt to changing soil conditions through multiple mechanisms, including activity of membrane transporters that regulate ion and water uptake from the soil, their radial transport within the root, and subsequent loading into the xylem [2]. Particularly, the root basal node, located at the junction between root and shoot, serves as an important physiological interface that coordinates ion transport, hormonal signaling, and vascular connectivity between belowground and aboveground tissues. The root basal node is therefore known to play critical roles in diverse stress responses, including ion transport, hormonal signaling, and structural adaptation. A recent study has reported that morphological and genetic alterations in the rice root basal node under abiotic stress are closely associated with reductions in biomass and yield [3]; however, such changes have not yet been investigated during salt stress, especially at the proteome level.

To evaluate the impact of salinity stress on rice, a Korean domestic rice cultivar, Dongjin (DJ), which is susceptible to high salinity, was used, and its responses were compared with two tolerant cultivars, CSA and CSA‐Pi9. The CSA cultivar was developed from the Indonesian rice cultivar Ciherang through the introgression of the submergence tolerance gene (Sub1) and the anaerobic germination tolerance gene (AG1) into a single genetic background, whereas the CSA‐Pi9 line was generated by incorporating the broad‐spectrum blast resistance gene Pi9 into the CSA background [4]. Using these three cultivars, comparative proteomic analyses were performed under control and salt stress conditions to investigate cultivar‐specific proteome responses associated with salinity tolerance. Fifteen‐day‐old rice seedlings were transplanted into soil treated with 1 g of Na (2.542 g NaCl) per 1 kg of soil. Phenotypic observations were conducted on three individual plants per cultivar after 32 days of transplanting into soil. Notably, tiller numbers were significantly changed in CSA and CSA‐Pi9, while no significant change was observed in DJ (Figure 1A). CSA‐Pi9 plants maintained higher tiller numbers under salinity compared to DJ, suggesting a stronger ability to sustain vegetative growth. In addition, dry weight measurements further highlighted the contrasting stress tolerance among cultivars (Figure 1B). Besides, DJ and CSA plants exhibited a marked reduction in biomass under salt stress, with CSA showing the most significant decline (*p*‐value < 0.0001). In contrast, CSA‐Pi9 plants sustained biomass accumulation with no significant reduction compared to their control counterparts, reinforcing their superior tolerance to salinity stress.

**FIGURE 1:**
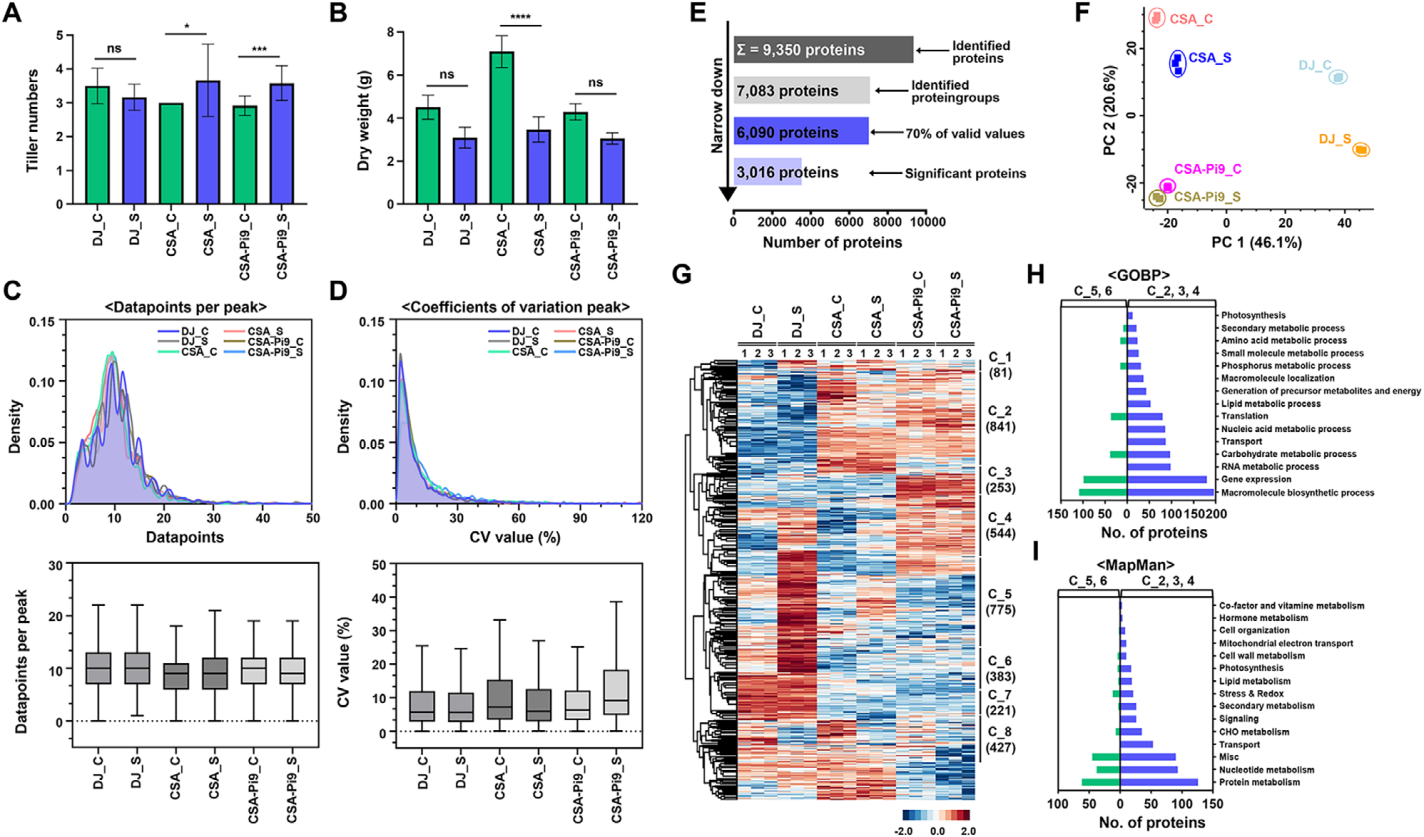
Evaluation of physiological changes and global proteomic profiling of rice root node samples under salinity stress using a DIA‐MS strategy. Quantitative assessment of plant physiological parameters under salt treatment. (A) Tiller number and (D) dry weight of each plant were measured for control (DJ_C, CSA_C, CSA‐Pi9_C) and salt‐treated (DJ_S, CSA_S, CSA‐Pi9_S) samples (*n* = 3 for three independent biological replicates). Statistical analysis was performed using student's *t*‐test*: 0.01<*p*<0.05, **: 0.001<*p*<0.01, ***: 0.0001<*p*<0.001, ****: *p*<0.0001, ns: not significant. (C) Distribution of datapoints per chromatographic peak across experimental groups. (D) Density plot of coefficient of variation (CV) values across all detected features, indicating high reproducibility among biological replicates. (E) Bar graph summarizing the total number of identified proteins and those showing statistical significance. (F) PCA plot showing distinct separation of sample groups along principal components 1 and 2. (G) Label‐free quantitative proteomic profiling of rice root nodes using the DIA‐MS approach. HCA of DMPs across six experimental groups, classified into eight distinct clusters based on abundance patterns. (H) GOBP enrichment analysis of DMPs in clusters (C)_2, 3, and 4 predominantly increased in CSA‐Pi9_S (salinity‐tolerant cultivar) and clusters C_5 and 6 showing increased abundance in DJ_S (salinity‐sensitive cultivar). (I) Functional classification of the same protein clusters using MapMan analysis.

To provide proteome‐wide insights into the adaptive strategies for salt tolerance mechanisms in rice, we conducted data‐independent acquisition‐mass spectrometry (DIA‐MS)‐based comparative analysis using three rice cultivars (DJ, CSA, and CSA‐Pi9) under control and salt stress conditions, utilizing a total of 25 isolation windows by overlapping each 1 mass to charge ratio (*m*/*z*) window with a full scan mass range from 400 to 1200 *m*/*z* in MS2 analysis (Figures S1A) [5]. The predicted number of average data points per chromatographic peaks was 9.5, and CV values of triplicates in each sample set were ∼9% among sample sets (Figure 1C,D). DIA‐MS analysis led to the identification of 70,066 peptides matched with 7083 protein groups, of which 6090 proteins showed two or more valid values in at least one sample group of DJ, CSA, and CSA‐Pi9 (Figure 1E). Principal component analysis (PCA) showed a distinct separation of six sample sets in PC1 and PC2, which account for 46.1% and 20.6% of the variations, respectively (Figure 1F). In addition, the multi‐scatter plots revealed Pearson correlation ranging from 0.910 to 0.991 across the six sample sets, indicating a strong correlation and high reproducibility of the obtained DIA‐MS data (Figure S1B). Application of multiple sample test controlled by Benjamini–Hochberg FDR threshold 0.05 with ≥1.5‐fold change as per previous report [6] revealed 3016 differentially modulated proteins (DMPs) among six sample sets (Figure 1G and Supporting Table in Mendeley data; DOI: 10.17632/c5k7jbg5pm.2). Hierarchical clustering analysis (HCA) classified these DMPs into eight clusters exhibiting differential‐regulation among six samples labeled as DJ‐control (DJ_C), DJ‐salt treated (DJ_S), CSA‐control (CSA_C), CSA‐salt treated (CSA_S), CSA‐Pi9‐control (CSA‐Pi9_C), and CSA‐Pi9‐salt treated (CSA‐Pi9_S) (Figure 1G). Of these DMPs, 1,094 and 554 proteins were localized in clusters_2, 3, and 4 (C_2, 3, and 4), which mainly showed increased abundance in CSA and/or CSA‐Pi9 sample sets, while proteins included in C_4 showed higher abundance in the DJ_S sample as well (Figure 1G). In contrast, proteins involved in C_5 (775) and C_6 (383) showed increased abundance only in the DJ sample sets (Figure 1G). Functional annotation by gene ontology for biological process (GOBP) enrichment analysis revealed that all the processes majorly enriched in C_2, 3, and 4 were increased in CSA‐Pi9 sample sets as compared to DJ sets (Figure 1H and Table S1). Especially, macromolecule biosynthetic process (GO:0009059, 196 and 109 proteins), gene expression (GO:0010467, 180 and 99 proteins), RNA metabolic process (GO:0016070, 98 proteins), carbohydrate metabolic process (GO:0005975, 97 and 39 proteins), transport (GO:0006810, 87 proteins), nucleic acid metabolic process (GO:0090304. 86 proteins), translation (GO:0006412, 80 and 37 proteins), lipid metabolic process (GO:0006629, 53 proteins), generation of precursor metabolites and energy (GO:0006091, 43 proteins), macromolecule localization (GO:0033036, 37 proteins), phosphorus metabolic process (GO:0006793, 31 and 16 proteins), small molecule metabolic process (GO:0044281, 26 proteins), amino acid metabolic process (GO:0006520, 23 and 16 proteins), secondary metabolic process (GO:0019748, 21 and 9 proteins), and photosynthesis (GO:0015979, 12 proteins) were identified in CSA and CSA‐Pi9 sample sets (Figure 1H and Table S1). In addition, functional annotation of significant DMPs with increased abundance in CSA‐Pi9 sample sets during salt stress by MapMan analysis showed that those proteins were mainly associated with amino acid metabolism (126 and 62 proteins), DNA/RNA/nucleotide metabolism (93 and 38 proteins), transport (53 proteins), carbon metabolism (35 and 7 proteins), and signaling (26 and 1 proteins), among others (Figure 1I and Table S2). In particular, 79, 18, 40, 17, 41, 25, 75, and 4 DMPs included in major metabolic pathways such as carbon metabolism, photosynthesis, nucleotide metabolism, amino acid metabolism, lipid metabolism, secondary metabolism, transport, and hormone metabolism were specifically increased in the CSA‐Pi9_S sample (Figure 2A and Table S3). Especially, out of these, 40 proteins corresponding to sodium exclusion, membrane remodeling and stabilization, cell wall reinforcement, cuticle and wax reinforcement, energy production, metabolic homeostasis, and nitrogen regulation showed increased abundance in CSA‐Pi9_S than DJ_S (Figure 2B and Table S4). Notably, we identified 94 proteins that showed salt‐induced increased abundance specifically in CSA‐Pi9 (Figure 2C). Specifically, those 94 proteins were mainly related to carbohydrate and starch metabolism, lipid and cuticular wax biosynthesis, ion and membrane transport, redox homeostasis, stress signaling, and secondary metabolite biosynthesis (Table S5). Among these, 20 proteins exhibited more than 1.5‐fold increase not only in the comparison between CSA‐Pi9_S and CSA‐Pi9_C, but also in the comparison between CSA‐Pi9_S and DJ_S (Figure 2D and Table S6).

**FIGURE 2:**
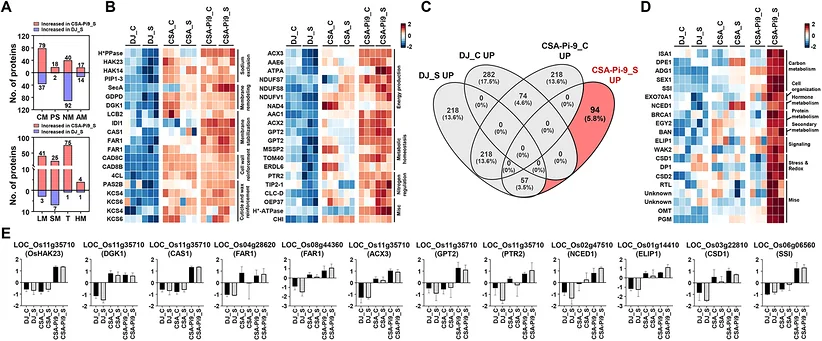
Functional characterization of selected proteins associated with various cellular metabolism. (A) Bar charts indicating the number of DMPs associated with specific metabolic categories, including carbon metabolism (CM), photosynthesis (PS), nucleotide metabolism (NM), amino acid metabolism (AM), lipid metabolism (LM), secondary metabolism (SM), transport (T), and hormone metabolism (HM), revealing distinct pathway enrichment patterns between CSA‐Pi9_S and DJ_S. (B) Heatmap depicting the abundance profiles of key proteins involved in sodium exclusion, membrane stabilization, cell wall reinforcement, energy metabolism, metabolite homeostasis, and nitrogen assimilation. (C) The Venn diagram indicates the commonly and specifically increased proteins in each cultivar under salt stress. (D) Heatmap showing the abundance profiles of key proteins specifically showed increased abundance in CSA‐Pi9_S as compared to CSA‐Pi9_C and DJ_S, respectively. (E) Transcript abundance of selected candidate genes corresponding to DMPs identified in DIA‐based proteomics analysis. These genes are implicated in key biological processes, including ion homeostasis, lipid metabolism, cell wall modification, and stress response regulation. Error bars represent standard deviation across biological replicates.

## 2 Enhanced Ion Homeostasis Contributes to Salinity Tolerance in CSA‐Pi9

The salt‐induced proteins included a potassium transporter 14 (OsHAK14) and an OsHAK23, exhibiting 1.65‐ and 2.14‐fold increase, respectively, in CSA‐Pi9_S than DJ_S (Figure 2B and Table S4), suggesting enhanced potassium homeostasis in the salt‐tolerant cultivar under salinity stress. Notably, RNA‐seq analysis revealed that the mRNA expression of OsHAK23 was also increased 1.74‐fold in CSA‐Pi9_S than in DJ_S (Figure 2E and Table S7). Under salt stress conditions, accumulation of Na^+^ is accompanied by the loss of K^+^ in plant cells [7]. In rice, 27 OsHAK family proteins have been identified, which regulate salt‐tolerance by mediating cellular K^+^/Na^+^ homeostasis [8]. Specifically, OsHAK1, OsHAK16, and OsHAK21 contribute to K^+^ uptake under salinity stress and help regulate Na^+^ exclusion, which improves salt tolerance [9, 10, 11]. Overexpression of *OsHAK5* results in significant improvement of K^+^ accumulation in rice shoot and biomass under salinity stress [12]. Although it remains unknown whether OsHAK14 and 23 also participate in K^+^/Na^+^ homeostasis or Na^+^ exclusion during high salinity conditions, the increased abundance of these two proteins observed in our study may suggest their potential involvement in salinity stress tolerance through similar mechanisms.

## 3 Improved Salinity Tolerance in CSA‐Pi9 is Associated With Reprogramming of Lipid‐ Associated Metabolism and Membrane Stability

During salinity stress, rapid reprogramming of lipid‐associated metabolism and proteins targeting/trafficking may contribute to reducing ion leakage and enhancing the physical stability of cellular membranes [13, 14]. Our results revealed increased protein and mRNA levels of a diacylglycerol kinase (DGK) by 3.07‐ and 4.02‐fold, respectively, in CSA‐Pi9_S as compared to DJ_S (Figure 2B,E; Table S4 and S7). The phospholipase‐C (PLC)/DGK pathway is pivotal for stress signaling and allows conversion of diacylglycerol to phosphatidic acid, the crucial second messengers in plant stress signaling [15]. We also identified long‐chain base biosynthesis protein 2d (Serine palmitoyltransferase, LCB2), exhibiting a 1.55‐fold increase, and a cycloartenol synthase 1 (CAS1) showing an 8.71‐fold increase in CSA‐Pi9_S as compared with DJ_S (Figure 2B and Table S4). Serine palmitoyltransferase (SPT), composed of the LCB1 and LCB2 subunits, catalyzes the first committed step in sphingolipid biosynthesis from serine with palmitoyl‐CoA to LCBs [16]. Sphingolipids stabilize various organelle membranes, and SPT indirectly provides essential components, including sphingolipids, ceramides, and very long‐chain fatty acids (VLCFAs), required for maintaining membrane integrity [17, 18]. Although the precise role of LCB2 under salt stress has not yet been elucidated, findings from Gan et al. 2009 suggested that LCB2 suppresses Bcl‐2‐associated X (Bax)‐mediated cell death [19]. In addition, several studies linking sphingolipid metabolism to membrane stabilization suggest that LCB2 may directly/indirectly contribute to enhanced tolerance against salt stress. Another sterol metabolism‐associated protein, CAS1, exhibited 8.71‐ and 9.11‐fold increase in protein and transcript levels, respectively, in CSA‐Pi9_S than DJ_S (Figure 2B,E; Table S4 and S7). CAS1 participates in sterol biosynthesis, along with exhibiting substantial impact on cell viability and physiological specifications [20]. Plant sterols not only contribute to maintaining membrane integrity, fluidity, and permeability but also regulate various cellular pathways such as endocytosis, cell signaling, plant‐pathogen interaction, and hormone metabolism [21, 22, 23]. Babiychuk et al. 2008 demonstrated that the *cas1‐1* mutant affects chlorophyll differentiation and pigment accumulation in Arabidopsis, along with a substantial impact on sterol biosynthesis, cell viability, and physiological parameters [20]. Although there is no direct evidence of CAS1's involvement in improving salt tolerance, various studies demonstrated that sterols themselves actively contribute to salt tolerance in wheat [24], tobacco [25], and *Brassica* species [26]. CAS1 catalyzes the first committed step of sterol biosynthesis, and its high abundance may influence sterol pools in plants. Thus, it may contribute to the maintenance of membrane integrity during salinity stress conditions.

## 4 Reinforcement of Cuticle and Wax Biosynthesis Enhances Structural Protection Under Salinity Stress

Salt stress also induces drastic alterations in plant surface barriers such as cuticular wax and suberin layers, which prevent the Na^+^‐induced dehydration and osmotic stress on plant cells [27]. Notably, VLCFAs are incorporated into cuticular waxes and the suberin barrier [28]. Very‐long‐chain (3R)‐3‐hydroxyacyl‐CoA dehydratase PASTICCINO 2 (PAS2) and 3‐ketoacyl‐CoA synthase (KCS) enzymes are major components of multiple fatty acid elongation complexes, which sequentially catalyze to produce the broad chain length range of the VLCFAs found in plant suberin and cuticular waxes [28]. In our study, we observed increased abundance of VLCFA biosynthesis‐related proteins, a PAS2B, with a 1.85‐fold increase in CSA‐Pi9_S as compared to DJ_S (Figure 2B and Table S4). In Arabidopsis, a mutation in *PAS2* results in the development of an abnormal phenotype due to lower VLCFA content and reduced density of wax layers than WT [29]. Moreover, we identified two isozymes of fatty acyl‐CoA reductase 1 (OsFAR1) with increased abundance (1.52‐ and 1.79‐fold increase, respectively) in CSA‐Pi9_S than DJ_S (Figure 2B and Table S4). RNA‐seq analysis also revealed their 2.68‐ and 1.89‐fold higher expression in CSA‐Pi9_S than DJ_S (Figure 2E and Table S7). FAR serves as a pivotal enzyme responsible for catalyzing the VLCFAs into primary alcohols and playing a crucial role in mediating responses to abiotic stresses, development, hormones, and growth [30]. Previous studies have demonstrated that distinct FAR isoforms display specific preferences for acyl chain length, leading to the synthesis of fatty alcohols of varying chain lengths [31]. Therefore, the accumulation of these membrane remodeling and structural integrity‐associated proteins suggests that CSA‐Pi9 cultivar may improve salinity tolerance by reinforcing lipid and sterol metabolism and enhancing wax and cuticular biosynthesis. Such multifaceted reinforcement of membrane and cell wall architecture provides a crucial barrier against ion toxicity and water loss, thereby maintaining cellular homeostasis under salt stress conditions.

In addition to these proteins, two isoforms of acyl‐coenzyme A oxidase (ACX2 and 3) also exhibited increased abundance in the CSA‐Pi9 sample under salt stress (Figure 2B and Table S4). Hydrolyzation of the triacylglycerols into fatty acids by peroxisomal *β*‐oxidation to generate ATP under energy‐deficient conditions during abiotic stresses is one of the important features in plants [32, 33]. We observed a 1.98‐ and 1.75‐fold increase in protein and mRNA levels of ACX3, respectively, in CSA‐Pi9_S than DJ_S (Figures 2B,E; Tables S4 and S7). The ACX enzyme is involved in the *β*‐oxidation of fatty acids and plays an important role in growth and stress response in plants [34]. A previous study demonstrated that overexpression of *ACX2* and *ACX3* improves biotic and abiotic stress tolerance in Arabidopsis [34, 35].

## 5 Enhanced Energy Metabolism Supports Salinity Stress Adaptation in CSA‐Pi9

Soil salinity also perturbs photosynthesis, carbon, and nitrogen homeostasis and ion balance. Therefore, proteins related to the sugar flux maintenance are required for the cellular energy and osmotic balance [36]. Here, we identified five proteins with increased abundance in CSA‐Pi9_S related to maintenance of the metabolic homeostasis, including two isoforms of glucose‐6‐phosphate/phosphate‐translocator 2 (GPT2), monosaccharide‐sensing protein 2 (MSSP2; also known as tonoplast monosaccharide transporter 2; TMT2), POT domain containing peptide transporter 2 (PTR2) family, and tonoplast intrinsic protein 2‐1 (TIP2‐1) (Figure 2B and Table S4). RNA‐seq analysis also revealed that *GPT2* and *PTR2* exhibit 1.68‐ and 1.83‐fold increase in CSA‐Pi9_S than DJ_S (Figure 2E and Table S7). The GPT2 mediates the exchange of glucose‐6‐phosphate to inorganic phosphate across the plastid membrane [37]. Knockout mutant of *gpt2* not only displays low seed yield during natural light environment, but also lower starch level and elevated levels of many sugar intermediates under high light conditions than WT [37, 38]. Notably, GPT2‐mediated import of cytosolic glucose‐6‐phosphate into chloroplasts can provide an alternative carbon supply that fuels starch biosynthesis or the glucose‐6‐phosphate shunt for providing metabolic flexibility. Therefore, this process mainly contributes to the redistribution of energy/metabolites, along with sustaining the ATP production and photosynthetic efficiency. Although changes of GPT2 enzyme under salinity stress in rice have not yet been reported, our study suggests its potential role in improving salt stress mitigation by maintaining photosynthesis rate and energy production.

Sugar proton‐coupled antiporter, MSSP2 (or TMT2), contributes to sugar import in the vacuole during stress response [39]. A previous study demonstrated that *TMT2* is specifically expressed in root and stem tissues, and the addition of 200 mM NaCl in growth media induces its expression in Arabidopsis. These results indicate that TMT2 is involved in vacuolar monosaccharide transport in the root and might play an important role during stress response. PTR2, a symporter and cotransporter of protons (H^+^), also plays key roles in the transport of a wide range of nitrogen‐containing substrates like nitrate (NO_3_^−^), amino acids, and di‐/tri‐peptides to plant roots [40]. Ouyang et al. 2010 reported up‐regulation of *OsPTR2* and other *OsPTR* isoforms in rice under drought and salinity stress conditions, highlighting their crucial roles in stress mitigation [41].

TIPs mainly consist of five subgroups, which are predominantly localized in the tonoplast [42]. TIP2‐1 is the most abundant aquaporin in the tonoplast, and we observed its >2.61‐fold increased abundance in CSA‐Pi9_S than DJ_S (Figure 2B and Table S4). Structural and functional analyses of TIP2‐1 revealed that it not only facilitates the ammonia (NH_3_) and ammonium (NH_4_^+^) transport across tonoplast, but also functions as water‐conducting membrane pores in plants [43, 44, 45]. Taken together, the increased abundance of TIP2‐1 along with GPT2, PTR2, and TMT2 in the salt‐tolerant rice cultivar highlights a coordinated network for maintaining metabolic homeostasis in rice, which enables the plant to avoid salt stress‐induced metabolic imbalance.

## 6 Maintenance of Metabolic Homeostasis and Solute Transport Contributes to Stress Resilience

Along with these proteins, we also observed salt stress‐induced abundance of several proteins potentially associated with the activation of starch–sugar conversion for energy supply, hormonal regulation, and maintenance of intracellular redox homeostasis exclusively in the CSA‐Pi9 cultivar (Figure 2D and Table S4). In particular, starch excess 1 (SEX1) and starch synthase I (SSI), known to have opposing functions for starch–sugar interconversion, were simultaneously increased in CSA‐Pi9_S as compared with both CSA‐Pi9_C and DJ_S, respectively (Figure 2D and Table S4). Among these, SEX1, also known as α‐glucan/water dikinase, has been reported to phosphorylate starch granules and facilitate starch degradation [46]. Previous report demonstrated that re‐mobilization of starch reserves provides soluble sugars that act as osmolytes and energy substrates for plant growth under osmotic stress conditions [47]. In contrast, SSI plays a distinct role in starch production, which fundamentally determines the fine structure of endosperm amylopectin by elongation of α‐1,4 glucan chain in rice [48]. Under salt stress, the accumulation of starch granules in chloroplasts is thought to serve as osmolytes, thereby facilitating water uptake and maintaining the structural integrity of the chloroplasts, as well as providing an essential energy supply for the cells [49]. Although no direct evidence has yet linked the accumulation of both SEX1 and SSI to salt tolerance in rice, their concurrent increased abundance indicates a coordinated strategy to alleviate osmotic stress and sufficient energy supply during salt stress in rice.

## 7 ABA Biosynthesis and ROS Scavenging Systems are Activated in Response to Salinity Stress

Abscisic acid (ABA) is a key hormone known to play a central role in enhancing plant tolerance to various environmental stresses by activating antioxidant defense systems, regulating ion efflux, and promoting stomatal closure [50, 51]. Exogenous ABA application improves rice survival and growth under salt and alkaline stresses through up‐regulation of stress‐related genes [51]. In this study, we identified 1.57‐ and 2.08‐fold increased abundance of a nine‐cis‐epoxycarotenoid dioxygenase 1 (NCED1) protein in CSA‐Pi9_S than in CSA‐Pi9_C and DJ_S, respectively (Figure 2D and Table S6). Moreover, the mRNA level of NCED1 also exhibited a 2.76‐fold increase in CSA‐Pi9_S than DJ_S (Figure 2E and Table S7). NCED catalyzes the first committed step in ABA biosynthesis and is known to be associated with responses to multiple abiotic stresses [52, 53]. Previous studies have revealed that NCED1 is positively correlated with salt, drought, and heat stress tolerance by regulation of the endogenous ABA contents in rice and Arabidopsis [54, 55].

An early light‐inducible protein 1 (ELIP1) exhibited a specific accumulation pattern in the CSA‐Pi9_S sample, showing a 2.72‐and 5.30‐fold increase compared with CSA‐Pi9_C and DJ_S, respectively (Figure 2D and Table S6). RNA‐seq analysis also showed its up‐regulation in CSA‐Pi9_S sample with 1.7‐ and 9.7‐fold increased than CAS‐Pi9_C and DJ_S samples, respectively (Figure 2E and Table S7). However, some discrepancies were observed between the expression profiles of the identified proteins and their corresponding transcripts (Figure 2E). Such differences may arise from multiple layers of regulation, including posttranscriptional and posttranslational mechanisms [56]. ELIP family proteins are localized in the thylakoid membranes and are well known for their photoprotective roles under light stress by transiently binding to free chlorophyll [57]. However, Zeng et al. 2002 demonstrated that the two *ELIP* genes (*ELIPa* and *ELIPb*) are responsive to a range of stress factors, including slow desiccation, rapid desiccation and rehydration, salinity, ABA, and rehydration under high‐light conditions in *Tortula ruralis* [58]. However, their exact role in salt stress mitigation in rice is still elusive and needs to be investigated.

Accumulation of reactive oxygen species (ROS) is a common feature during stress; therefore, diverse antioxidant enzymes are often activated as a part of the adaptive stress response in plants [59]. Here, we identified two subunits of copper/zinc superoxide dismutase (Cu/ZnSOD, CSD1, and CSD2) with respectively 1.61‐ and 1.54‐fold increased abundance in CSA‐Pi9_S as compared to CSA‐Pi9_C (Figure 2D and Table S6). mRNA expression level of CSD1 was also increased 2.12‐fold increase in CSA‐Pi9_S as compared to DJ_S (Figure 2E and Table S7). Both cytosolic CDS1 and chloroplastic CDS2 are shown to be associated with scavenging of salt stress‐induced ROS for prevention of oxidative damage and photoinhibition in rice and Arabidopsis [60, 61].

Taken together, our results suggest that the root basal node, which is positioned at the anatomical interface between the root and shoot, serves as a critical hub for ion transport and signal exchange. We identified key salt stress‐responsive proteins in this tissue, especially those associated with ion transport (OsHAK14 and OsHAK23), membrane remodeling (DGK and CAS1), and energy homeostasis (GPT2 and TIP2‐1), that possibly affect the node‐specific adaptations to the unique physiological demands of this tissue. In addition, the increased abundance of the ABA biosynthetic enzyme NCED1 and ROS‐scavenging enzymes (CSD1 and CSD2) may contribute to systemic stress signaling beyond the root basal node. Collectively, our results provide insights into the integrated regulation of the ion balance, membrane and cell wall stabilization, energy metabolism, and metabolic homeostasis during salt‐tolerance. Future efforts should be on the validation of these key identified proteins to establish their roles in salinity tolerance in rice and other crop plants.

## Funding

This work was supported by the National Research Foundation of Korea (NRF), funded by the Ministry of Education, Science, and Technology (grant numbers RS‐2022NR072241, RS‐2023‐00217064, RS‐2024‐00344229, and RS‐2025‐02214096 provided to CWM and STK, respectively).

## Conflicts of Interest

The authors declare no conflicts of interest.

## Supporting information




**Supporting File 1**: Supporting information.docx.

**Supporting File 2**: pmic70138‐sup‐0002‐TableS1.docx.

**Supporting File 3**: pmic70138‐sup‐0003‐TableS2.docx.

**Supporting File 4**: pmic70138‐sup‐0004‐TableS3.docx.

**Supporting File 5**: pmic70138‐sup‐0005‐TableS4.docx.

**Supporting File 6**: pmic70138‐sup‐0006‐TableS5.docx.

**Supporting File 7**: pmic70138‐sup‐0007‐TableS6.docx.

**Supporting File 8**: pmic70138‐sup‐0008‐TableS7.docx.

## Data Availability

The mass spectrometry proteomics data have been deposited to the ProteomeXchange Consortium via the PRIDE partner repository with the dataset identifier PXD068619 [62]. Supplementary tables have been deposited to the Mendeley Data with the data identification number DOI: 10.17632/c5k7jbg5pm. 2 (Title: The supplementary data for Comprehensive DIA‐MS proteomics of root basal nodes elucidates mechanisms of salt tolerance in rice, direct URL link: https://data.mendeley.com/datasets/c5k7jbg5pm/2).
