# Supplementary material for: Comprehensive DIA‐MS Proteomics of Root Basal Nodes Elucidates Mechanisms of Salt Tolerance in Rice
Source: Proteomics. 2026 Apr 29;26(9):140–7. doi: 10.1002/pmic.70138 (PMC13519373; doi:10.1002/pmic.70138)
Supplement: Supplementary file 1 [file PMIC-26--s001.docx]

**Table S5.** Comprehensive list of 94 proteins with increased abundance in the CSA-Pi9_S sample compared to CSA-Pi9_C and DJ_S

| **Locus IDs** | **Uniprot IDs** | **Protein name** | **Protein Symbols** | **DJ_C** | **DJ_S** | **CSA_C** | **CSA_S** | **CSA-Pi9_C** | **CSA-Pi9_S** | **FC (CSA-Pi9_S vs CSA-Pi9_C)** | **FC (CSA-Pi9_S vs DJ_S)** |
| --- | --- | --- | --- | --- | --- | --- | --- | --- | --- | --- | --- |
| LOC_Os02g47510.1 | Q6YVJ0 | NCED4 (NINE-CIS-EPOXYCAROTENOID DIOXYGENASE 4) | NCED4 | -0.1068 | -1.312 | -0.704 | 1.3159 | -0.3631 | 1.1699 | 1.573144894 | 2.08239 |
| LOC_Os09g26820.1 | Q69P90 | ATEXO70D1 (exocyst subunit EXO70 family protein D1); protein binding | EXO70D1 | -0.2571 | -1.0405 | -0.1921 | -0.4018 | 0.3175 | 1.574 | 1.558735791 | 2.5182485 |
| LOC_Os08g14109.1 | Q0J722 | dienelactone hydrolase family protein | - | -0.5731 | -0.1739 | 0.1556 | -1.0167 | 0.0457 | 1.5625 | 1.773559924 | 1.9269578 |
| LOC_Os07g09140.1 | Q6Z4D6 | transferase, transferring glycosyl groups | - | -0.2008 | -0.2125 | 0.4535 | -0.9454 | -0.5012 | 1.4064 | 2.501368047 | 2.177309 |
| LOC_Os01g14410.1 | Q9XHY5 | ELIP1 (EARLY LIGHT-INDUCABLE PROTEIN); chlorophyll binding | ELIP1 | -0.7172 | -0.7621 | 0.738 | -0.5566 | 0.0432 | 1.2549 | 2.721934239 | 5.2953161 |
| LOC_Os06g05630.1 | B9FRH9 | GDSL-motif lipase/hydrolase family protein | - | -0.6376 | -0.1367 | 0.8317 | -1.1695 | 0.0273 | 1.0848 | 1.567206479 | 1.680259 |
| LOC_Os01g51754.4 | Q5JLT1 | AMY3 (ALPHA-AMYLASE-LIKE 3); alpha-amylase | AMY3 | -0.8641 | -0.6529 | 0.8339 | -0.2877 | -0.4276 | 1.3984 | 1.797206139 | 1.9320146 |
| LOC_Os01g03630.1 | Q5ZE00 | LPR1 (Low Phosphate Root1); copper ion binding / oxidoreductase, acting on diphenols and related substances as donors, oxygen as acceptor | LPR1 | 0.0092 | -0.6762 | 1.0462 | -0.1311 | -1.4244 | 1.1763 | 3.080866473 | 2.2289346 |
| LOC_Os11g45295.1 | A0A0P0Y585 | retrotransposon protein, putative, unclassified, expressed | - | -1.2709 | -0.8875 | 0.945 | 0.7712 | -0.0606 | 0.5029 | 1.706460736 | 3.7387198 |
| LOC_Os04g48140.1 | Q7XRW8 | dehydration-responsive protein-related | - | -0.6408 | -0.6119 | 1.6834 | -0.0057 | -0.6924 | 0.2675 | 1.602161629 | 1.5400623 |
| LOC_Os11g13694.1 | Q0ITL9 | Uncharacterized protein | - | -1.0526 | -0.8993 | 1.2262 | 0.3846 | -0.1076 | 0.4487 | 1.659881641 | 3.413897 |
| LOC_Os01g12940.1 | Q0JPC4 | catalytic | - | -1.3094 | -1.1791 | 1.2693 | 0.176 | 0.2839 | 0.7594 | 1.912682934 | 14.063646 |
| LOC_Os01g15780.1 | Q5NBB8 | glycosyl transferase family 1 protein | - | -1.2514 | -0.8727 | 1.3054 | 0.9101 | -0.4343 | 0.3429 | 1.572926182 | 2.0308472 |
| LOC_Os08g36650.1 | Q7F805 | Unknown | - | -1.419 | -1.2778 | 0.5239 | 0.5472 | 0.6538 | 0.972 | 1.616441731 | 29.839481 |
| LOC_Os07g35940.1 | Q6Z5B2 | BAM5 (BETA-AMYLASE 5); beta-amylase | BETA-AMY | -1.3899 | -1.2576 | 0.4695 | 1.0664 | 0.3942 | 0.7173 | 1.622835231 | 19.291932 |
| LOC_Os07g13530.1 | Q7XHP7 | Ras-related GTP-binding family protein | - | -1.1814 | -0.9579 | 0.146 | 1.3869 | 0.1292 | 0.4771 | 1.537115515 | 5.8913705 |
| LOC_Os12g24390.1 | Q2QSE5 | zinc finger (C3HC4-type RING finger | - | -0.3694 | -0.7593 | 0.762 | 1.0246 | -0.9953 | 0.3374 | 3.050930748 | 2.5039775 |
| LOC_Os09g30010.1 | Q651Y5 | FLA1 (FASCICLIN-LIKE ARAB INOGALACTAN 1) | FLA1 | -0.958 | -0.2906 | 1.363 | 0.9148 | -1.009 | -0.0202 | 1.764221902 | 1.1679846 |
| LOC_Os06g45360.1 | Q655Z0 | ATAPP1; N-1-naphthylphthalamic acid binding / aminopeptidase | APP1 | 0.1944 | -0.7242 | 0.7713 | -0.7993 | -0.7424 | 1.3002 | 1.833296173 | 1.8234475 |
| LOC_Os01g38480.1 | Q656U1 | Uncharacterized protein | - | 0.4871 | 0.3557 | 0.1278 | -0.4376 | -1.4376 | 0.9046 | 21.73486687 | 2.0577083 |
| LOC_Os09g17650.1 | A0A0P0XKF8 | Unknown | - | -0.5417 | -0.2306 | 1.2233 | 0.2941 | -1.4565 | 0.7114 | 4.995809438 | 2.01172 |
| LOC_Os07g44410.1 | Q8GVH2 | tolB protein-related | - | -0.1149 | 1.1342 | 0.1569 | -0.1478 | -1.8755 | 0.8472 | 1.767167108 | -1.061861 |
| LOC_Os02g32970.1 | Q6ESF3 | hydrolase, alpha/beta fold family protein | - | -0.2102 | 0.649 | 0.266 | 0.4216 | -1.789 | 0.6626 | 1.683704975 | 1.0028847 |
| LOC_Os03g22810.1 | Q10LG5 | CSD1 (COPPER/ZINC SUPEROXIDE DISMUTASE 1); superoxide dismutase | CSD1 | -1.0969 | 0.4526 | 0.3227 | 0.171 | -1.2639 | 1.4146 | 1.610191564 | 1.1865975 |
| LOC_Os11g42500.1 | Q2R0I1 | disease resistance-responsive family protein | - | -0.0151 | 0.6623 | -0.9618 | -0.7181 | -0.7322 | 1.7649 | 2.752924108 | 1.5638852 |
| LOC_Os03g51930.1 | B9FBB9 | Uncharacterized protein | - | -0.6851 | 0.3506 | -0.6796 | -0.5561 | -0.3894 | 1.9597 | 1.621055436 | 1.3922156 |
| LOC_Os10g06140.1 | Q7XGR2 | WAK2; ATP binding / calcium ion binding / protein kinase/ protein serine/threonine kinase | WAK2 | -0.3387 | -0.2873 | -1.1265 | -0.4316 | 0.5346 | 1.6495 | 2.549310663 | 5.0826304 |
| LOC_Os11g20160.1 | Q53LW0 | O-methyltransferase family 2 protein | - | -0.5001 | -1.0428 | -0.3213 | -0.3687 | 0.2457 | 1.9873 | 1.682033722 | 2.4712168 |
| LOC_Os08g40930.1 | D0TZF0 | ISA1 (ISOAMYLASE 1); alpha-amylase/ isoamylase | ISA1 | 0.0596 | -0.6433 | -0.7127 | -0.048 | -0.6116 | 1.956 | 1.809752044 | 1.8230731 |
| LOC_Os10g11140.1 | Q33AE4 | PGM (PHOSPHOGLUCOMUTASE); phosphoglucomutase | PGM | -1.0973 | -1.0587 | -0.0615 | 0.3069 | 0.1193 | 1.7913 | 1.69823957 | 2.4662166 |
| LOC_Os08g44770.1 | P93407 | CSD2 (COPPER/ZINC SUPEROXIDE DISMUTASE 2); superoxide dismutase | CSD2 | -1.3032 | -0.1127 | 0.136 | 0.2554 | -0.6736 | 1.6981 | 1.536606538 | 1.3881673 |
| LOC_Os04g55480.1 | Q7XTV9 | zinc finger (ubiquitin-hydrolase | - | -0.7798 | -0.2593 | 0.0932 | -0.0629 | -0.797 | 1.8057 | 3.124855096 | 2.4694852 |
| LOC_Os04g53780.1 | B9FCP2 | BAN (BANYULS); oxidoreductase | BAN | -1.036 | -0.9224 | 0.3907 | -0.2532 | 0.2569 | 1.564 | 1.541177102 | 2.2768612 |
| LOC_Os01g04900.1 | Q0JQS5 | EGY2; metalloendopeptidase | EGY2 | -1.2114 | -0.9372 | 0.6861 | 0.2194 | -0.281 | 1.5241 | 5.496466135 | 10.212371 |
| LOC_Os07g43390.1 | F2ZBU6 | DPE1 (DISPROPORTIONATING ENZYME); 4-alpha-glucanotransferase/ catalytic/ cation binding | DPE1 | -1.0209 | -0.1226 | -0.9543 | 1.0293 | -0.4765 | 1.5449 | 1.553794374 | 1.4384057 |
| LOC_Os09g12660.1 | Q69T99 | ADG1 (ADP GLUCOSE PYROPHOSPHORYLASE 1); glucose-1-phosphate adenylyltransferase | ADG1 | -1.0879 | -1.2439 | -0.1282 | 0.1716 | 0.7825 | 1.5059 | 1.53315618 | 5.075705 |
| LOC_Os06g30310.1 | B9FTF7 | SEX1 (STARCH EXCESS 1); alpha-glucan, water dikinase | SEX1 | -0.7532 | -1.053 | -0.4373 | 0.223 | 0.1001 | 1.9205 | 1.564127119 | 2.0765268 |
| LOC_Os06g06560.1 | B7F2B2 | SSI1 (SUPPRESSOR OF SALICYLIC ACID INSENSITIVITY 1); starch synthase/ transferase, transferring glycosyl groups | SSI1 | -0.7287 | -0.7996 | -0.6838 | -0.1963 | 0.4946 | 1.9138 | 1.52600294 | 2.2435652 |
| LOC_Os05g32710.1 | Q6AU80 | isoamylase, putative / starch debranching enzyme, putative | ISA2 | -0.8621 | -0.7466 | -0.4007 | -0.3748 | 0.4273 | 1.9569 | 1.510628303 | 2.0732538 |
| LOC_Os03g55090.1 | Q9AUV8 | glucan phosphorylase, putative | - | -0.9319 | -0.8867 | -0.7134 | 0.3101 | 0.4361 | 1.7859 | 1.523234099 | 2.3007526 |
| LOC_Os01g63270.1 | Q8LQ33 | PHS2 (ALPHA-GLUCAN PHOSPHORYLASE 2); phosphorylase/ transferase, transferring glycosyl groups | PHS2 | -0.7672 | -1.3349 | -0.2989 | 0.0215 | 0.7651 | 1.6145 | 1.521438153 | 4.2938091 |
| LOC_Os06g49090.1 | Q0D9Q1 | zinc finger protein-related | - | 0.4476 | 0.3176 | -1.7659 | -0.3513 | 0.4363 | 0.9157 | 1.517922849 | 1.6831934 |
| LOC_Os03g50300.1 | Q10E21 | AtCASP (Arabidopsis thaliana CCAAT-displacement protein alternatively spliced product) | CASP | 0.21 | 0.6787 | -1.4571 | 0.8032 | -0.6927 | 0.4578 | 1.678271237 | -1.104551 |
| LOC_Os05g05710.1 | Q65XE2 | FASS (FASS 1); protein phosphatase type 2B regulator | FASS | -0.2635 | 0.785 | -1.6723 | 0.396 | -0.2595 | 1.0143 | 1.613571014 | 1.0899574 |
| LOC_Os02g01890.1 | Q6ETT1 | CYP89A6; electron carrier/ heme binding / iron ion binding / monooxygenase/ oxygen binding | CYP89A6 | -0.4742 | 0.7834 | -1.0993 | -0.1489 | -0.2325 | 1.1714 | 1.522682075 | 1.1232337 |
| LOC_Os06g16640.1 | Q5VPU9 | Uncharacterized protein | - | -0.0456 | 0.5759 | -1.058 | 0.8685 | -1.3333 | 0.9925 | 1.816622715 | 1.1128401 |
| LOC_Os10g17454.1 | Q109W0 | GR-RBP3 (glycine-rich RNA-binding protein 3); ATP binding / RNA binding | GR-RBP3 | -0.2214 | 1.6357 | -0.1902 | -0.4786 | -1.3938 | 0.6484 | 1.503656373 | -1.21797 |
| LOC_Os08g07400.1 | Q6YYL1 | CUL3B (CULLIN 3B); protein binding / ubiquitin-protein ligase | CUL3B | -0.0959 | 1.1407 | -0.0365 | -0.5705 | -1.2543 | 0.8165 | 1.653252573 | -1.081896 |
| LOC_Os09g15480.1 | Q6K2D5 | Uncharacterized protein | - | 0.8952 | 0.8634 | 0.2543 | -0.5675 | -1.3581 | -0.0872 | 2.051030095 | -1.711394 |
| LOC_Os04g59494.4 | B9FDR3 | TRN1 (TRANSPORTIN 1); protein transporter | TRN1 | 0.7199 | 1.1321 | 0.5223 | -1.002 | -1.0973 | -0.2749 | 1.709948414 | -2.503979 |
| LOC_Os02g58790.1 | Q6EP22 | SULA; binding / catalytic/ coenzyme binding | GC1 | 1.0819 | 0.4287 | -0.1124 | -0.1792 | -1.5265 | 0.3075 | 1.691184513 | -1.035321 |
| LOC_Os01g40550.1 | Q8S1F2 | Unknown | - | 0.1918 | 0.7097 | -0.0156 | 0.4955 | -1.5893 | 0.2079 | 1.601040447 | -1.140416 |
| LOC_Os01g09320.1 | P43279 | ATNADP-ME4 (NADP-malic enzyme 4); malate dehydrogenase (oxaloacetate-decarboxylating | NADP-ME4 | 0.6741 | 0.7258 | 0.2948 | 0.337 | -1.6449 | -0.3869 | 2.591130875 | -2.321164 |
| LOC_Os11g03390.1 | Q2RB31 | forkhead-associated domain-containing protein / FHA domain-containing protein | - | 0.3279 | 0.988 | -0.2943 | 0.26 | -1.9467 | 0.6651 | 1.817043863 | -1.076626 |
| LOC_Os08g25430.1 | Q0J6A8 | CRK10 (CYSTEINE-RICH RLK10) | CRK10 | 0.163 | 0.8997 | 0.2354 | -0.3114 | -1.5524 | 0.5657 | 2.64370148 | -1.16568 |
| LOC_Os05g15770.1 | Q5WMX0 | acidic endochitinase (CHIB1) | - | 0.2893 | 1.4086 | -0.1703 | 0.2002 | -1.7833 | 0.0555 | 1.534233542 | -1.370213 |
| LOC_Os01g72290.1 | Q942A8 | GLP10 (GERMIN-LIKE PROTEIN 10); manganese ion binding / nutrient reservoir | GLP10 | 0.4533 | 1.33 | -0.4292 | -0.1701 | -1.5804 | 0.3964 | 1.745782724 | -1.301002 |
| LOC_Os12g12590.1 | Q2QVJ5 | NADP-dependent oxidoreductase, putative | - | 1.1818 | 0.5299 | 0.1736 | -0.3425 | -1.7999 | 0.2572 | 1.730420611 | -1.075384 |
| LOC_Os07g09150.1 | Q6Z4D5 | Uncharacterized protein | - | 0.6526 | 1.3845 | 0.1779 | -0.198 | -1.5481 | -0.469 | 1.73845316 | -2.585428 |
| LOC_Os04g49530.1 | Q7XP63 | Uncharacterized protein | - | 0.8285 | 1.0807 | 0.3636 | -0.0941 | -1.6291 | -0.5495 | 1.573026002 | -1.981912 |
| LOC_Os03g57160.1 | Q10CF1 | zinc ion binding | - | 0.9687 | 1.0155 | 0.0697 | -0.352 | -1.4532 | -0.2487 | 4.096614371 | -4.393368 |
| LOC_Os02g02400.1 | B7EAG0 | CAT1 (CATALASE 1); catalase | C1 | 0.674 | 0.778 | -0.2628 | 0.6093 | -2.0176 | 0.2191 | 2.015228919 | -1.191324 |
| LOC_Os01g43890.1 | Q9FP87 | scpl50 (serine carboxypeptidase-like 50); serine-type carboxypeptidase | scpl50 | 0.5163 | 0.1287 | 1.1757 | -0.1366 | -1.4728 | -0.2113 | 2.190965373 | -1.23537 |
| LOC_Os01g56470.1 | Q5ZD06 | Uncharacterized protein | - | 0.7888 | 0.607 | 0.3987 | -0.0021 | -1.5704 | -0.222 | 2.246291751 | -1.644744 |
| LOC_Os08g19420.1 | Q6Z9P5 | O-methyltransferase family 2 protein | - | 0.8009 | 0.9348 | 0.4039 | 0.2114 | -1.533 | -0.818 | 2.300916831 | -7.711597 |
| LOC_Os05g09440.1 | Q6T5D1 | ATNADP-ME3 (NADP-malic enzyme 3); malate dehydrogenase (oxaloacetate-decarboxylating | NADP-ME3 | 1.1133 | 0.9408 | 0.0757 | 0.0064 | -1.4368 | -0.6994 | 1.689174543 | -3.20981 |
| LOC_Os04g40420.1 | Q0JCC3 | ATSWI3A (SWITCH/SUCROSE NONFERMENTING 3A); DNA binding | SWI3A | 0.3458 | 1.334 | -0.1851 | -0.7374 | -1.0785 | 0.3212 | 1.514806473 | -1.350506 |
| LOC_Os08g43020.1 | B9FYD6 | HCT (HYDROXYCINNAMOYL-COA SHIKIMATE/QUINATE HYDROXYCINNAMOYL TRANSFERASE) | HCT | 0.6346 | 1.1768 | -1.2357 | 0.2095 | -1.3028 | 0.5177 | 1.808961853 | -1.239389 |
| LOC_Os02g51470.1 | Q6Z8K7 | ATPD (ATP SYNTHASE DELTA-SUBUNIT GENE); hydrogen ion transporting ATP synthase, rotational mechanism / proton-transporting ATPase, rotational mechanism | PD | 0.5565 | 1.1772 | -1.1182 | 0.5695 | -1.4432 | 0.2582 | 1.634352583 | -1.303858 |
| LOC_Os08g28790.1 | Q6ZDJ2 | Uncharacterized protein | - | 0.9613 | 1.5751 | -0.6527 | -0.6076 | -1.0986 | -0.1775 | 1.561551616 | -2.33509 |
| LOC_Os02g57120.1 | Q6K6B1 | CLASP (CLIP-ASSOCIATED PROTEIN); binding | CLASP | 0.7622 | 1.1417 | -1.0605 | -0.1889 | -0.9406 | 0.2861 | 1.764289155 | -1.485826 |
| LOC_Os04g09720.1 | Q7XSG1 | scpl10 (serine carboxypeptidase-like 10); serine-type carboxypeptidase | scpl10 | 1.1325 | 1.1544 | 0.2087 | -0.3463 | -1.3465 | -0.8028 | 1.528240628 | -4.602198 |
| LOC_Os08g39300.1 | Q6ZFI6 | AGT (ALANINE:GLYOXYLATE AMINOTRANSFERASE); alanine-glyoxylate transaminase/ serine-glyoxylate transaminase/ serine-pyruvate transaminase | AGT | 1.4376 | 1.0247 | -0.5828 | 0.0163 | -1.3852 | -0.5106 | 1.755027401 | -2.684521 |
| LOC_Os07g11110.1 | Q8GTK8 | CSP41A (CHLOROPLAST STEM-LOOP BINDING PROTEIN OF 41 KDA); mRNA binding / poly(U | CSP41A | 1.3367 | 0.6408 | -0.3314 | -0.5524 | -1.5639 | 0.4701 | 1.658460589 | -1.043369 |
| LOC_Os06g39690.1 | Q69VD1 | SOUL heme-binding family protein | - | 1.5797 | 0.7512 | -0.4986 | -0.6801 | -1.1825 | 0.0302 | 1.70197294 | -1.371828 |
| LOC_Os06g01210.1 | Q0DFC9 | DRT112; copper ion binding / electron carrier | DRT112 | 0.7947 | 1.1035 | -0.7108 | 0.2307 | -1.4691 | 0.051 | 1.535891615 | -1.345974 |
| LOC_Os03g32270.1 | Q10J20 | hydrolase, alpha/beta fold family protein | - | 1.2069 | 1.0344 | -0.3409 | -0.1403 | -1.3898 | -0.3702 | 1.502510098 | -1.752166 |
| LOC_Os10g09850.1 | Q33AI7 | calcium-binding EF hand family protein | - | 1.0194 | 1.2789 | -0.1844 | -0.9216 | -0.845 | -0.3474 | 1.561726419 | -4.292665 |
| LOC_Os06g39780.1 | Q69X58 | CYP76C4; electron carrier/ heme binding / iron ion binding / monooxygenase/ oxygen binding | CYP76C4 | 1.425 | 1.1803 | -0.5203 | -0.7672 | -0.871 | -0.4468 | 1.77682725 | -9.071744 |
| LOC_Os04g42690.1 | Q7F8X6 | UDP-glucoronosyl/UDP-glucosyl transferase family protein | - | 1.4402 | 1.1731 | -0.6183 | -0.7264 | -0.9691 | -0.2996 | 2.383828817 | -6.75899 |
| LOC_Os03g44880.1 | Q7XZY1 | GLP5 (GERMIN-LIKE PROTEIN 5); manganese ion binding / nutrient reservoir | GLP5 | 1.1969 | 1.4541 | -0.8052 | -0.795 | -0.7928 | -0.258 | 1.841603361 | -7.062707 |
| LOC_Os12g07290.1 | Q2QX39 | FUT13 (FUCOSYLTRANSFERASE 13); fucosyltransferase/ transferase, transferring glycosyl groups | FUT13 | 0.8199 | 0.7977 | -0.2806 | -1.3971 | -0.5383 | 0.5985 | 3.753253596 | -1.260843 |
| LOC_Os03g19580.1 | Q10MH1 | Uncharacterized protein | - | 0.8709 | 1.078 | 0.1278 | -1.4172 | -0.7737 | 0.1142 | 1.511598236 | -1.565996 |
| LOC_Os06g51084.1 | Q0D9D0 | SBE2.2 (starch branching enzyme 2.2); 1,4-alpha-glucan branching enzyme | SBE2.2 | 1.0341 | 0.721 | -0.9112 | -1.0471 | -0.9143 | 1.1175 | 1.8409461 | 1.1264735 |
| LOC_Os03g62370.1 | Q6AVR6 | Uncharacterized protein | - | 1.0335 | 1.2685 | -1.0966 | -1.2421 | -0.3206 | 0.3574 | 2.315359666 | -3.08952 |
| LOC_Os02g48210.1 | Q0DY65 | lectin protein kinase family protein | - | 1.13 | 1.2981 | -0.3769 | -0.8421 | -1.0456 | -0.1636 | 2.184751785 | -3.651869 |
| LOC_Os01g71540.2 | B7FA83 | Unknown | - | 1.0861 | 1.2635 | -0.5017 | -0.9309 | -0.7449 | -0.1721 | 2.2055426 | -7.259332 |
| LOC_Os01g10140.2 | A0A0P0UZ91 | RNA-dependent RNA polymerase family protein | - | 0.9016 | 0.9787 | -0.494 | -0.8023 | -1.0715 | 0.4875 | 1.797061998 | -1.20283 |
| LOC_Os12g38770.1 | Q2QN72 | calcineurin-like phosphoesterase family protein | - | 0.0949 | -0.2708 | 1.4278 | -0.8963 | -0.5802 | 0.2245 | 2.191672261 | 1.6208043 |
| LOC_Os11g05090.1 | Q2RAK4 | TWD1 (TWISTED DWARF 1); FK506 binding / calmodulin binding / peptidyl-prolyl cis-trans isomerase | TWD1 | -0.0729 | 0.1794 | 0.8888 | 1.0294 | -1.3863 | -0.6385 | 1.646183806 | -1.724991 |
| LOC_Os05g08770.1 | Q5KQK4 | leucine-rich repeat family protein / extensin family protein | - | 0.4728 | 1.1546 | 0.4171 | 0.411 | -1.7025 | -0.753 | 1.558701102 | -2.439354 |
| LOC_Os02g35080.1 | Q6ZI82 | T-complex protein 11 | - | 0.8165 | 0.838 | 0.392 | 0.5096 | -1.5676 | -0.9885 | 1.623721567 | -4.612489 |
| LOC_Os05g38310.1 | B9FKK7 | UBQ5 (ubiquitin 5); protein binding / structural constituent of ribosome | UBQ5 | -0.0981 | 0.0547 | 1.3653 | -0.114 | -1.2495 | 0.0417 | 1.898146968 | -1.0065 |
| LOC_Os01g03090.1 | Q5ZEM9 | AR401 | AR401 | 0.365 | 0.0924 | 0.8821 | 0.5991 | -1.7462 | -0.1923 | 2.099377874 | -1.145572 |
