# Supplementary material for: Comprehensive DIA‐MS Proteomics of Root Basal Nodes Elucidates Mechanisms of Salt Tolerance in Rice
Source: Proteomics. 2026 Apr 29;26(9):140–7. doi: 10.1002/pmic.70138 (PMC13519373; doi:10.1002/pmic.70138)
Supplement: Supplementary file 2 [file PMIC-26--s002.docx]

**Table S6.** List of 20 proteins showing more than 1.5-fold increased abundance in CSA-Pi9_S compared to both CSA-Pi9_C and DJ_S samples

| **Cat** | **Locus IDs** | **Uniprot IDs** | **Protein name** | **Protein Symbols** | **DJ_C** | **DJ_S** | **CSA_C** | **CSA_S** | **CSA-Pi9_C** | **CSA-Pi9_S** | **FC (CSA-Pi9_S vs CSA-Pi9_C)** | **FC (CSA-Pi9_S vs DJ_S)** |
| --- | --- | --- | --- | --- | --- | --- | --- | --- | --- | --- | --- | --- |
| Carbon metabolism | LOC_Os08g40930.1 | D0TZF0 | ISA1 (ISOAMYLASE 1); alpha-amylase/ isoamylase | ISA1 | 0.06 | -0.64 | -0.71 | -0.05 | -0.61 | 1.96 | 1.809752 | 1.823073 |
| Carbon metabolism | LOC_Os07g43390.1 | F2ZBU6 | DPE1 (DISPROPORTIONATING ENZYME); 4-alpha-glucanotransferase/ catalytic/ cation binding | DPE1 | -1.02 | -0.12 | -0.95 | 1.03 | -0.48 | 1.54 | 1.553794 | 1.438406 |
| Carbon metabolism | LOC_Os09g12660.1 | Q69T99 | ADG1 (ADP GLUCOSE PYROPHOSPHORYLASE 1); glucose-1-phosphate adenylyltransferase | ADG1 | -1.09 | -1.24 | -0.13 | 0.17 | 0.78 | 1.51 | 1.533156 | 5.075705 |
| Carbon metabolism | LOC_Os06g30310.1 | B9FTF7 | SEX1 (STARCH EXCESS 1); alpha-glucan, water dikinase | SEX1 | -0.75 | -1.05 | -0.44 | 0.22 | 0.10 | 1.92 | 1.564127 | 2.076527 |
| Carbon metabolism | LOC_Os06g06560.1 | B7F2B2 | SSS1 (Soluble starch synthase 1), chloroplastic/amyloplastic | SSI1 | -0.73 | -0.80 | -0.68 | -0.20 | 0.49 | 1.91 | 1.526003 | 2.243565 |
| Cell organization&division&transport | LOC_Os09g26820.1 | Q69P90 | exocyst complex component EXO70A1 | EXO70A1 | -0.26 | -1.04 | -0.19 | -0.40 | 0.32 | 1.57 | 1.558736 | 2.518249 |
| Hormone metabolism | LOC_Os02g47510.1 | Q6YVJ0 | NCED1 (NINE-CIS-EPOXYCAROTENOID DIOXYGENASE 1) | OsNCED1 | -0.11 | -1.31 | -0.70 | 1.32 | -0.36 | 1.17 | 1.573145 | 2.08239 |
| Protein metabolism | LOC_Os04g55480.1 | Q7XTV9 | BRCA1-associated protein | BRCA1 | -0.78 | -0.26 | 0.09 | -0.06 | -0.80 | 1.81 | 3.124855 | 2.469485 |
| Protein metabolism | LOC_Os01g04900.1 | Q0JQS5 | EGY2; metalloendopeptidase | EGY2 | -1.21 | -0.94 | 0.69 | 0.22 | -0.28 | 1.52 | 5.496466 | 10.21237 |
| Secondary metabolism | LOC_Os04g53780.1 | B9FCP2 | BAN (BANYULS); oxidoreductase | BAN | -1.04 | -0.92 | 0.39 | -0.25 | 0.26 | 1.56 | 1.541177 | 2.276861 |
| Signaling | LOC_Os01g14410.1 | Q9XHY5 | ELIP1 (EARLY LIGHT-INDUCABLE PROTEIN); chlorophyll binding | ELIP1 | -0.72 | -0.76 | 0.74 | -0.56 | 0.04 | 1.25 | 2.721934 | 5.295316 |
| Signaling | LOC_Os10g06140.1 | Q7XGR2 | WAK2; ATP binding / calcium ion binding / protein kinase/ protein serine/threonine kinase | WAK2 | -0.34 | -0.29 | -1.13 | -0.43 | 0.53 | 1.65 | 2.549311 | 5.08263 |
| Stress & Redox | LOC_Os03g22810.1 | Q10LG5 | CSD1 (COPPER/ZINC SUPEROXIDE DISMUTASE 1); superoxide dismutase | CSD1 | -1.10 | 0.45 | 0.32 | 0.17 | -1.26 | 1.41 | 1.610192 | 1.186597 |
| Stress & Redox | LOC_Os11g42500.1 | Q2R0I1 | Dirigent protein 1 | DP1 | -0.02 | 0.66 | -0.96 | -0.72 | -0.73 | 1.76 | 2.752924 | 1.563885 |
| Stress & Redox | LOC_Os08g44770.1 | P93407 | CSD2 (COPPER/ZINC SUPEROXIDE DISMUTASE 2); superoxide dismutase | CSD2 | -1.30 | -0.11 | 0.14 | 0.26 | -0.67 | 1.70 | 1.536607 | 1.388167 |
| Misc | LOC_Os08g14109.1 | Q0J722 | retrotransposon protein, putative, Ty1-copia subclass | RTL | -0.57 | -0.17 | 0.16 | -1.02 | 0.05 | 1.56 | 1.77356 | 1.926958 |
| Misc | LOC_Os07g09140.1 | Q6Z4D6 | transferase, transferring glycosyl groups | Unknown | -0.20 | -0.21 | 0.45 | -0.95 | -0.50 | 1.41 | 2.501368 | 2.177309 |
| Misc | LOC_Os03g51930.1 | B9FBB9 | unknown protein | Unknown | -0.69 | 0.35 | -0.68 | -0.56 | -0.39 | 1.96 | 1.621055 | 1.392216 |
| Misc | LOC_Os11g20160.1 | Q53LW0 | O-methyltransferase | OMT | -0.50 | -1.04 | -0.32 | -0.37 | 0.25 | 1.99 | 1.682034 | 2.471217 |
| Misc | LOC_Os10g11140.1 | Q33AE4 | PGM (PHOSPHOGLUCOMUTASE); phosphoglucomutase | PGM | -1.10 | -1.06 | -0.06 | 0.31 | 0.12 | 1.79 | 1.69824 | 2.466217 |
