# Supplementary material for: Comprehensive DIA‐MS Proteomics of Root Basal Nodes Elucidates Mechanisms of Salt Tolerance in Rice
Source: Proteomics. 2026 Apr 29;26(9):140–7. doi: 10.1002/pmic.70138 (PMC13519373; doi:10.1002/pmic.70138)
Supplement: Supplementary file 3 [file PMIC-26--s003.docx]

**Table S3.** List of selected increased abundance proteins in DJ_S and CSA-Pi9_S sample sets, respectively

| **Cat** | **Locus IDs** | **Uniprot IDs** | **Protein description** | **DJ_C** | **DJ_S** | **CSA_C** | **CSA_S** | **CSA-Pi9_C** | **CSA-Pi9_S** | **Cat** |
| --- | --- | --- | --- | --- | --- | --- | --- | --- | --- | --- |
| CM | LOC_Os05g32710.1 | Q6AU80 | Alpha amylase, catalytic domain containing protein | -0.86 | -0.75 | -0.40 | -0.37 | 0.43 | 1.96 | increased in CSA-Pi9_S |
| CM | LOC_Os08g40930.1 | D0TZF0 | Alpha amylase, catalytic domain containing protein | 0.06 | -0.64 | -0.71 | -0.05 | -0.61 | 1.96 | increased in CSA-Pi9_S |
| CM | LOC_Os09g29404.1 | B9G434 | glycogen operon glgX, putative | -0.35 | -1.00 | -0.75 | -0.22 | 0.44 | 1.87 | increased in CSA-Pi9_S |
| CM | LOC_Os02g32660.1 | Q6H6P8 | 1,4-alpha-glucan-branching enzyme, chloroplast precursor, putative | -0.25 | -1.38 | -0.20 | 0.06 | -0.04 | 1.80 | increased in CSA-Pi9_S |
| CM | LOC_Os01g63270.1 | Q8LQ33 | alpha-glucan phosphorylast isozyme, putative | -0.77 | -1.33 | -0.30 | 0.02 | 0.77 | 1.61 | increased in CSA-Pi9_S |
| CM | LOC_Os01g32364.1 | Q5QMT0 | Os1bglu1 - beta-mannosidase/glucosidase homologue | -0.62 | -1.16 | 0.04 | -0.65 | 0.77 | 1.61 | increased in CSA-Pi9_S |
| CM | LOC_Os08g25734.1 | P15280 | glucose-1-phosphate adenylyltransferase large subunit, chloroplast precursor, putative | -1.14 | -1.15 | -0.26 | 0.54 | 0.44 | 1.57 | increased in CSA-Pi9_S |
| CM | LOC_Os09g12660.1 | Q69T99 | glucose-1-phosphate adenylyltransferase large subunit, chloroplast precursor, putative | -1.09 | -1.24 | -0.13 | 0.17 | 0.78 | 1.51 | increased in CSA-Pi9_S |
| CM | LOC_Os09g23350.1 | Q6ERD9 | trehalose synthase, putative | -1.24 | -1.17 | -0.04 | 0.46 | 0.56 | 1.44 | increased in CSA-Pi9_S |
| CM | LOC_Os05g50380.1 | Q688T8 | glucose-1-phosphate adenylyltransferase large subunit, chloroplast precursor, putative | -0.98 | -1.38 | 0.12 | -0.03 | 0.86 | 1.42 | increased in CSA-Pi9_S |
| CM | LOC_Os01g51754.4 | Q5JLT1 | alpha-amylase precursor, putative | -0.86 | -0.65 | 0.83 | -0.29 | -0.43 | 1.40 | increased in CSA-Pi9_S |
| CM | LOC_Os06g12820.1 | A0A0P0WV15 | proteins of unknown function domain containing protein | -1.45 | -0.25 | 0.37 | -0.53 | 0.47 | 1.40 | increased in CSA-Pi9_S |
| CM | LOC_Os05g45720.1 | Q84VC7 | starch synthase, putative | -0.70 | -0.37 | -1.16 | 0.50 | 0.35 | 1.38 | increased in CSA-Pi9_S |
| CM | LOC_Os04g32550.1 | Q0JDK9 | defender against cell death 1, putative | -0.55 | -1.27 | -0.40 | -0.29 | 1.14 | 1.38 | increased in CSA-Pi9_S |
| CM | LOC_Os01g44220.1 | Q7G065 | glucose-1-phosphate adenylyltransferase large subunit, chloroplast precursor, putative | -1.20 | -1.31 | 0.25 | 0.28 | 0.78 | 1.21 | increased in CSA-Pi9_S |
| CM | LOC_Os01g70520.1 | Q5JK35 | Os1bglu5 - beta-glucosidase homologue, similar to G. max isohydroxyurate hydrolase | -1.61 | -0.96 | 0.34 | 0.50 | 0.54 | 1.20 | increased in CSA-Pi9_S |
| CM | LOC_Os01g33420.1 | Q5QLK3 | glycosyl hydrolase family 27, putative | -1.41 | -0.61 | 0.46 | 0.00 | 0.35 | 1.20 | increased in CSA-Pi9_S |
| CM | LOC_Os01g53680.1 | Q5JNC6 | 6-phosphofructokinase, putative | -0.89 | -1.62 | 0.53 | 0.52 | 0.26 | 1.18 | increased in CSA-Pi9_S |
| CM | LOC_Os04g39880.1 | Q7XKV4 | Os4bglu12 - beta-glucosidase, exo-beta-glucanase | -1.63 | -0.96 | 0.48 | 0.43 | 0.51 | 1.17 | increased in CSA-Pi9_S |
| CM | LOC_Os11g29390.1 | Q2R481 | bifunctional dihydrofolate reductase-thymidylate synthase, putative | -1.23 | -1.29 | 0.22 | 0.19 | 0.93 | 1.17 | increased in CSA-Pi9_S |
| CM | LOC_Os05g47980.1 | A0A067Y2M2 | ATP synthase, putative | -1.23 | -1.03 | 0.79 | 0.48 | -0.17 | 1.15 | increased in CSA-Pi9_S |
| CM | LOC_Os06g45370.1 | Q655Y9 | fructose-1,6-bisphosphatase, putative | -1.08 | -1.51 | 0.45 | 0.43 | 0.57 | 1.14 | increased in CSA-Pi9_S |
| CM | LOC_Os08g15030.1 | Q9LD61 | aspartate carbamoyltransferase, putative | -0.18 | -1.21 | -0.08 | -0.30 | 0.63 | 1.14 | increased in CSA-Pi9_S |
| CM | LOC_Os04g49960.1 | Q7XLZ9 | glycosyl transferase, group 1 domain containing protein | -0.79 | -1.38 | 0.15 | -0.16 | 1.06 | 1.12 | increased in CSA-Pi9_S |
| CM | LOC_Os02g57770.1 | Q6K9X1 | glycosyl hydrolases family 16, putative | -1.51 | -0.98 | 0.91 | -0.07 | 0.54 | 1.11 | increased in CSA-Pi9_S |
| CM | LOC_Os09g39570.1 | Q652P5 | beta-amylase, putative | -0.65 | -1.56 | 0.81 | -0.18 | 0.51 | 1.07 | increased in CSA-Pi9_S |
| CM | LOC_Os03g52460.1 | Q6AVT2 | glucose-1-phosphate adenylyltransferase large subunit, putative | -1.21 | -1.35 | 0.53 | 0.95 | 0.02 | 1.06 | increased in CSA-Pi9_S |
| CM | LOC_Os03g53860.1 | Q8W2Z2 | periplasmic beta-glucosidase precursor, putative | -0.82 | -1.63 | 0.91 | -0.11 | 0.72 | 0.94 | increased in CSA-Pi9_S |
| CM | LOC_Os11g12810.1 | Q53JI9 | sucrose-phosphate synthase, putative | -0.94 | -1.69 | 0.74 | 0.60 | 0.35 | 0.94 | increased in CSA-Pi9_S |
| CM | LOC_Os06g09450.1 | I3QD82 | sucrose synthase, putative | -0.50 | -1.93 | 0.32 | 0.47 | 0.72 | 0.91 | increased in CSA-Pi9_S |
| CM | LOC_Os01g72240.1 | Q9LDN2 | uridine 5-monophosphate synthase, putative | -1.29 | -1.38 | 0.67 | 0.66 | 0.46 | 0.87 | increased in CSA-Pi9_S |
| CM | LOC_Os06g25010.1 | B7F946 | Uncharacterized protein | -1.21 | -1.20 | 0.49 | 0.10 | 0.97 | 0.85 | increased in CSA-Pi9_S |
| CM | LOC_Os01g48200.1 | Q0JKI5 | callose synthase, putative | -0.71 | -0.97 | 0.53 | -0.79 | 1.11 | 0.82 | increased in CSA-Pi9_S |
| CM | LOC_Os09g21210.1 | Q6H5B5 | beta-glucan-binding 4, putative | -1.22 | -1.49 | 0.85 | 0.51 | 0.54 | 0.81 | increased in CSA-Pi9_S |
| CM | LOC_Os03g46910.1 | Q84R73 | pyruvate kinase, putative | -1.50 | -1.19 | 0.42 | 0.77 | 0.73 | 0.78 | increased in CSA-Pi9_S |
| CM | LOC_Os07g04240.1 | Q6ZDY8 | succinate dehydrogenase flavosubunit,mitochondrial precursor, putative | -0.74 | -1.62 | 1.21 | 0.36 | 0.01 | 0.77 | increased in CSA-Pi9_S |
| CM | LOC_Os06g41020.1 | Q69WY3 | DUF1680 domain containing protein, putative | -1.38 | -1.31 | 0.58 | 0.66 | 0.68 | 0.77 | increased in CSA-Pi9_S |
| CM | LOC_Os09g15820.1 | Q6K2P3 | aldose 1-epimerase, putative | 0.24 | -2.07 | 0.61 | 0.20 | 0.25 | 0.77 | increased in CSA-Pi9_S |
| CM | LOC_Os02g10070.1 | Q7F8R1 | citrate synthase, putative | -0.62 | -0.90 | -0.97 | 0.18 | 1.55 | 0.77 | increased in CSA-Pi9_S |
| CM | LOC_Os11g04740.1 | Q2RAP0 | FAD-binding and arabino-lactone oxidase domains containing protein, putative | -1.48 | -1.19 | 0.97 | 0.56 | 0.37 | 0.76 | increased in CSA-Pi9_S |
| CM | LOC_Os11g47330.1 | Q2QZ86 | ATP-grasp domain containing protein | -1.31 | -1.37 | 0.78 | 0.59 | 0.55 | 0.75 | increased in CSA-Pi9_S |
| CM | LOC_Os01g55780.1 | Q8LR39 | glycerophosphoryl diester phosphodiesterase family protein, putative | -0.77 | -1.79 | 0.82 | 0.48 | 0.54 | 0.73 | increased in CSA-Pi9_S |
| CM | LOC_Os07g35940.1 | Q6Z5B2 | beta-amylase, putative | -1.39 | -1.26 | 0.47 | 1.07 | 0.39 | 0.72 | increased in CSA-Pi9_S |
| CM | LOC_Os07g36630.1 | Q84S18 | CSLF8 - cellulose synthase-like family F; beta1,3;1,4 glucan synthase | -0.85 | -1.72 | 0.74 | 0.34 | 0.79 | 0.70 | increased in CSA-Pi9_S |
| CM | LOC_Os08g34900.1 | Q6ZLF6 | Uncharacterized protein | -0.57 | -1.59 | 0.82 | -0.07 | 0.74 | 0.67 | increased in CSA-Pi9_S |
| CM | LOC_Os11g03780.1 | Q2RAZ2 | alpha-N-arabinofuranosidase, putative | -1.12 | -1.58 | 0.85 | 0.50 | 0.69 | 0.65 | increased in CSA-Pi9_S |
| CM | LOC_Os04g46370.1 | Q0JB91 | Uncharacterized protein | -0.97 | -1.41 | 0.55 | 0.46 | 0.74 | 0.63 | increased in CSA-Pi9_S |
| CM | LOC_Os10g19880.1 | Q339Q0 | alpha-1,2-fucosidase, putative | -0.82 | -1.37 | 1.51 | 0.43 | -0.37 | 0.62 | increased in CSA-Pi9_S |
| CM | LOC_Os08g29710.1 | Q6ZA16 | galactosyltransferase family protein, putative | -0.99 | -1.16 | 0.84 | 0.04 | 0.66 | 0.61 | increased in CSA-Pi9_S |
| CM | LOC_Os12g04520.1 | Q2QXY1 | FAD-binding and arabino-lactone oxidase domains containing protein, putative | -1.50 | -1.17 | 0.77 | 0.84 | 0.49 | 0.57 | increased in CSA-Pi9_S |
| CM | LOC_Os04g39900.1 | Q7XKV2 | Os4bglu13 - beta-glucosidase homologue, similar to Os4Bglu12 exoglucanase/b-glucosidase | -1.24 | -1.42 | 0.92 | 0.64 | 0.54 | 0.57 | increased in CSA-Pi9_S |
| CM | LOC_Os01g05490.1 | P48494 | triosephosphate isomerase, cytosolic, putative | -1.27 | -1.33 | 1.05 | 0.86 | 0.12 | 0.57 | increased in CSA-Pi9_S |
| CM | LOC_Os10g17650.1 | Q339X2 | Uncharacterized protein | -0.82 | -1.56 | 1.24 | 0.26 | 0.32 | 0.56 | increased in CSA-Pi9_S |
| CM | LOC_Os11g03730.1 | Q2RAZ7 | alpha-N-arabinofuranosidase A, putative | -0.94 | -1.26 | 1.65 | 0.22 | -0.23 | 0.55 | increased in CSA-Pi9_S |
| CM | LOC_Os02g10190.1 | Q6H7P9 | DUF1680 domain containing protein, putative | -0.58 | -1.76 | 1.18 | 0.68 | -0.03 | 0.50 | increased in CSA-Pi9_S |
| CM | LOC_Os04g24430.1 | H6TFZ4 | sucrose synthase, putative | -0.99 | -1.69 | 0.76 | 0.73 | 0.70 | 0.49 | increased in CSA-Pi9_S |
| CM | LOC_Os06g47310.1 | Q653F8 | plant-specific domain TIGR01627 family protein | -1.24 | -0.75 | 1.65 | -0.04 | -0.07 | 0.45 | increased in CSA-Pi9_S |
| CM | LOC_Os05g33840.1 | O22567 | transketolase, putative | -1.33 | -1.33 | 0.79 | 0.92 | 0.49 | 0.45 | increased in CSA-Pi9_S |
| CM | LOC_Os01g70780.1 | Q8LR61 | WD40-like domain containing protein, putative | -0.27 | -1.77 | 1.22 | -0.05 | 0.41 | 0.45 | increased in CSA-Pi9_S |
| CM | LOC_Os04g40490.1 | Q7XUQ7 | glycosyl hydrolase family 5 protein, putative | -0.88 | -1.54 | 1.41 | 0.25 | 0.32 | 0.44 | increased in CSA-Pi9_S |
| CM | LOC_Os06g21570.1 | Q5Z9Z0 | Os6bglu24 - beta-glucosidase homologue, similar to G. max isohydroxyurate hydrolase | -0.82 | -1.50 | 1.16 | 0.07 | 0.67 | 0.41 | increased in CSA-Pi9_S |
| CM | LOC_Os11g37890.1 | Q2R1V8 | NAD dependent epimerase/dehydratase family protein, putative | -0.83 | -1.69 | 0.61 | 0.35 | 1.18 | 0.38 | increased in CSA-Pi9_S |
| CM | LOC_Os09g39440.1 | Q652Q8 | inosine-uridine preferring nucleoside hydrolase family protein, putative | -0.92 | -1.67 | 1.01 | 0.67 | 0.62 | 0.29 | increased in CSA-Pi9_S |
| CM | LOC_Os12g03470.1 | Q2QY88 | alpha-N-arabinofuranosidase A, putative | -1.18 | -1.08 | 1.56 | 0.50 | -0.07 | 0.27 | increased in CSA-Pi9_S |
| CM | LOC_Os04g48140.1 | Q7XRW8 | methyltransferase, putative | -0.64 | -0.61 | 1.68 | -0.01 | -0.69 | 0.27 | increased in CSA-Pi9_S |
| CM | LOC_Os03g13390.2 | Q10PE7 | oxidoreductase, aldo/keto reductase family protein, putative | -1.09 | -1.34 | 0.42 | 0.97 | 0.82 | 0.22 | increased in CSA-Pi9_S |
| CM | LOC_Os01g06660.1 | Q9LGL5 | thiamine pyrophosphate enzyme, C-terminal TPP binding domain containing protein | -1.16 | -1.35 | 1.22 | 0.68 | 0.40 | 0.20 | increased in CSA-Pi9_S |
| CM | LOC_Os03g20420.1 | Q10M79 | alpha-N-arabinofuranosidase A, putative | -0.42 | -1.60 | 1.68 | 0.06 | 0.09 | 0.19 | increased in CSA-Pi9_S |
| CM | LOC_Os02g58480.1 | Q6K973 | sucrose synthase, putative | -0.54 | -1.87 | 0.89 | 0.59 | 0.79 | 0.14 | increased in CSA-Pi9_S |
| CM | LOC_Os01g62420.1 | Q8LR75 | triosephosphate isomerase, cytosolic, putative | -1.04 | -1.35 | 1.15 | 1.08 | 0.05 | 0.11 | increased in CSA-Pi9_S |
| CM | LOC_Os10g21236.1 | Q8S6G0 | chloroplast ATP synthase a chain precursor, putative | -0.31 | -1.77 | 0.74 | 0.71 | 0.54 | 0.09 | increased in CSA-Pi9_S |
| CM | LOC_Os01g54300.1 | Q0JJD4 | OsMan02 - Endo-Beta-Mannanase | -0.97 | -1.12 | 0.44 | 0.71 | 0.86 | 0.08 | increased in CSA-Pi9_S |
| CM | LOC_Os01g57854.1 | Q5ZAW1 | pectinesterase, putative | -0.50 | -1.28 | 1.84 | 0.21 | -0.34 | 0.07 | increased in CSA-Pi9_S |
| CM | LOC_Os05g28180.1 | Q84NP7 | AMP deaminase, putative | -0.49 | -1.32 | -0.11 | 1.19 | 0.70 | 0.04 | increased in CSA-Pi9_S |
| CM | LOC_Os03g31170.1 | Q84MH2 | inosine-uridine preferring nucleoside hydrolase family protein, putative | -1.05 | -1.48 | 1.02 | 0.89 | 0.59 | 0.04 | increased in CSA-Pi9_S |
| CM | LOC_Os03g40670.1 | Q6AUZ6 | glycerophosphoryl diester phosphodiesterase family protein, putative | 0.47 | -1.76 | 1.42 | -0.42 | 0.33 | -0.05 | increased in CSA-Pi9_S |
| CM | LOC_Os05g47880.1 | Q6L4F9 | Uncharacterized protein | 0.66 | -1.32 | 1.27 | -0.53 | 0.01 | -0.09 | increased in CSA-Pi9_S |
| CM | LOC_Os05g36070.1 | B9FJ61 | signal peptide peptidase domain containing protein | -0.58 | -1.07 | 1.36 | -0.01 | 0.46 | -0.16 | increased in CSA-Pi9_S |
| CM | LOC_Os01g27880.1 | Q94E75 | sucrose-phosphatase, putative | 0.20 | -1.80 | 1.49 | 0.35 | -0.07 | -0.17 | increased in CSA-Pi9_S |
| PS | LOC_Os06g39708.1 | Q69VC8 | photosystem II P680 chlorophyll A apoprotein, putative | 0.30 | -1.28 | -0.49 | -0.47 | 0.09 | 1.84 | increased in CSA-Pi9_S |
| PS | LOC_Os05g49380.1 | Q6AUN5 | OsDegp9 - Putative Deg protease homologue | -1.25 | -0.96 | 0.13 | 0.15 | 0.34 | 1.60 | increased in CSA-Pi9_S |
| PS | LOC_Os02g24632.1 | Q6Z1U5 | photosystem II 44 kDa reaction center protein, putative | 0.95 | -1.37 | -0.50 | -0.57 | 0.00 | 1.50 | increased in CSA-Pi9_S |
| PS | LOC_Os04g16770.1 | Q0JEM7 | photosynthetic reaction center protein, putative | 0.29 | -1.19 | -0.85 | -0.34 | 0.60 | 1.49 | increased in CSA-Pi9_S |
| PS | LOC_Os07g37240.1 | Q6Z411 | chlorophyll A-B binding protein, putative | -0.24 | -1.35 | -0.86 | 0.27 | 0.69 | 1.48 | increased in CSA-Pi9_S |
| PS | LOC_Os08g33820.1 | Q6YWJ7 | chlorophyll A-B binding protein, putative | -0.28 | -1.53 | -0.21 | -0.31 | 0.88 | 1.45 | increased in CSA-Pi9_S |
| PS | LOC_Os07g32880.1 | Q84NW1 | ATP synthase gamma chain, putative | -1.25 | -1.16 | -0.27 | 0.67 | 0.69 | 1.32 | increased in CSA-Pi9_S |
| PS | LOC_Os06g45370.1 | Q655Y9 | fructose-1,6-bisphosphatase, putative | -1.08 | -1.51 | 0.45 | 0.43 | 0.57 | 1.14 | increased in CSA-Pi9_S |
| PS | LOC_Os02g24634.1 | Q6Z1U4 | photosystem II D2 protein, putative | 1.04 | -1.60 | -0.41 | -0.60 | 0.52 | 1.04 | increased in CSA-Pi9_S |
| PS | LOC_Os09g17740.1 | P12330 | chlorophyll A-B binding protein, putative | -0.09 | -1.12 | -0.91 | -0.40 | 1.48 | 1.04 | increased in CSA-Pi9_S |
| PS | LOC_Os11g13890.1 | Q53N83 | chlorophyll A-B binding protein, putative | -0.16 | -1.18 | -0.85 | -0.25 | 1.60 | 0.84 | increased in CSA-Pi9_S |
| PS | LOC_Os07g37550.1 | Q6ZF30 | chlorophyll A-B binding protein, putative | 0.23 | -1.01 | -1.07 | -0.50 | 1.52 | 0.83 | increased in CSA-Pi9_S |
| PS | LOC_Os01g25600.1 | Q5ZBW3 | Uncharacterized protein | -0.99 | -0.32 | -0.93 | 0.80 | 0.69 | 0.74 | increased in CSA-Pi9_S |
| PS | LOC_Os01g41710.1 | I3QD41 | chlorophyll A-B binding protein, putative | 0.08 | -0.88 | -1.05 | -0.53 | 1.71 | 0.67 | increased in CSA-Pi9_S |
| PS | LOC_Os01g05490.1 | P48494 | triosephosphate isomerase, cytosolic, putative | -1.27 | -1.33 | 1.05 | 0.86 | 0.12 | 0.57 | increased in CSA-Pi9_S |
| PS | LOC_Os01g52240.1 | Q8W0E6 | chlorophyll A-B binding protein, putative | 0.12 | -1.42 | -0.22 | -0.59 | 1.66 | 0.44 | increased in CSA-Pi9_S |
| PS | LOC_Os01g62420.1 | Q8LR75 | triosephosphate isomerase, cytosolic, putative | -1.04 | -1.35 | 1.15 | 1.08 | 0.05 | 0.11 | increased in CSA-Pi9_S |
| PS | LOC_Os10g21236.1 | Q8S6G0 | chloroplast ATP synthase a chain precursor, putative | -0.31 | -1.77 | 0.74 | 0.71 | 0.54 | 0.09 | increased in CSA-Pi9_S |
| NM | LOC_Os02g43600.1 | Q6H8H3 | 50S ribosomal L19, chloroplast precursor, putative | -1.18 | -0.29 | -0.63 | -0.48 | 0.94 | 1.63 | increased in CSA-Pi9_S |
| NM | LOC_Os02g01332.1 | Q0E4R7 | ribosomal L6, putative | -1.47 | -0.79 | 0.14 | -0.09 | 0.74 | 1.47 | increased in CSA-Pi9_S |
| NM | LOC_Os01g52490.1 | Q8W0C2 | Uncharacterized protein | 0.11 | -0.90 | -0.98 | 0.19 | 0.14 | 1.45 | increased in CSA-Pi9_S |
| NM | LOC_Os02g27760.1 | Q6K5R6 | 40S ribosomal S15a, putative | -1.49 | -0.47 | -0.24 | -0.10 | 0.93 | 1.37 | increased in CSA-Pi9_S |
| NM | LOC_Os01g47660.1 | Q0JKK9 | 60S ribosomal L18a, putative | -0.72 | -0.51 | -0.91 | -0.55 | 1.35 | 1.35 | increased in CSA-Pi9_S |
| NM | LOC_Os01g10820.1 | Q9LWN1 | ribosomal L5, putative | -0.71 | -0.66 | -0.52 | -0.82 | 1.40 | 1.31 | increased in CSA-Pi9_S |
| NM | LOC_Os03g18570.1 | Q10MS5 | 40S ribosomal S7, putative | -0.72 | -0.06 | -1.24 | -0.53 | 1.26 | 1.30 | increased in CSA-Pi9_S |
| NM | LOC_Os02g18380.1 | Q6K4T1 | 60S ribosomal L27-3, putative | -0.54 | -0.67 | -0.63 | -0.87 | 1.44 | 1.28 | increased in CSA-Pi9_S |
| NM | LOC_Os03g19960.1 | Q10MC7 | ATP-dependent RNA helicase, putative | -1.06 | -1.14 | -0.34 | 0.12 | 1.16 | 1.26 | increased in CSA-Pi9_S |
| NM | LOC_Os01g65220.1 | Q5N739 | XRN 5'-3' exonuclease N-terminus domain containing protein | -1.19 | -0.17 | -0.46 | -0.73 | 1.31 | 1.25 | increased in CSA-Pi9_S |
| NM | LOC_Os04g51630.1 | Q0JAC6 | 60S ribosomal L7, putative | -0.67 | -0.43 | -0.78 | -0.83 | 1.48 | 1.23 | increased in CSA-Pi9_S |
| NM | LOC_Os03g05980.1 | Q2R1J8 | 40S ribosomal S9-2, putative | -0.95 | -0.45 | -0.59 | -0.70 | 1.47 | 1.22 | increased in CSA-Pi9_S |
| NM | LOC_Os03g26229.1 | Q0DRI8 | 40S ribosomal S9-1, putative | -0.49 | 0.03 | -1.28 | -0.61 | 1.14 | 1.22 | increased in CSA-Pi9_S |
| NM | LOC_Os03g38000.1 | Q75G91 | 40S ribosomal S3-1, putative | -0.92 | -0.93 | -0.58 | -0.22 | 1.44 | 1.21 | increased in CSA-Pi9_S |
| NM | LOC_Os08g13690.1 | Q6YYR2 | 60S ribosomal L7, putative | -1.02 | -0.53 | -0.61 | -0.49 | 1.44 | 1.21 | increased in CSA-Pi9_S |
| NM | LOC_Os07g42950.1 | Q8LH97 | 40S ribosomal S6, putative | -0.81 | -0.59 | -0.98 | -0.07 | 1.24 | 1.20 | increased in CSA-Pi9_S |
| NM | LOC_Os05g27940.1 | Q8LJU5 | 40S ribosomal S7, putative | -0.95 | -0.13 | -1.20 | -0.22 | 1.33 | 1.18 | increased in CSA-Pi9_S |
| NM | LOC_Os09g24250.1 | Q69MX3 | ubiquitin carboxyl-terminal hydrolase domain containing protein | -0.97 | -0.51 | -1.04 | -0.01 | 1.37 | 1.16 | increased in CSA-Pi9_S |
| NM | LOC_Os02g40880.1 | Q6K1Q6 | ribosomal L14, putative | -0.77 | -0.59 | -0.60 | -0.67 | 1.48 | 1.14 | increased in CSA-Pi9_S |
| NM | LOC_Os02g51830.1 | Q6Z695 | Uncharacterized protein | 0.06 | 0.03 | -0.85 | -1.37 | 1.00 | 1.14 | increased in CSA-Pi9_S |
| NM | LOC_Os08g15030.1 | Q9LD61 | aspartate carbamoyltransferase, putative | -0.18 | -1.21 | -0.08 | -0.30 | 0.63 | 1.14 | increased in CSA-Pi9_S |
| NM | LOC_Os08g23710.1 | P0DKK7 | ribosomal L7Ae, putative | -1.01 | -0.59 | -0.84 | -0.12 | 1.42 | 1.13 | increased in CSA-Pi9_S |
| NM | LOC_Os10g41470.1 | Q7XC31 | 60S ribosomal L27-3, putative | -0.52 | -0.74 | -0.65 | -0.79 | 1.60 | 1.09 | increased in CSA-Pi9_S |
| NM | LOC_Os01g54870.1 | Q943F3 | 60S ribosomal L18a, putative | -0.40 | -0.87 | -0.62 | -0.73 | 1.59 | 1.03 | increased in CSA-Pi9_S |
| NM | LOC_Os11g09990.1 | Q53LJ0 | mTERF family protein | -0.32 | -0.77 | -1.01 | -0.01 | 1.08 | 1.02 | increased in CSA-Pi9_S |
| NM | LOC_Os08g40230.1 | Q6Z8N1 | Uncharacterized protein | -0.48 | -0.14 | -1.43 | -0.04 | 1.07 | 1.02 | increased in CSA-Pi9_S |
| NM | LOC_Os05g13950.1 | Q0DJU1 | eukaryotic translation initiation factor 3 subunit E-interacting protein, putative | -0.31 | -0.59 | -1.34 | -0.27 | 1.50 | 1.02 | increased in CSA-Pi9_S |
| NM | LOC_Os02g39720.2 | B9F110 | Uncharacterized protein | -0.59 | -0.23 | -0.48 | -0.86 | 1.20 | 0.96 | increased in CSA-Pi9_S |
| NM | LOC_Os02g03470.1 | B9F287 | tetratricopeptide repeat containing protein, putative | 0.23 | -1.20 | -0.84 | -0.59 | 1.45 | 0.96 | increased in CSA-Pi9_S |
| NM | LOC_Os04g39700.1 | Q7XR19 | 60S ribosomal L6, putative | -1.73 | -0.48 | -0.11 | 0.20 | 1.17 | 0.95 | increased in CSA-Pi9_S |
| NM | LOC_Os01g72240.1 | Q9LDN2 | uridine 5-monophosphate synthase, putative | -1.29 | -1.38 | 0.67 | 0.66 | 0.46 | 0.87 | increased in CSA-Pi9_S |
| NM | LOC_Os11g03290.1 | B9GBL8 | nucleoside-triphosphatase, putative | -1.33 | -1.25 | 1.20 | 0.35 | 0.22 | 0.81 | increased in CSA-Pi9_S |
| NM | LOC_Os05g42300.1 | Q60EI5 | Uncharacterized protein | 0.09 | 0.29 | -1.41 | -0.54 | 1.04 | 0.54 | increased in CSA-Pi9_S |
| NM | LOC_Os09g02270.1 | Q6YX89 | cyclase family protein, putative | -1.33 | -1.05 | 1.14 | -0.09 | 0.90 | 0.43 | increased in CSA-Pi9_S |
| NM | LOC_Os07g30170.1 | Q8H530 | nitrilase, putative | -1.02 | -1.47 | 1.38 | 0.44 | 0.26 | 0.41 | increased in CSA-Pi9_S |
| NM | LOC_Os08g24760.1 | B9G0E3 | ATP-dependent RNA helicase, putative | -0.18 | -1.06 | -1.10 | 0.79 | 1.21 | 0.34 | increased in CSA-Pi9_S |
| NM | LOC_Os09g39440.1 | Q652Q8 | inosine-uridine preferring nucleoside hydrolase family protein, putative | -0.92 | -1.67 | 1.01 | 0.67 | 0.62 | 0.29 | increased in CSA-Pi9_S |
| NM | LOC_Os05g28180.1 | Q84NP7 | AMP deaminase, putative | -0.49 | -1.32 | -0.11 | 1.19 | 0.70 | 0.04 | increased in CSA-Pi9_S |
| NM | LOC_Os03g31170.1 | Q84MH2 | inosine-uridine preferring nucleoside hydrolase family protein, putative | -1.05 | -1.48 | 1.02 | 0.89 | 0.59 | 0.04 | increased in CSA-Pi9_S |
| NM | LOC_Os11g25330.1 | Q53KM8 | Uncharacterized protein | -0.95 | -0.74 | 1.17 | 1.21 | -0.30 | -0.39 | increased in CSA-Pi9_S |
| AM | LOC_Os08g15030.1 | Q9LD61 | aspartate carbamoyltransferase, putative | -0.18 | -1.21 | -0.08 | -0.30 | 0.63 | 1.14 | increased in CSA-Pi9_S |
| AM | LOC_Os08g36320.2 | Q9AQU4 | decarboxylase, putative | -1.47 | -1.09 | 0.54 | 0.70 | 0.21 | 1.12 | increased in CSA-Pi9_S |
| AM | LOC_Os01g65410.1 | Q8RYY6 | serine hydroxymethyltransferase, mitochondrial precursor, putative | -1.40 | -0.80 | 0.93 | 0.33 | -0.12 | 1.06 | increased in CSA-Pi9_S |
| AM | LOC_Os11g14950.1 | Q53L28 | ACT domain containing protein | -1.52 | -1.01 | 0.75 | 0.43 | 0.33 | 1.02 | increased in CSA-Pi9_S |
| AM | LOC_Os04g01590.1 | Q7X7N2 | arginase, putative | -1.37 | -1.15 | -0.01 | 1.00 | 0.57 | 0.97 | increased in CSA-Pi9_S |
| AM | LOC_Os01g09000.1 | Q5VRX8 | glutaminyl-tRNA synthetase, putative | -0.99 | -1.49 | -0.01 | 0.39 | 1.16 | 0.94 | increased in CSA-Pi9_S |
| AM | LOC_Os03g51080.1 | Q6ASV4 | glutamate decarboxylase, putative | -0.51 | -1.77 | 0.39 | -0.12 | 1.10 | 0.90 | increased in CSA-Pi9_S |
| AM | LOC_Os04g46370.1 | Q0JB91 | Uncharacterized protein | -0.97 | -1.41 | 0.55 | 0.46 | 0.74 | 0.63 | increased in CSA-Pi9_S |
| AM | LOC_Os03g11120.1 | Q10Q53 | methionyl-tRNA synthetase, putative | -0.91 | -1.27 | 0.07 | 1.03 | 0.49 | 0.59 | increased in CSA-Pi9_S |
| AM | LOC_Os02g02860.1 | Q6YUS0 | glutamyl-tRNA synthetase, putative | -1.64 | -0.90 | 0.49 | 0.98 | 0.50 | 0.57 | increased in CSA-Pi9_S |
| AM | LOC_Os03g18130.1 | Q10MX3 | asparagine synthetase, putative | -1.53 | -1.17 | 0.55 | 0.71 | 0.92 | 0.52 | increased in CSA-Pi9_S |
| AM | LOC_Os09g02270.1 | Q6YX89 | cyclase family protein, putative | -1.33 | -1.05 | 1.14 | -0.09 | 0.90 | 0.43 | increased in CSA-Pi9_S |
| AM | LOC_Os07g30170.1 | Q8H530 | nitrilase, putative | -1.02 | -1.47 | 1.38 | 0.44 | 0.26 | 0.41 | increased in CSA-Pi9_S |
| AM | LOC_Os04g37500.1 | Q7XV18 | glutamate decarboxylase, putative | -0.27 | -1.88 | 0.92 | -0.12 | 0.97 | 0.38 | increased in CSA-Pi9_S |
| AM | LOC_Os04g31960.1 | Q7XKQ8 | Uncharacterized protein | -1.17 | -1.43 | 0.63 | 0.77 | 0.84 | 0.37 | increased in CSA-Pi9_S |
| AM | LOC_Os02g33500.1 | Q6ER90 | threonyl-tRNA synthetase, putative | 0.36 | -1.78 | 0.46 | 0.82 | -0.17 | 0.31 | increased in CSA-Pi9_S |
| AM | LOC_Os01g06660.1 | Q9LGL5 | thiamine pyrophosphate enzyme, C-terminal TPP binding domain containing protein | -1.16 | -1.35 | 1.22 | 0.68 | 0.40 | 0.20 | increased in CSA-Pi9_S |
| T | LOC_Os04g16740.1 | Q7DNB0 | ATP synthase subunit alpha, putative | -0.60 | -1.41 | -0.05 | 0.37 | -0.06 | 1.76 | increased in CSA-Pi9_S |
| T | LOC_Os01g40470.1 | Q8RU33 | vacuolar ATP synthase subunit d, putative | -0.35 | -0.57 | -0.93 | -0.77 | 0.94 | 1.68 | increased in CSA-Pi9_S |
| T | LOC_Os07g44040.1 | Q8LHP5 | ras-related protein, putative | -1.25 | -0.67 | 0.02 | -0.35 | 0.62 | 1.64 | increased in CSA-Pi9_S |
| T | LOC_Os01g21820.1 | Q657P0 | pretranslocase subunit secA, chloroplast precursor, putative | -0.95 | -0.94 | -0.79 | 0.36 | 0.79 | 1.53 | increased in CSA-Pi9_S |
| T | LOC_Os05g02060.1 | Q65XV6 | mitochondrial import inner membrane translocase subunit Tim17, putative | 0.40 | -0.45 | -0.90 | -1.15 | 0.58 | 1.52 | increased in CSA-Pi9_S |
| T | LOC_Os03g63250.1 | Q84LH7 | brefeldin A-sensitive Golgi protein-like, putative | 0.05 | -0.01 | -1.52 | -0.70 | 0.69 | 1.49 | increased in CSA-Pi9_S |
| T | LOC_Os02g44080.1 | Q7XA61 | aquaporin protein, putative | 0.11 | -1.20 | -0.05 | -1.12 | 0.79 | 1.46 | increased in CSA-Pi9_S |
| T | LOC_Os07g33954.1 | Q7F1T0 | transporter family protein, putative | -0.84 | -1.19 | -0.15 | -0.37 | 1.12 | 1.43 | increased in CSA-Pi9_S |
| T | LOC_Os06g08110.1 | Q8H613 | nodulin, putative | -0.11 | -1.14 | 0.30 | -1.04 | 0.62 | 1.37 | increased in CSA-Pi9_S |
| T | LOC_Os05g43360.1 | Q6L4W7 | NADH dehydrogenase flavo2, mitochondrial precursor, putative | -1.34 | -1.00 | -0.28 | 0.69 | 0.57 | 1.35 | increased in CSA-Pi9_S |
| T | LOC_Os03g02380.1 | Q8GZW6 | major facilitator superfamily domain-containing 5, putative | 0.19 | -1.22 | 0.17 | -1.19 | 0.71 | 1.34 | increased in CSA-Pi9_S |
| T | LOC_Os03g48940.1 | Q851D8 | chloride channel CLC-d, putative | -0.97 | -1.40 | 0.61 | -0.13 | 0.57 | 1.33 | increased in CSA-Pi9_S |
| T | LOC_Os06g35814.1 | P40393 | ras-related protein, putative | -0.43 | -0.60 | -0.67 | -0.80 | 1.19 | 1.32 | increased in CSA-Pi9_S |
| T | LOC_Os02g21250.1 | Q6Z844 | clathrin adaptor complex small chain domain containing protein | -0.41 | -0.12 | -1.39 | -0.57 | 1.17 | 1.32 | increased in CSA-Pi9_S |
| T | LOC_Os10g23100.1 | Q338Z5 | ras-related protein, putative | -0.54 | -0.61 | -0.50 | -0.90 | 1.24 | 1.31 | increased in CSA-Pi9_S |
| T | LOC_Os01g03144.1 | Q9ARU4 | white-brown complex homolog 7, putative | -0.54 | -1.40 | -0.16 | -0.37 | 1.17 | 1.30 | increased in CSA-Pi9_S |
| T | LOC_Os05g01480.1 | Q75M17 | ras-related protein, putative | -1.11 | -1.16 | -0.19 | 0.18 | 1.02 | 1.26 | increased in CSA-Pi9_S |
| T | LOC_Os05g48980.1 | Q6AUF8 | ras-related protein, putative | -0.19 | -0.62 | -0.82 | -1.01 | 1.38 | 1.26 | increased in CSA-Pi9_S |
| T | LOC_Os05g23720.1 | Q0DJC0 | mitochondrial carrier protein, putative | -1.38 | -1.10 | 0.11 | 0.23 | 0.89 | 1.25 | increased in CSA-Pi9_S |
| T | LOC_Os12g43550.2 | Q0ILQ6 | ras-related protein, putative | 0.13 | -0.66 | -0.76 | -1.07 | 1.15 | 1.21 | increased in CSA-Pi9_S |
| T | LOC_Os12g12370.1 | Q2QVL8 | outer envelope protein, putative | -0.40 | -1.63 | 0.65 | 0.02 | 0.17 | 1.20 | increased in CSA-Pi9_S |
| T | LOC_Os05g47980.1 | A0A067Y2M2 | ATP synthase, putative | -1.23 | -1.03 | 0.79 | 0.48 | -0.17 | 1.15 | increased in CSA-Pi9_S |
| T | LOC_Os03g60870.1 | Q852B4 | ras-related protein, putative | -0.10 | -1.15 | -0.38 | -0.81 | 1.31 | 1.13 | increased in CSA-Pi9_S |
| T | LOC_Os06g43660.1 | Q67WN5 | inorganic H+ pyrophosphatase, putative | -0.55 | -1.55 | 0.40 | -0.52 | 1.08 | 1.13 | increased in CSA-Pi9_S |
| T | LOC_Os08g43470.1 | Q6YT06 | ER lumen retaining receptor, putative | 0.46 | -1.13 | -0.99 | -0.64 | 1.19 | 1.12 | increased in CSA-Pi9_S |
| T | LOC_Os03g53140.1 | Q75KX1 | transport Sec23B, putative | -0.66 | -0.99 | -0.42 | -0.27 | 1.22 | 1.11 | increased in CSA-Pi9_S |
| T | LOC_Os07g45090.1 | Q8H2T7 | NADH-ubiquinone oxidoreductase 51 kDa subunit, mitochondrial precursor, putative | -0.88 | -1.68 | 0.62 | 0.54 | 0.32 | 1.09 | increased in CSA-Pi9_S |
| T | LOC_Os03g56300.1 | Q7XZW1 | 4Fe-4S binding domain containing protein | -0.85 | -0.67 | -1.20 | 0.56 | 1.08 | 1.07 | increased in CSA-Pi9_S |
| T | LOC_Os08g08840.1 | Q6YZC3 | phosphate/phosphate translocator, putative | -0.34 | -1.72 | 0.38 | -0.42 | 1.03 | 1.07 | increased in CSA-Pi9_S |
| T | LOC_Os03g05390.1 | Q10RS7 | Citrate transporter protein, putative | 0.18 | -1.67 | 0.43 | -0.80 | 0.80 | 1.07 | increased in CSA-Pi9_S |
| T | LOC_Os10g39440.1 | Q336W8 | transporter family protein, putative | -0.64 | -1.57 | 1.16 | -0.32 | 0.34 | 1.02 | increased in CSA-Pi9_S |
| T | LOC_Os03g20750.1 | Q10M45 | outer mitochondrial membrane porin, putative | -0.74 | -1.58 | 0.22 | 0.00 | 1.08 | 1.02 | increased in CSA-Pi9_S |
| T | LOC_Os03g56190.1 | Q7XZW4 | Uncharacterized protein | -1.26 | -1.35 | 0.42 | 0.38 | 0.79 | 1.01 | increased in CSA-Pi9_S |
| T | LOC_Os10g37520.1 | Q9FWD6 | major facilitator superfamily domain-containing 5, putative | -0.45 | -1.86 | 0.34 | 0.28 | 0.71 | 0.98 | increased in CSA-Pi9_S |
| T | LOC_Os05g27820.1 | Q5W6M0 | patellin protein, putative | -0.76 | -1.03 | -0.68 | -0.02 | 1.52 | 0.98 | increased in CSA-Pi9_S |
| T | LOC_Os01g15010.1 | Q9SDK4 | miro, putative | -0.27 | -1.31 | -0.37 | -0.49 | 1.51 | 0.93 | increased in CSA-Pi9_S |
| T | LOC_Os09g14520.1 | Q69M22 | integral membrane protein, putative | -0.65 | -1.57 | 0.72 | -0.04 | 0.64 | 0.89 | increased in CSA-Pi9_S |
| T | LOC_Os02g57720.1 | Q9SXF8 | aquaporin protein, putative | -0.93 | -1.68 | 0.74 | 0.27 | 0.75 | 0.85 | increased in CSA-Pi9_S |
| T | LOC_Os04g52900.1 | Q7XTT6 | ABC transporter family protein, putative | -0.48 | -1.82 | 0.98 | -0.14 | 0.63 | 0.83 | increased in CSA-Pi9_S |
| T | LOC_Os10g33170.1 | Q7XDJ1 | Uncharacterized protein | -0.47 | -1.73 | 0.40 | 0.10 | 0.87 | 0.83 | increased in CSA-Pi9_S |
| T | LOC_Os06g43850.1 | Q67W57 | ATP synthase F1, delta subunit family protein, putative | 0.29 | -1.26 | -0.74 | -0.61 | 1.51 | 0.81 | increased in CSA-Pi9_S |
| T | LOC_Os03g17740.1 | Q8GZZ6 | transporter, putative | 0.85 | -1.66 | 0.32 | -0.49 | 0.19 | 0.79 | increased in CSA-Pi9_S |
| T | LOC_Os07g37250.1 | Q84PB7 | THYLAKOID FORMATION1, chloroplast precursor, putative | -0.67 | -0.90 | -0.69 | 0.64 | 0.86 | 0.76 | increased in CSA-Pi9_S |
| T | LOC_Os03g06720.1 | Q10RD6 | Uncharacterized protein | 0.78 | -1.56 | 0.14 | -0.41 | 0.32 | 0.74 | increased in CSA-Pi9_S |
| T | LOC_Os01g48680.1 | Q5QM84 | two pore calcium channel 1, putative | 1.26 | -1.27 | -0.27 | -0.38 | -0.06 | 0.72 | increased in CSA-Pi9_S |
| T | LOC_Os05g41480.1 | Q65X78 | domain of unknown function, DUF250 domain containing protein | -0.75 | -1.58 | 0.47 | -0.16 | 1.35 | 0.67 | increased in CSA-Pi9_S |
| T | LOC_Os08g40530.1 | B9G1S8 | calcium-transporting ATPase 9, plasma membrane-type, putative | -0.89 | -1.05 | 1.05 | -0.65 | 0.89 | 0.64 | increased in CSA-Pi9_S |
| T | LOC_Os09g21000.1 | Q6H4R6 | potassium transporter, putative | -0.58 | -1.74 | 0.98 | 0.16 | 0.54 | 0.64 | increased in CSA-Pi9_S |
| T | LOC_Os07g47350.1 | Q8H3P9 | potassium transporter, putative | -0.78 | -1.64 | 0.95 | 0.02 | 0.81 | 0.64 | increased in CSA-Pi9_S |
| T | LOC_Os02g49980.1 | Q6Z2K0 | ADP-ribosylation factor, putative | -0.26 | -0.85 | -0.97 | -0.22 | 1.68 | 0.62 | increased in CSA-Pi9_S |
| T | LOC_Os07g32530.1 | Q69RI8 | potassium transporter, putative | -0.75 | -1.65 | 0.80 | 0.59 | 0.46 | 0.55 | increased in CSA-Pi9_S |
| T | LOC_Os12g34094.1 | Q8HCP7 | NADH-ubiquinone oxidoreductase chain 4, putative | -0.96 | -1.31 | 1.59 | 0.35 | -0.17 | 0.49 | increased in CSA-Pi9_S |
| T | LOC_Os05g50280.1 | Q688U6 | transporter family protein, putative | -0.01 | -1.75 | 1.24 | -0.28 | 0.31 | 0.49 | increased in CSA-Pi9_S |
| T | LOC_Os06g16420.1 | Q9FNZ8 | Uncharacterized protein | 0.25 | -0.94 | 0.83 | -1.34 | 0.74 | 0.46 | increased in CSA-Pi9_S |
| T | LOC_Os10g42870.1 | Q7XBS8 | peptide transporter PTR2, putative | -0.03 | -1.50 | 1.11 | -0.79 | 0.79 | 0.43 | increased in CSA-Pi9_S |
| T | LOC_Os01g60230.1 | Q0JI87 | auxin efflux carrier component, putative | 0.20 | -1.63 | -0.04 | 0.29 | 0.82 | 0.36 | increased in CSA-Pi9_S |
| T | LOC_Os10g30790.1 | Q8H6G8 | inorganic phosphate transporter, putative | 0.71 | -1.85 | 0.66 | -0.67 | 0.81 | 0.34 | increased in CSA-Pi9_S |
| T | LOC_Os08g05910.1 | Q6ZJR2 | peptide transporter PTR2, putative | -0.05 | -1.57 | 1.05 | -0.80 | 1.08 | 0.30 | increased in CSA-Pi9_S |
| T | LOC_Os02g51810.1 | Q6Z697 | Uncharacterized protein | -0.51 | -1.63 | 0.79 | 0.96 | 0.12 | 0.28 | increased in CSA-Pi9_S |
| T | LOC_Os12g04220.1 | Q2QY12 | calcium-transporting ATPase, plasma membrane-type, putative | -0.66 | -1.79 | 0.98 | 0.69 | 0.51 | 0.27 | increased in CSA-Pi9_S |
| T | LOC_Os01g70940.1 | Q942X8 | potassium transporter, putative | -0.09 | -1.88 | 1.00 | -0.09 | 0.87 | 0.20 | increased in CSA-Pi9_S |
| T | LOC_Os06g08310.1 | Q5SMI5 | Uncharacterized protein | -0.84 | -0.65 | 1.43 | -0.34 | 0.20 | 0.19 | increased in CSA-Pi9_S |
| T | LOC_Os08g28170.1 | Q0J648 | Uncharacterized protein | 0.21 | -1.45 | -0.13 | 0.14 | 1.04 | 0.18 | increased in CSA-Pi9_S |
| T | LOC_Os01g45830.1 | Q9ASA0 | sulfate transporter, putative | -0.61 | -1.58 | 1.54 | 0.28 | 0.22 | 0.15 | increased in CSA-Pi9_S |
| T | LOC_Os01g56420.1 | Q94EE4 | ctr copper transporter family protein, putative | -1.02 | -0.79 | 1.45 | 0.18 | 0.03 | 0.14 | increased in CSA-Pi9_S |
| T | LOC_Os09g02214.1 | A0A0P0XJN2 | Citrate transporter protein, putative | -0.43 | -1.44 | 0.78 | 0.94 | 0.04 | 0.10 | increased in CSA-Pi9_S |
| T | LOC_Os06g05160.1 | Q5VQ79 | Uncharacterized protein | -0.76 | -1.54 | 1.03 | 1.08 | 0.14 | 0.06 | increased in CSA-Pi9_S |
| T | LOC_Os11g17970.1 | Q2R733 | Uncharacterized protein | -0.17 | -1.50 | 0.93 | -0.50 | 1.20 | 0.05 | increased in CSA-Pi9_S |
| T | LOC_Os07g26690.1 | B7EKW3 | aquaporin protein, putative | -0.41 | -1.81 | 0.94 | 0.31 | 0.97 | 0.00 | increased in CSA-Pi9_S |
| T | LOC_Os05g25550.1 | B9FNV9 | E1-E2 ATPase domain containing protein | -0.27 | -1.74 | 1.35 | 0.06 | 0.61 | -0.01 | increased in CSA-Pi9_S |
| T | LOC_Os05g35410.1 | H9BAN2 | potassium channel AKT2/3, putative | -0.04 | -1.36 | 1.42 | -0.28 | 0.40 | -0.13 | increased in CSA-Pi9_S |
| T | LOC_Os05g49270.1 | Q688W0 | transporter family protein, putative | 0.31 | -1.73 | 1.30 | -0.31 | 0.55 | -0.13 | increased in CSA-Pi9_S |
| T | LOC_Os02g46860.1 | B9F1X8 | oligopeptide transporter 4, putative | -0.49 | -1.67 | 1.18 | 0.19 | 0.94 | -0.15 | increased in CSA-Pi9_S |
| T | LOC_Os08g03350.1 | Q6ZK52 | amino acid transporter, putative | 0.76 | -1.55 | 0.61 | -0.82 | 1.15 | -0.15 | increased in CSA-Pi9_S |
| T | LOC_Os02g55890.1 | Q75U53 | inorganic H+ pyrophosphatase, putative | -0.03 | -1.68 | 1.40 | 0.22 | 0.30 | -0.22 | increased in CSA-Pi9_S |
| LM | LOC_Os08g44360.1 | Q6ZJ06 | male sterility 2, putative | -0.62 | -1.64 | 0.02 | 0.21 | 0.82 | 1.21 | increased in CSA-Pi9_S |
| LM | LOC_Os11g39220.1 | Q2R1G8 | acyl-coenzyme A oxidase, putative | -0.85 | -1.33 | 0.03 | -0.17 | 1.16 | 1.15 | increased in CSA-Pi9_S |
| LM | LOC_Os11g35710.1 | Q0IS49 | Uncharacterized protein | -0.09 | -1.34 | -0.11 | -0.56 | 0.97 | 1.13 | increased in CSA-Pi9_S |
| LM | LOC_Os04g28620.1 | Q7XRZ6 | male sterility protein, putative | -1.10 | -1.37 | 0.72 | -0.11 | 0.76 | 1.10 | increased in CSA-Pi9_S |
| LM | LOC_Os10g39930.1 | Q336V3 | cytochrome P450 97B2, putative | -0.54 | -1.51 | -0.59 | 0.66 | 0.87 | 1.09 | increased in CSA-Pi9_S |
| LM | LOC_Os06g24704.1 | Q69XR7 | acyl-coenzyme A oxidase, putative | -0.55 | -1.75 | 0.19 | 0.03 | 1.02 | 1.07 | increased in CSA-Pi9_S |
| LM | LOC_Os03g62740.1 | Q75LC6 | GDSL-like lipase/acylhydrolase, putative | -1.08 | -1.15 | 1.29 | 0.11 | -0.21 | 1.04 | increased in CSA-Pi9_S |
| LM | LOC_Os05g38590.1 | Q0DHK0 | Uncharacterized protein | -0.83 | -1.69 | 0.65 | 0.43 | 0.47 | 0.95 | increased in CSA-Pi9_S |
| LM | LOC_Os04g57850.1 | Q7XQ93 | AMP-binding enzyme, putative | -0.26 | -1.77 | 0.40 | -0.28 | 1.02 | 0.89 | increased in CSA-Pi9_S |
| LM | LOC_Os01g05694.1 | Q5ZEJ0 | hypothetical protein | -0.18 | -1.84 | 0.18 | 0.28 | 0.69 | 0.87 | increased in CSA-Pi9_S |
| LM | LOC_Os01g03750.1 | Q5ZEG0 | Uncharacterized protein | 0.00 | -1.69 | -0.29 | 0.53 | 0.59 | 0.86 | increased in CSA-Pi9_S |
| LM | LOC_Os05g49290.1 | Q688V9 | 3-ketoacyl-CoA synthase, putative | -1.18 | -1.42 | 0.80 | 0.17 | 0.80 | 0.83 | increased in CSA-Pi9_S |
| LM | LOC_Os02g09220.1 | Q6ZIH6 | cytochrome P450, putative | -0.93 | -1.41 | 0.44 | -0.06 | 1.15 | 0.81 | increased in CSA-Pi9_S |
| LM | LOC_Os03g49350.1 | Q7G794 | lipozygenase protein, putative | -1.33 | -1.20 | 0.36 | 1.24 | 0.13 | 0.80 | increased in CSA-Pi9_S |
| LM | LOC_Os02g56920.1 | Q6K3D8 | WAX2, putative | -0.52 | -1.72 | 0.24 | -0.03 | 1.25 | 0.77 | increased in CSA-Pi9_S |
| LM | LOC_Os11g47330.1 | Q2QZ86 | ATP-grasp domain containing protein | -1.31 | -1.37 | 0.78 | 0.59 | 0.55 | 0.75 | increased in CSA-Pi9_S |
| LM | LOC_Os04g02640.1 | B7F5B4 | Uncharacterized protein | -0.74 | -1.30 | 0.35 | 0.11 | 0.84 | 0.75 | increased in CSA-Pi9_S |
| LM | LOC_Os11g03050.1 | A0A0N7KSC9 | cytidylyltransferase domain containing protein | -0.22 | -1.73 | 1.02 | -0.36 | 0.55 | 0.74 | increased in CSA-Pi9_S |
| LM | LOC_Os01g55780.1 | Q8LR39 | glycerophosphoryl diester phosphodiesterase family protein, putative | -0.77 | -1.79 | 0.82 | 0.48 | 0.54 | 0.73 | increased in CSA-Pi9_S |
| LM | LOC_Os03g57680.1 | Q852M2 | aldehyde oxidase, putative | 0.80 | -1.69 | 0.88 | -0.63 | -0.10 | 0.73 | increased in CSA-Pi9_S |
| LM | LOC_Os02g44780.1 | Q6ET88 | Uncharacterized protein | -1.18 | -0.99 | 0.05 | 0.35 | 1.06 | 0.72 | increased in CSA-Pi9_S |
| LM | LOC_Os02g21520.1 | Q6EQW2 | chalcone isomerase 3, putative | -1.33 | -1.35 | 0.57 | 0.84 | 0.57 | 0.71 | increased in CSA-Pi9_S |
| LM | LOC_Os05g33600.1 | A0A0P0WM73 | cytochrome P450 72A1, putative | -0.98 | -1.58 | 0.80 | 0.27 | 0.79 | 0.69 | increased in CSA-Pi9_S |
| LM | LOC_Os03g17520.1 | Q8H8U0 | CDP-alcohol phosphatidyltransferase, putative | 0.07 | -1.60 | 0.13 | -0.64 | 1.36 | 0.68 | increased in CSA-Pi9_S |
| LM | LOC_Os03g12030.1 | Q10PV5 | 3-ketoacyl-CoA synthase, putative | -0.32 | -1.58 | 1.15 | -0.24 | 0.34 | 0.64 | increased in CSA-Pi9_S |
| LM | LOC_Os04g54200.1 | Q7XQT2 | diacylglycerol kinase, putative | -1.03 | -1.57 | 1.09 | 0.32 | 0.56 | 0.63 | increased in CSA-Pi9_S |
| LM | LOC_Os01g70380.1 | Q5JK39 | serine palmitoyltransferase 2, putative | -0.65 | -1.30 | 1.21 | -0.09 | 0.33 | 0.52 | increased in CSA-Pi9_S |
| LM | LOC_Os02g09240.1 | Q6ZIH2 | cytochrome P450 71D8, putative | -1.26 | -1.41 | 0.91 | 0.41 | 0.84 | 0.50 | increased in CSA-Pi9_S |
| LM | LOC_Os11g41900.1 | Q2R0P3 | acyltransferase, putative | -0.59 | -1.74 | 0.31 | 0.21 | 1.31 | 0.48 | increased in CSA-Pi9_S |
| LM | LOC_Os04g01810.1 | Q7X8W2 | terpene synthase, putative | -1.34 | -1.24 | 0.93 | 0.08 | 1.09 | 0.48 | increased in CSA-Pi9_S |
| LM | LOC_Os05g33840.1 | O22567 | transketolase, putative | -1.33 | -1.33 | 0.79 | 0.92 | 0.49 | 0.45 | increased in CSA-Pi9_S |
| LM | LOC_Os01g08810.1 | Q9LGS7 | Uncharacterized protein | -0.44 | -1.20 | 1.29 | -0.27 | 0.16 | 0.45 | increased in CSA-Pi9_S |
| LM | LOC_Os01g49760.1 | Q0JK71 | Uncharacterized protein | -1.33 | -1.28 | 0.51 | 0.80 | 0.94 | 0.36 | increased in CSA-Pi9_S |
| LM | LOC_Os03g50070.1 | Q10E47 | DUF1295 domain containing protein, putative | -0.86 | -1.14 | 1.24 | 0.86 | -0.43 | 0.34 | increased in CSA-Pi9_S |
| LM | LOC_Os02g57290.1 | Q0DWE8 | cytochrome P450, putative | -0.78 | -1.74 | 0.72 | 0.80 | 0.70 | 0.31 | increased in CSA-Pi9_S |
| LM | LOC_Os03g30130.1 | Q75IX4 | phospholipase C, putative | -1.25 | -1.19 | 1.01 | 0.57 | 0.57 | 0.29 | increased in CSA-Pi9_S |
| LM | LOC_Os05g49840.1 | Q6F357 | Uncharacterized protein | -1.17 | -1.23 | 1.47 | 0.24 | 0.50 | 0.20 | increased in CSA-Pi9_S |
| LM | LOC_Os09g19650.1 | Q6K558 | 3-ketoacyl-CoA synthase precursor, putative | -1.23 | -1.21 | 0.98 | 1.20 | 0.07 | 0.18 | increased in CSA-Pi9_S |
| LM | LOC_Os04g27340.1 | A3ASJ4 | terpene synthase, putative | -0.64 | -0.84 | 1.19 | -0.59 | 0.82 | 0.06 | increased in CSA-Pi9_S |
| LM | LOC_Os03g40670.1 | Q6AUZ6 | glycerophosphoryl diester phosphodiesterase family protein, putative | 0.47 | -1.76 | 1.42 | -0.42 | 0.33 | -0.05 | increased in CSA-Pi9_S |
| LM | LOC_Os11g38050.1 | A0A0P0Y3X8 | phosphoesterase family protein, putative | -0.53 | -1.37 | 1.78 | 0.03 | 0.42 | -0.32 | increased in CSA-Pi9_S |
| SM | LOC_Os11g42500.1 | Q2R0I1 | dirigent, putative | -0.02 | 0.66 | -0.96 | -0.72 | -0.73 | 1.76 | increased in CSA-Pi9_S |
| SM | LOC_Os05g34180.1 | Q6AUB0 | hydrolase, NUDIX family, domain containing protein | -0.66 | -0.35 | -1.14 | -0.33 | 1.23 | 1.24 | increased in CSA-Pi9_S |
| SM | LOC_Os08g44360.1 | Q6ZJ06 | male sterility 2, putative | -0.62 | -1.64 | 0.02 | 0.21 | 0.82 | 1.21 | increased in CSA-Pi9_S |
| SM | LOC_Os11g35710.1 | Q0IS49 | Uncharacterized protein | -0.09 | -1.34 | -0.11 | -0.56 | 0.97 | 1.13 | increased in CSA-Pi9_S |
| SM | LOC_Os04g28620.1 | Q7XRZ6 | male sterility protein, putative | -1.10 | -1.37 | 0.72 | -0.11 | 0.76 | 1.10 | increased in CSA-Pi9_S |
| SM | LOC_Os07g36190.1 | Q8GVZ0 | hydrolase, NUDIX family, domain containing protein | -0.49 | -0.72 | -0.79 | -0.65 | 1.66 | 1.00 | increased in CSA-Pi9_S |
| SM | LOC_Os05g46580.1 | Q65XM9 | polyprenyl synthetase, putative | -0.32 | -0.26 | -1.48 | -0.39 | 1.47 | 0.98 | increased in CSA-Pi9_S |
| SM | LOC_Os04g57850.1 | Q7XQ93 | AMP-binding enzyme, putative | -0.26 | -1.77 | 0.40 | -0.28 | 1.02 | 0.89 | increased in CSA-Pi9_S |
| SM | LOC_Os09g23550.1 | Q6ERW7 | dehydrogenase, putative | -1.35 | -1.37 | 0.80 | 0.68 | 0.41 | 0.83 | increased in CSA-Pi9_S |
| SM | LOC_Os03g52040.2 | Q851E7 | OsSCP19 - Putative Serine Carboxypeptidase homologue | -1.51 | -1.19 | 0.76 | 0.45 | 0.72 | 0.78 | increased in CSA-Pi9_S |
| SM | LOC_Os02g56920.1 | Q6K3D8 | WAX2, putative | -0.52 | -1.72 | 0.24 | -0.03 | 1.25 | 0.77 | increased in CSA-Pi9_S |
| SM | LOC_Os02g44780.1 | Q6ET88 | Uncharacterized protein | -1.18 | -0.99 | 0.05 | 0.35 | 1.06 | 0.72 | increased in CSA-Pi9_S |
| SM | LOC_Os02g21520.1 | Q6EQW2 | chalcone isomerase 3, putative | -1.33 | -1.35 | 0.57 | 0.84 | 0.57 | 0.71 | increased in CSA-Pi9_S |
| SM | LOC_Os09g23540.1 | Q6ERW9 | dehydrogenase, putative | -1.31 | -1.42 | 0.78 | 0.72 | 0.56 | 0.67 | increased in CSA-Pi9_S |
| SM | LOC_Os11g07830.1 | Q53NQ9 | dirigent, putative | -1.71 | -0.84 | 0.97 | 0.54 | 0.40 | 0.63 | increased in CSA-Pi9_S |
| SM | LOC_Os01g67530.1 | A0A0P0VBX1 | AMP-binding enzyme, putative | -1.43 | -1.27 | 0.60 | 0.86 | 0.64 | 0.60 | increased in CSA-Pi9_S |
| SM | LOC_Os10g29470.1 | Q337Y2 | dehydrogenase, putative | -1.10 | -1.58 | 0.86 | 0.52 | 0.73 | 0.58 | increased in CSA-Pi9_S |
| SM | LOC_Os04g01810.1 | Q7X8W2 | terpene synthase, putative | -1.34 | -1.24 | 0.93 | 0.08 | 1.09 | 0.48 | increased in CSA-Pi9_S |
| SM | LOC_Os05g33840.1 | O22567 | transketolase, putative | -1.33 | -1.33 | 0.79 | 0.92 | 0.49 | 0.45 | increased in CSA-Pi9_S |
| SM | LOC_Os03g12270.1 | Q10PS6 | Uncharacterized protein | -1.24 | -1.17 | 0.88 | 0.73 | 0.46 | 0.34 | increased in CSA-Pi9_S |
| SM | LOC_Os03g13390.2 | Q10PE7 | oxidoreductase, aldo/keto reductase family protein, putative | -1.09 | -1.34 | 0.42 | 0.97 | 0.82 | 0.22 | increased in CSA-Pi9_S |
| SM | LOC_Os12g16290.1 | Q2QUH0 | isoflavone reductase, putative | -1.17 | -1.15 | 1.10 | 0.92 | 0.14 | 0.16 | increased in CSA-Pi9_S |
| SM | LOC_Os01g63200.1 | Q0JHP8 | laccase precursor protein, putative | -0.41 | -1.62 | 1.33 | 0.31 | 0.25 | 0.14 | increased in CSA-Pi9_S |
| SM | LOC_Os09g23560.1 | Q6ERW5 | dehydrogenase, putative | -1.04 | -1.45 | 1.24 | 0.81 | 0.32 | 0.12 | increased in CSA-Pi9_S |
| SM | LOC_Os04g27340.1 | A3ASJ4 | terpene synthase, putative | -0.64 | -0.84 | 1.19 | -0.59 | 0.82 | 0.06 | increased in CSA-Pi9_S |
| HM | LOC_Os03g57680.1 | Q852M2 | aldehyde oxidase, putative | 0.80 | -1.69 | 0.88 | -0.63 | -0.10 | 0.73 | increased in CSA-Pi9_S |
| HM | LOC_Os01g38580.1 | Q93VD5 | Uncharacterized protein | -1.57 | -1.13 | 0.76 | 0.72 | 0.54 | 0.68 | increased in CSA-Pi9_S |
| HM | LOC_Os12g44310.2 | Q2QLJ0 | carotenoid cleavage dioxygenase, putative | -0.51 | -1.79 | 1.12 | 0.70 | 0.44 | 0.04 | increased in CSA-Pi9_S |
| HM | LOC_Os03g49350.1 | Q7G794 | lipozygenase protein, putative | -1.33 | -1.20 | 0.36 | 1.24 | 0.13 | 0.80 | increased in CSA-Pi9_S |
| CM | LOC_Os03g60340.1 | Q10BH4 | leaf senescence related protein, putative | 0.69 | 1.54 | -0.06 | -1.48 | -0.29 | -0.41 | increased in DJ_S |
| CM | LOC_Os12g36810.1 | Q2QNT4 | polygalacturonase, putative | 0.96 | 1.49 | -0.29 | -1.21 | -0.45 | -0.50 | increased in DJ_S |
| CM | LOC_Os02g39330.1 | Q6K8R2 | CHIT1 - Chitinase family precursor | 0.94 | 1.48 | -0.41 | -0.12 | -0.55 | -1.34 | increased in DJ_S |
| CM | LOC_Os03g15020.1 | B7EDJ0 | beta-galactosidase precursor, putative | 1.02 | 1.47 | -0.30 | -0.19 | -0.92 | -1.08 | increased in DJ_S |
| CM | LOC_Os09g31310.1 | Q5U9F2 | Uncharacterized protein | 1.04 | 1.46 | -0.66 | -0.33 | -0.42 | -1.09 | increased in DJ_S |
| CM | LOC_Os04g33740.1 | B5KNJ4 | glycosyl hydrolases, putative | 1.08 | 1.44 | -1.12 | -0.61 | -0.34 | -0.44 | increased in DJ_S |
| CM | LOC_Os07g35520.1 | A3BKR0 | Uncharacterized protein | 1.05 | 1.42 | -0.57 | -0.66 | -0.34 | -0.91 | increased in DJ_S |
| CM | LOC_Os02g06380.1 | A0A0P0VFA5 | plant-specific domain TIGR01627 family protein | 1.25 | 1.40 | -0.27 | -0.84 | -0.90 | -0.63 | increased in DJ_S |
| CM | LOC_Os08g02230.1 | Q6YXT5 | Uncharacterized protein | 1.33 | 1.39 | -0.78 | -0.66 | -0.66 | -0.61 | increased in DJ_S |
| CM | LOC_Os03g11980.1 | Q10PW1 | beta-hexosaminidase precursor, putative | 1.11 | 1.38 | -0.63 | -1.06 | -0.28 | -0.52 | increased in DJ_S |
| CM | LOC_Os01g71810.1 | Q0JG30 | glycosyl hydrolases family 17, putative | 1.17 | 1.37 | -0.46 | -0.20 | -0.51 | -1.38 | increased in DJ_S |
| CM | LOC_Os01g71080.1 | Q8S1V1 | xylanase inhibitor, putative | 1.07 | 1.33 | 0.22 | -1.30 | -0.85 | -0.47 | increased in DJ_S |
| CM | LOC_Os01g16960.1 | Q5NBQ0 | pyruvate kinase, putative | 1.35 | 1.32 | -0.82 | -0.85 | -0.27 | -0.72 | increased in DJ_S |
| CM | LOC_Os03g18140.1 | Q10MX2 | Uncharacterized protein | 1.20 | 1.28 | -0.36 | -1.45 | -0.43 | -0.24 | increased in DJ_S |
| CM | LOC_Os09g07460.1 | Q6ZXK6 | kelch repeat protein, putative | 1.00 | 1.27 | 0.49 | -1.17 | -1.05 | -0.53 | increased in DJ_S |
| CM | LOC_Os03g04060.1 | Q10S66 | CHIT16 - Chitinase family precursor | 1.01 | 1.26 | -0.21 | 0.17 | -0.70 | -1.53 | increased in DJ_S |
| CM | LOC_Os09g32080.1 | Q0J0L1 | CHIT13 - Chitinase family precursor | 1.25 | 1.24 | -0.65 | -1.27 | -0.37 | -0.20 | increased in DJ_S |
| CM | LOC_Os07g35480.1 | Q6YVU7 | Uncharacterized protein | 1.32 | 1.24 | -0.33 | -0.88 | -0.72 | -0.63 | increased in DJ_S |
| CM | LOC_Os05g29990.1 | Q6I683 | NAD dependent epimerase/dehydratase family domain containing protein | 1.42 | 1.22 | -0.20 | -0.94 | -0.69 | -0.80 | increased in DJ_S |
| CM | LOC_Os01g66940.1 | Q0JGZ6 | kinase, pfkB family, putative | 1.35 | 1.20 | -0.41 | -0.05 | -0.95 | -1.14 | increased in DJ_S |
| CM | LOC_Os02g02560.1 | Q6ZGL5 | Uncharacterized protein | 1.41 | 1.15 | -0.65 | -0.61 | -0.75 | -0.55 | increased in DJ_S |
| CM | LOC_Os02g48390.1 | Q6ZFT5 | phosphoribosyl transferase, putative | 1.49 | 1.12 | -0.34 | -0.83 | -0.67 | -0.77 | increased in DJ_S |
| CM | LOC_Os05g07130.1 | Q0DKG9 | 6PF-2-K/Fru-2,6-P2ASE liver isozyme, putative | 1.37 | 1.08 | -0.42 | 0.06 | -0.87 | -1.22 | increased in DJ_S |
| CM | LOC_Os06g02210.1 | Q5VRZ8 | bark storage A precursor, putative | 1.33 | 1.04 | -0.75 | -1.43 | -0.04 | -0.15 | increased in DJ_S |
| CM | LOC_Os10g28080.1 | Q9FYR9 | glycosyl hydrolase, putative | 1.06 | 1.04 | 0.71 | -0.62 | -1.27 | -0.92 | increased in DJ_S |
| CM | LOC_Os06g40640.2 | Q69V57 | fructose-bisphospate aldolase isozyme, putative | 1.16 | 1.03 | -0.31 | 0.48 | -1.35 | -1.02 | increased in DJ_S |
| CM | LOC_Os08g33440.1 | Q6ZKB1 | 2-oxo acid dehydrogenases acyltransferase domain containing protein | -0.30 | 1.00 | 0.14 | 1.35 | -0.80 | -1.39 | increased in DJ_S |
| CM | LOC_Os05g01440.1 | B9FM04 | phosphoribosylformylglycinamidine synthase, putative | 1.53 | 0.96 | -0.21 | -0.35 | -1.06 | -0.87 | increased in DJ_S |
| CM | LOC_Os04g40510.1 | A0A0P0WBG8 | glycosyl hydrolase family 5 protein, putative | 1.43 | 0.93 | -0.98 | -1.28 | -0.14 | 0.04 | increased in DJ_S |
| CM | LOC_Os12g23180.1 | Q2QSR7 | 3-beta hydroxysteroid dehydrogenase/isomerase family protein, putative | 1.65 | 0.88 | -0.37 | -0.19 | -1.06 | -0.92 | increased in DJ_S |
| CM | LOC_Os03g09250.1 | O64437 | inositol-3-phosphate synthase, putative | 1.64 | 0.86 | -0.41 | -0.10 | -1.10 | -0.89 | increased in DJ_S |
| CM | LOC_Os03g21250.1 | Q10LZ0 | glycosyl transferase, family 8, putative | 0.95 | 0.83 | -0.15 | -1.40 | 0.22 | -0.46 | increased in DJ_S |
| CM | LOC_Os02g47020.1 | Q6Z8F4 | phosphoribulokinase/Uridine kinase family protein | 1.21 | 0.83 | 0.07 | 0.44 | -1.35 | -1.20 | increased in DJ_S |
| CM | LOC_Os04g52370.1 | B7EA04 | UTP--glucose-1-phosphate uridylyltransferase, putative | 1.74 | 0.61 | -0.22 | -0.36 | -0.43 | -1.34 | increased in DJ_S |
| CM | LOC_Os11g13870.1 | Q53N85 | plant-specific domain TIGR01627 family protein | 1.20 | 0.57 | 0.04 | -1.78 | -0.18 | 0.15 | increased in DJ_S |
| CM | LOC_Os01g69030.1 | Q0JGK4 | sucrose-phosphate synthase, putative | 1.75 | 0.53 | -0.89 | -1.06 | -0.44 | 0.11 | increased in DJ_S |
| CM | LOC_Os11g13810.1 | Q2R859 | non-lysosomal glucosylceramidase, putative | 1.73 | 0.33 | 0.28 | -1.32 | -0.34 | -0.68 | increased in DJ_S |
| PS | LOC_Os06g40640.2 | Q69V57 | fructose-bisphospate aldolase isozyme, putative | 1.16 | 1.03 | -0.31 | 0.48 | -1.35 | -1.02 | increased in DJ_S |
| PS | LOC_Os02g47020.1 | Q6Z8F4 | phosphoribulokinase/Uridine kinase family protein | 1.21 | 0.83 | 0.07 | 0.44 | -1.35 | -1.20 | increased in DJ_S |
| NM | LOC_Os02g39700.1 | Q6K6X2 | RNA binding protein, putative | -0.18 | 2.02 | -0.35 | -0.09 | -0.38 | -1.02 | increased in DJ_S |
| NM | LOC_Os07g28430.1 | Q6AWX8 | growth regulating factor protein, putative | 0.20 | 1.98 | -0.51 | -0.44 | -0.70 | -0.53 | increased in DJ_S |
| NM | LOC_Os01g34330.1 | Q5QLH2 | BAH domain containing protein | -0.37 | 1.95 | -1.01 | -0.10 | -0.14 | -0.33 | increased in DJ_S |
| NM | LOC_Os04g30890.1 | B9FES7 | MYB family transcription factor, putative | -0.39 | 1.94 | -1.03 | -0.34 | -0.11 | -0.08 | increased in DJ_S |
| NM | LOC_Os07g36940.1 | B9FXV5 | eukaryotic translation initiation factor 4G, putative | 0.07 | 1.94 | -1.10 | -0.14 | -0.45 | -0.32 | increased in DJ_S |
| NM | LOC_Os09g38970.1 | A0A0P0XQE0 | zinc finger family protein, putative | 0.28 | 1.93 | -1.14 | -0.29 | -0.31 | -0.47 | increased in DJ_S |
| NM | LOC_Os08g01840.1 | Q6ZC63 | nucleic acid binding protein, putative | 0.34 | 1.92 | -0.79 | -0.15 | -0.50 | -0.82 | increased in DJ_S |
| NM | LOC_Os05g48810.1 | Q688X8 | dnaJ domain containing protein | -0.24 | 1.89 | -1.27 | -0.40 | -0.25 | 0.26 | increased in DJ_S |
| NM | LOC_Os09g31486.1 | B9G4B3 | DnaK family protein, putative | 0.40 | 1.88 | -0.02 | -0.56 | -0.70 | -1.01 | increased in DJ_S |
| NM | LOC_Os02g02360.1 | Q6Z7B7 | Uncharacterized protein | 0.36 | 1.88 | -0.83 | -0.12 | -0.82 | -0.46 | increased in DJ_S |
| NM | LOC_Os10g34380.1 | Q337J6 | retrotransposon protein, putative, unclassified | -0.07 | 1.87 | -0.59 | -0.12 | -0.11 | -0.99 | increased in DJ_S |
| NM | LOC_Os03g58430.1 | Q851P5 | 40S ribosomal S15, putative | -0.20 | 1.86 | -0.32 | 0.46 | -0.98 | -0.82 | increased in DJ_S |
| NM | LOC_Os03g12020.1 | Q10PV6 | 50S ribosomal L15, chloroplast precursor, putative | -0.51 | 1.84 | -1.04 | 0.47 | -0.57 | -0.20 | increased in DJ_S |
| NM | LOC_Os01g09890.1 | Q5SMX7 | translation initiation factor SUI1, putative | -0.47 | 1.84 | -1.28 | 0.28 | 0.14 | -0.50 | increased in DJ_S |
| NM | LOC_Os02g52250.1 | Q6K8D9 | SKIP/SNW domain containing protein | -0.25 | 1.81 | -1.27 | 0.16 | -0.44 | -0.02 | increased in DJ_S |
| NM | LOC_Os01g07740.1 | B7EQA6 | DEAD-box ATP-dependent RNA helicase 14, putative | -0.39 | 1.77 | 0.09 | 0.03 | -1.03 | -0.48 | increased in DJ_S |
| NM | LOC_Os02g54060.1 | Q6K826 | chaperonin, putative | 0.64 | 1.73 | -0.55 | 0.13 | -1.04 | -0.91 | increased in DJ_S |
| NM | LOC_Os05g40820.1 | Q75KZ3 | ribosomal L24, putative | 0.17 | 1.71 | -0.15 | 0.15 | -0.76 | -1.13 | increased in DJ_S |
| NM | LOC_Os07g43170.1 | Q7XI40 | adenylate kinase, putative | 0.47 | 1.71 | -0.01 | -0.24 | -0.49 | -1.44 | increased in DJ_S |
| NM | LOC_Os02g15900.1 | Q6K241 | 50S ribosomal L21, chloroplast precursor, putative | 0.30 | 1.71 | -1.07 | 0.22 | -0.60 | -0.55 | increased in DJ_S |
| NM | LOC_Os02g14929.1 | Q6K3R8 | T-complex protein, putative | 0.83 | 1.70 | -0.91 | -0.79 | -0.35 | -0.49 | increased in DJ_S |
| NM | LOC_Os07g29320.1 | Q84RU7 | Uncharacterized protein | 0.02 | 1.70 | -1.37 | 0.28 | -0.07 | -0.57 | increased in DJ_S |
| NM | LOC_Os10g10990.1 | Q8LMN8 | transcription initiation factor IIF, alpha subunit domain containing protein | 0.18 | 1.69 | -1.55 | -0.56 | 0.22 | 0.02 | increased in DJ_S |
| NM | LOC_Os03g49210.1 | Q851S7 | Uncharacterized protein | -0.09 | 1.69 | -1.08 | -0.62 | 0.01 | 0.10 | increased in DJ_S |
| NM | LOC_Os01g56300.1 | Q5ZAQ3 | Uncharacterized protein | 0.28 | 1.69 | -1.11 | -0.44 | 0.24 | -0.65 | increased in DJ_S |
| NM | LOC_Os04g16776.1 | P0C497 | chloroplast 50S ribosomal L2, putative | -0.26 | 1.68 | -0.99 | 0.73 | -0.96 | -0.21 | increased in DJ_S |
| NM | LOC_Os12g12360.1 | Q2QVL9 | transposon protein, putative, Mutator sub-class | -0.07 | 1.68 | -1.17 | 0.26 | -0.11 | -0.59 | increased in DJ_S |
| NM | LOC_Os03g62630.1 | Q75LD8 | ribosomal S6, putative | -0.93 | 1.68 | -1.12 | 0.65 | -0.01 | -0.27 | increased in DJ_S |
| NM | LOC_Os02g54470.1 | Q6K8U8 | 60S ribosomal L35a-3, putative | -0.49 | 1.66 | -0.34 | 0.40 | -0.05 | -1.17 | increased in DJ_S |
| NM | LOC_Os07g34589.2 | B7F683 | translation initiation factor SUI1, putative | -0.04 | 1.66 | -1.30 | -0.40 | 0.02 | 0.06 | increased in DJ_S |
| NM | LOC_Os02g37870.1 | Q69L77 | Brix domain containing protein, putative | -0.03 | 1.66 | -0.92 | -0.67 | 0.78 | -0.82 | increased in DJ_S |
| NM | LOC_Os02g13990.1 | Q6EUK2 | U2 small nuclear ribonucleoA, putative | -1.11 | 1.65 | -1.13 | 0.45 | 0.27 | -0.13 | increased in DJ_S |
| NM | LOC_Os02g39890.1 | Q84L14 | RNA recognition motif containing protein, putative | 0.21 | 1.64 | -0.53 | 0.35 | -0.75 | -0.93 | increased in DJ_S |
| NM | LOC_Os01g13080.1 | Q5NB69 | 60S acidic ribosomal protein, putative | -0.49 | 1.61 | -0.79 | 0.85 | -0.07 | -1.11 | increased in DJ_S |
| NM | LOC_Os07g08330.1 | Q6ZLB8 | ribosomal L4, putative | -0.14 | 1.61 | 0.01 | 0.73 | -1.00 | -1.22 | increased in DJ_S |
| NM | LOC_Os09g11250.1 | Q6EPR4 | co-chaperone GrpE protein, putative | -0.19 | 1.61 | -1.12 | 0.47 | -0.39 | -0.38 | increased in DJ_S |
| NM | LOC_Os02g07070.1 | B9F385 | RNA recognition motif family protein | -0.35 | 1.61 | -1.11 | 0.40 | -0.12 | -0.43 | increased in DJ_S |
| NM | LOC_Os03g31090.1 | P40978 | 40S ribosomal S19, putative | -0.87 | 1.60 | 0.08 | 0.76 | -0.52 | -1.06 | increased in DJ_S |
| NM | LOC_Os01g66980.1 | Q5JLV2 | Uncharacterized protein | 0.12 | 1.60 | -1.14 | 0.51 | -0.23 | -0.86 | increased in DJ_S |
| NM | LOC_Os03g44900.1 | Q7XZZ2 | CCR4-NOT transcription factor, putative | 0.13 | 1.59 | 0.00 | 0.50 | -1.04 | -1.18 | increased in DJ_S |
| NM | LOC_Os08g06070.1 | Q6ZD92 | ELF7, putative | -0.23 | 1.59 | -0.47 | 0.62 | -0.98 | -0.53 | increased in DJ_S |
| NM | LOC_Os04g39629.1 | Q7XR25 | Uncharacterized protein | 0.64 | 1.58 | -0.43 | 0.22 | -0.98 | -1.03 | increased in DJ_S |
| NM | LOC_Os02g52360.1 | Q6Z6H8 | peptidyl-prolyl cis-trans isomerase CYP40, putative | 0.44 | 1.58 | -0.02 | -0.25 | -1.40 | -0.34 | increased in DJ_S |
| NM | LOC_Os03g43800.1 | Q10G93 | Uncharacterized protein | -0.78 | 1.57 | -0.56 | -0.01 | 0.45 | -0.66 | increased in DJ_S |
| NM | LOC_Os03g15870.1 | Q10NM5 | ribosomal L4, putative | -0.06 | 1.56 | -0.01 | 0.74 | -1.29 | -0.95 | increased in DJ_S |
| NM | LOC_Os04g02000.1 | B9FD24 | zinc finger family protein, putative | 0.51 | 1.55 | -1.23 | 0.38 | -0.42 | -0.80 | increased in DJ_S |
| NM | LOC_Os03g46640.1 | Q10FF9 | deoxyuridine 5-triphosphate nucleotidohydrolase, putative | 0.96 | 1.55 | -0.58 | 0.02 | -0.80 | -1.16 | increased in DJ_S |
| NM | LOC_Os07g12320.1 | Q8LH68 | WD domain, G-beta repeat domain containing protein | -0.07 | 1.54 | -1.36 | -0.29 | 0.39 | -0.20 | increased in DJ_S |
| NM | LOC_Os08g21840.1 | Q6UUH5 | 50S ribosomal L15, putative | -0.65 | 1.54 | 0.04 | 0.87 | -0.67 | -1.12 | increased in DJ_S |
| NM | LOC_Os08g02410.1 | Q69UI1 | 40S ribosomal S13, putative | -0.71 | 1.53 | -0.45 | 0.81 | -1.00 | -0.18 | increased in DJ_S |
| NM | LOC_Os12g15420.1 | Q2QUQ9 | nucampholin, putative | 0.60 | 1.52 | -1.42 | -0.09 | 0.24 | -0.85 | increased in DJ_S |
| NM | LOC_Os03g08810.1 | Q10QR1 | Uncharacterized protein | -0.36 | 1.52 | 0.11 | -0.23 | -0.95 | -0.08 | increased in DJ_S |
| NM | LOC_Os01g61310.1 | Q941W1 | DUF640 domain containing protein, putative | 0.96 | 1.51 | -0.68 | 0.09 | -0.96 | -0.93 | increased in DJ_S |
| NM | LOC_Os11g45590.1 | B9G8T5 | transposon protein, putative, CACTA, En/Spm sub-class | 0.53 | 1.51 | -1.29 | 0.03 | 0.26 | -1.04 | increased in DJ_S |
| NM | LOC_Os08g25090.1 | Q6Z0G0 | co-chaperone GrpE protein, putative | -0.60 | 1.50 | -0.96 | 1.04 | -0.77 | -0.22 | increased in DJ_S |
| NM | LOC_Os03g49220.1 | Q851S8 | adenylosuccinate synthetase, chloroplast precursor, putative | 1.05 | 1.50 | -0.64 | -0.07 | -0.87 | -0.97 | increased in DJ_S |
| NM | LOC_Os03g21940.1 | Q10LR6 | 60S ribosomal L19-3, putative | 0.25 | 1.50 | -0.30 | 0.70 | -0.79 | -1.37 | increased in DJ_S |
| NM | LOC_Os04g56760.1 | Q7XM15 | PHF5-like domain containing protein | 0.22 | 1.49 | -1.69 | -0.18 | 0.21 | -0.04 | increased in DJ_S |
| NM | LOC_Os03g29260.1 | A3AIY6 | elongation factor protein, putative | 0.24 | 1.46 | -0.16 | 0.76 | -0.92 | -1.38 | increased in DJ_S |
| NM | LOC_Os12g33924.1 | Q7JAI8 | mitochondrial ribosomal S12, putative | 0.45 | 1.42 | -1.41 | -0.24 | -0.34 | 0.11 | increased in DJ_S |
| NM | LOC_Os01g09510.1 | Q5SNH7 | 60S acidic ribosomal protein, putative | -0.69 | 1.42 | -1.30 | 0.82 | 0.34 | -0.59 | increased in DJ_S |
| NM | LOC_Os03g52594.1 | Q10D98 | CCR4-NOT transcription factor, putative | 0.20 | 1.41 | -0.57 | 0.87 | -0.61 | -1.30 | increased in DJ_S |
| NM | LOC_Os12g36194.1 | Q0IMS5 | nucleoside diphosphate kinase, putative | 0.43 | 1.41 | -0.18 | 0.71 | -1.23 | -1.14 | increased in DJ_S |
| NM | LOC_Os03g22180.1 | Q10LN7 | 60S ribosomal L18-3, putative | -0.93 | 1.41 | 0.14 | 1.06 | -0.56 | -1.12 | increased in DJ_S |
| NM | LOC_Os05g08950.1 | A0A0P0WIZ3 | phosphoribosyl transferase, putative | 0.98 | 1.40 | -0.53 | -0.39 | -0.89 | -0.57 | increased in DJ_S |
| NM | LOC_Os12g42150.1 | Q2QM63 | WD domain, G-beta repeat domain containing protein | 0.53 | 1.38 | -0.48 | -0.53 | 0.44 | -1.35 | increased in DJ_S |
| NM | LOC_Os03g63670.1 | Q84T85 | Uncharacterized protein | 1.08 | 1.38 | -1.22 | 0.05 | -0.71 | -0.58 | increased in DJ_S |
| NM | LOC_Os03g17860.1 | Q10N04 | OsPDIL5-1 disulfide isomerase PDIL5-1 | -0.49 | 1.37 | -0.43 | 0.66 | -0.47 | -0.63 | increased in DJ_S |
| NM | LOC_Os01g21180.1 | Q8RZU5 | translation machinery-associated 20, putative | -0.74 | 1.35 | -1.12 | 0.68 | 0.67 | -0.84 | increased in DJ_S |
| NM | LOC_Os02g30624.1 | Q6K2E9 | LSM domain containing protein | -0.36 | 1.35 | -0.62 | 0.65 | -0.21 | -0.82 | increased in DJ_S |
| NM | LOC_Os07g46750.1 | P29545 | elongation factor protein, putative | -0.10 | 1.33 | -0.09 | 1.11 | -0.94 | -1.31 | increased in DJ_S |
| NM | LOC_Os05g48410.1 | Q6I600 | 50S ribosomal L21, chloroplast precursor, putative | -0.35 | 1.33 | 0.25 | 0.52 | -0.21 | -1.53 | increased in DJ_S |
| NM | LOC_Os05g37330.1 | Q65WT5 | 60S acidic ribosomal protein, putative | -0.45 | 1.31 | 0.21 | 1.06 | -1.26 | -0.87 | increased in DJ_S |
| NM | LOC_Os01g08860.1 | E5D3J5 | Uncharacterized protein | 0.83 | 1.30 | -0.33 | -0.74 | -0.62 | -0.45 | increased in DJ_S |
| NM | LOC_Os12g38000.1 | Q2QNF3 | 60S ribosomal L8, putative | -0.05 | 1.29 | 0.17 | 0.66 | -0.56 | -1.50 | increased in DJ_S |
| NM | LOC_Os01g41190.1 | B7FAK7 | Uncharacterized protein | -1.02 | 1.29 | -0.05 | 0.58 | -0.07 | -0.73 | increased in DJ_S |
| NM | LOC_Os02g04460.1 | Q0E446 | ribosomal L3, putative | -0.56 | 1.27 | 0.02 | 1.12 | -0.84 | -1.01 | increased in DJ_S |
| NM | LOC_Os05g01910.1 | B7F8F5 | pumilio-family RNA binding protein, putative | -0.03 | 1.24 | -1.19 | 0.93 | -0.41 | -0.54 | increased in DJ_S |
| NM | LOC_Os08g39440.1 | Q6ZK85 | RNA recognition motif containing protein, putative | 0.56 | 1.18 | -1.61 | 0.14 | 0.20 | -0.46 | increased in DJ_S |
| NM | LOC_Os02g28810.1 | P49199 | ribosomal protein, putative | -0.35 | 1.16 | 0.69 | 0.88 | -1.12 | -1.27 | increased in DJ_S |
| NM | LOC_Os07g47710.1 | Q6YSX0 | 60S ribosomal L22-2, putative | -1.18 | 1.16 | -0.90 | 0.63 | 0.78 | -0.48 | increased in DJ_S |
| NM | LOC_Os05g04940.1 | Q75KG9 | RNA methyltransferase, TrmH family protein, putative | 0.76 | 1.15 | 0.54 | -0.43 | -0.54 | -1.47 | increased in DJ_S |
| NM | LOC_Os02g48390.1 | Q6ZFT5 | phosphoribosyl transferase, putative | 1.49 | 1.12 | -0.34 | -0.83 | -0.67 | -0.77 | increased in DJ_S |
| NM | LOC_Os12g18880.1 | P49030 | mago nashi, putative | 0.40 | 1.10 | -0.67 | 0.77 | 0.11 | -1.71 | increased in DJ_S |
| NM | LOC_Os05g46430.1 | Q5TKP3 | 60S ribosomal L28-1, putative | -0.48 | 1.07 | 0.04 | 1.03 | -0.06 | -1.60 | increased in DJ_S |
| NM | LOC_Os01g08960.1 | Q9LGR1 | phosducin-like 3, putative | -0.38 | 1.04 | 0.64 | 0.91 | -0.88 | -1.33 | increased in DJ_S |
| NM | LOC_Os09g39270.1 | Q0IZJ8 | ZOS9-20 - C2H2 zinc finger protein | 0.08 | 1.00 | 0.30 | 0.83 | -0.68 | -1.53 | increased in DJ_S |
| NM | LOC_Os04g36859.1 | Q7XUX4 | signal peptidase complex subunit 2, putative | -0.37 | 0.98 | 0.51 | 0.93 | -1.14 | -0.91 | increased in DJ_S |
| NM | LOC_Os08g06240.1 | Q84PV0 | Uncharacterized protein | 0.85 | 0.97 | -0.76 | -1.49 | 0.44 | 0.00 | increased in DJ_S |
| NM | LOC_Os05g01440.1 | B9FM04 | phosphoribosylformylglycinamidine synthase, putative | 1.53 | 0.96 | -0.21 | -0.35 | -1.06 | -0.87 | increased in DJ_S |
| NM | LOC_Os12g23180.1 | Q2QSR7 | 3-beta hydroxysteroid dehydrogenase/isomerase family protein, putative | 1.65 | 0.88 | -0.37 | -0.19 | -1.06 | -0.92 | increased in DJ_S |
| NM | LOC_Os07g36254.1 | Q8GVY2 | 60S ribosomal L38, putative | 0.02 | 0.63 | 1.27 | 0.17 | -0.77 | -1.33 | increased in DJ_S |
| AM | LOC_Os05g48450.1 | Q6AT43 | aminotransferase domain containing protein, putative | 0.91 | 1.71 | -0.41 | -0.55 | -0.90 | -0.75 | increased in DJ_S |
| AM | LOC_Os02g07160.1 | Q6H4V1 | glyoxalase family protein, putative | 1.10 | 1.39 | -0.32 | -0.06 | -0.90 | -1.21 | increased in DJ_S |
| AM | LOC_Os04g01690.1 | Q7XRA1 | pyridoxal-dependent decarboxylase protein, putative | 1.13 | 1.32 | -0.27 | -0.07 | -1.44 | -0.67 | increased in DJ_S |
| AM | LOC_Os02g04700.1 | Q6Z2Y0 | tRNA synthetases class II domain containing protein | 1.20 | 1.20 | 0.27 | -1.10 | -0.95 | -0.62 | increased in DJ_S |
| AM | LOC_Os03g42110.1 | Q6AV34 | semialdehyde dehydrogenase, NAD binding domain containing protein, putative | 1.26 | 1.15 | 0.01 | -0.19 | -1.15 | -1.09 | increased in DJ_S |
| AM | LOC_Os02g17330.1 | Q0E243 | aminotransferase domain containing protein, putative | 1.52 | 1.10 | -0.38 | -0.68 | -0.91 | -0.64 | increased in DJ_S |
| AM | LOC_Os06g42560.1 | Q67VM1 | tryptophan synthase beta chain 2, putative | 1.09 | 1.07 | 0.09 | 0.25 | -1.30 | -1.20 | increased in DJ_S |
| AM | LOC_Os02g54254.1 | E3WF37 | saccharopine dehydrogenase, putative | 1.43 | 1.05 | -0.72 | -1.21 | -0.43 | -0.12 | increased in DJ_S |
| AM | LOC_Os06g44460.1 | A3BE72 | D-3-phosphoglycerate dehydrogenase, chloroplast precursor, putative | 1.52 | 0.98 | -0.59 | -1.03 | -0.91 | 0.03 | increased in DJ_S |
| AM | LOC_Os10g31820.1 | Q0IX96 | fluG, putative | 1.63 | 0.98 | -0.69 | -0.82 | -0.81 | -0.30 | increased in DJ_S |
| AM | LOC_Os05g01440.1 | B9FM04 | phosphoribosylformylglycinamidine synthase, putative | 1.53 | 0.96 | -0.21 | -0.35 | -1.06 | -0.87 | increased in DJ_S |
| AM | LOC_Os04g56400.1 | P14655 | glutamine synthetase, catalytic domain containing protein | 1.68 | 0.94 | -0.71 | -0.41 | -0.98 | -0.52 | increased in DJ_S |
| AM | LOC_Os12g33610.1 | A0A0P0YAL3 | Uncharacterized protein | 1.30 | 0.82 | -0.90 | -0.37 | -0.11 | -0.74 | increased in DJ_S |
| AM | LOC_Os10g37340.1 | Q7XCS3 | cystathionine gamma-synthase, putative | 1.15 | 0.78 | -0.27 | -1.68 | -0.46 | 0.48 | increased in DJ_S |
| T | LOC_Os01g61410.1 | B9EU97 | NADH-ubiquinone oxidoreductase, mitochondrial precursor, putative | 1.59 | 0.81 | 0.15 | -0.84 | -0.54 | -1.18 | increased in DJ_S |
| LM | LOC_Os01g16960.1 | Q5NBQ0 | pyruvate kinase, putative | 1.35 | 1.32 | -0.82 | -0.85 | -0.27 | -0.72 | increased in DJ_S |
| LM | LOC_Os02g02700.1 | Q6YUT4 | AMP-binding enzyme, putative | 1.41 | 0.81 | -0.70 | -0.68 | 0.23 | -1.07 | increased in DJ_S |
| LM | LOC_Os01g08760.1 | Q0JQ41 | choline/ethanolamine kinase, putative | 0.76 | 0.78 | 0.00 | -1.12 | 0.59 | -1.00 | increased in DJ_S |
| SM | LOC_Os02g07160.1 | Q6H4V1 | glyoxalase family protein, putative | 1.10 | 1.39 | -0.32 | -0.06 | -0.90 | -1.21 | increased in DJ_S |
| SM | LOC_Os02g46260.1 | B9F1V9 | OsSCP9 - Putative Serine Carboxypeptidase homologue | 1.29 | 1.36 | -0.56 | -1.13 | -0.54 | -0.43 | increased in DJ_S |
| SM | LOC_Os03g16610.1 | Q10ND7 | Uncharacterized protein | 0.60 | 1.18 | 0.21 | -0.88 | 0.26 | -1.38 | increased in DJ_S |
| SM | LOC_Os07g44250.1 | Q7XIX5 | dirigent, putative | 1.39 | 1.05 | -0.30 | -0.39 | -0.92 | -0.83 | increased in DJ_S |
| SM | LOC_Os10g01134.1 | Q10A76 | OsSCP46 - Putative Serine Carboxypeptidase homologue | 1.40 | 0.82 | -0.64 | -1.43 | 0.10 | -0.25 | increased in DJ_S |
| SM | LOC_Os12g33610.1 | A0A0P0YAL3 | Uncharacterized protein | 1.30 | 0.82 | -0.90 | -0.37 | -0.11 | -0.74 | increased in DJ_S |
| SM | LOC_Os02g02700.1 | Q6YUT4 | AMP-binding enzyme, putative | 1.41 | 0.81 | -0.70 | -0.68 | 0.23 | -1.07 | increased in DJ_S |
| HM | LOC_Os09g07460.1 | Q6ZXK6 | kelch repeat protein, putative | 1.00 | 1.27 | 0.49 | -1.17 | -1.05 | -0.53 | increased in DJ_S |
