# Supplementary material for: Comprehensive DIA‐MS Proteomics of Root Basal Nodes Elucidates Mechanisms of Salt Tolerance in Rice
Source: Proteomics. 2026 Apr 29;26(9):140–7. doi: 10.1002/pmic.70138 (PMC13519373; doi:10.1002/pmic.70138)
Supplement: Supplementary file 4 [file PMIC-26--s004.docx]

**Table S4.** List of 40 proteins showing increased abundance in the CSA-Pi9_S sample

| **Cat** | **Locus IDs** | **Uniprot IDs** | **Protein name** | **Protein symbols** | **DJ_C** | **DJ_S** | **CSA_C** | **CSA_S** | **CSA-Pi9_C** | **CSA-Pi9_S** | **FC (CSA-Pi9_S vs CSA-Pi9_C)** | **FC (CSA-Pi9_S vs DJ_S)** |
| --- | --- | --- | --- | --- | --- | --- | --- | --- | --- | --- | --- | --- |
| Sodium exclusion | LOC_Os06g43660.1 | Q67WN5 | AVP1; ATPase/ hydrogen-translocating pyrophosphatase | AVP1 | -0.55 | -1.55 | 0.40 | -0.52 | 1.08 | 1.13 | 1.0125671 | 1.9627731 |
| Sodium exclusion | LOC_Os09g21000.1 | Q6H4R6 | Potassium transporter 23 (High-affinity potassium transporter) | OsHAK23 | -0.58 | -1.74 | 0.98 | 0.16 | 0.54 | 0.64 | 1.0323552 | 2.1365671 |
| Sodium exclusion | LOC_Os07g32530.1 | Q69RI8 | Probable potassium transporter 14 | OsHAK14 | -0.75 | -1.65 | 0.80 | 0.59 | 0.46 | 0.55 | 1.021313 | 1.6477955 |
| Sodium exclusion | LOC_Os02g57720.1 | Q9SXF8 | Aquaporin Plasma membrane intrinsic protein 1-3 | PIP1-3 | -0.93 | -1.68 | 0.74 | 0.27 | 0.75 | 0.85 | 1.0831601 | 6.8680183 |
| Membrane remodeling | LOC_Os01g21820.1 | Q657P0 | Protein translocase subunit SecA | OsSecA | -0.95 | -0.94 | -0.79 | 0.36 | 0.79 | 1.53 | 1.1915788 | 1.7895921 |
| Membrane remodeling | LOC_Os01g55780.1 | Q8LR39 | glycerophosphoryl diester phosphodiesterase family protein | GDPD | -0.77 | -1.79 | 0.82 | 0.48 | 0.54 | 0.73 | 1.060223 | 2.1270578 |
| Membrane remodeling | LOC_Os04g54200.1 | Q7XQT2 | Diacylglycerol kinase 1 | DGK1 | -1.03 | -1.57 | 1.09 | 0.32 | 0.56 | 0.63 | 1.0347743 | 3.0666401 |
| Membrane stabilization | LOC_Os01g70380.1 | Q5JK39 | Long chain base biosynthesis protein 2d (Serine palmitoyltransferase) | LCB2 | -0.65 | -1.30 | 1.21 | -0.09 | 0.33 | 0.52 | 1.047014 | 1.5529233 |
| Membrane stabilization | LOC_Os05g34180.1 | Q6AUB0 | isopentenyl-diphosphate delta-isomerase 1 | IDI1 | -0.66 | -0.35 | -1.14 | -0.33 | 1.23 | 1.24 | 1.00585 | 2.0067681 |
| Membrane stabilization | LOC_Os11g35710.1 | Q0IS49 | Cycloartenol synthase 1 | CAS1 | -0.09 | -1.34 | -0.11 | -0.56 | 0.97 | 1.13 | 1.1453464 | 8.7195917 |
| Membrane stabilization | LOC_Os08g44360.1 | Q6ZJ06 | Fatty acyl-CoA reductase | FAR1 | -0.62 | -1.64 | 0.02 | 0.21 | 0.82 | 1.21 | 1.0590728 | 1.5205081 |
| Membrane stabilization | LOC_Os04g28620.1 | Q7XRZ6 | Fatty acyl-CoA reductase | FAR1 | -1.10 | -1.37 | 0.72 | -0.11 | 0.76 | 1.10 | 1.0835647 | 1.7932137 |
| Cell wall reinforcement | LOC_Os09g23550.1 | Q6ERW7 | cinnamyl alcohol dehydrogenase 8C | CAD8C | -1.35 | -1.37 | 0.80 | 0.68 | 0.41 | 0.83 | 1.1514094 | 2.1023604 |
| Cell wall reinforcement | LOC_Os09g23540.1 | Q6ERW9 | Cinnamyl alcohol dehydrogenase 8B | CAD8B | -1.31 | -1.42 | 0.78 | 0.72 | 0.56 | 0.67 | 1.0771086 | 4.0666583 |
| Cell wall reinforcement | LOC_Os01g67530.1 | A0A0P0VBX1 | 4-coumarate--CoA ligase | 4CL | -1.43 | -1.27 | 0.60 | 0.86 | 0.64 | 0.60 | -1.009704 | 1.6776612 |
| Cuticle and wax reinforcement | LOC_Os01g05694.1 | Q5ZEJ0 | Very-long-chain (3R)-3-hydroxyacyl-CoA dehydratase PASTICCINO 2B | PAS2B | -0.18 | -1.84 | 0.18 | 0.28 | 0.69 | 0.87 | 1.0400302 | 1.8459374 |
| Cuticle and wax reinforcement | LOC_Os05g49290.1 | Q688V9 | 3-ketoacyl-CoA synthase 4 | KCS4 | -1.18 | -1.42 | 0.80 | 0.17 | 0.80 | 0.83 | 1.0122757 | 2.5271223 |
| Cuticle and wax reinforcement | LOC_Os04g02640.1 | B7F5B4 | 3-ketoacyl-CoA synthase 6 | KCS6 | -0.74 | -1.30 | 0.35 | 0.11 | 0.84 | 0.75 | -1.151294 | 27.142727 |
| Cuticle and wax reinforcement | LOC_Os09g34930.1 | Q650T5 | 3-ketoacyl-CoA synthase 4 | KCS4 | -1.69 | -0.57 | 0.81 | 0.57 | 0.18 | 0.71 | 1.2753473 | 1.8015099 |
| Cuticle and wax reinforcement | LOC_Os03g12030.1 | Q10PV5 | 3-ketoacyl-CoA synthase 6 | KCS6 | -0.32 | -1.58 | 1.15 | -0.24 | 0.34 | 0.64 | 1.0948816 | 1.9714962 |
| Energy production | LOC_Os06g24704.1 | Q69XR7 | Acyl-coenzyme A oxidase 3 | ACX3 | -0.55 | -1.75 | 0.19 | 0.03 | 1.02 | 1.07 | 1.0112502 | 1.9772449 |
| Energy production | LOC_Os04g57850.1 | Q7XQ93 | Acyl-activating enzyme 6 | AAE6 | -0.26 | -1.77 | 0.40 | -0.28 | 1.02 | 0.89 | -1.040356 | 2.2487139 |
| Energy production | LOC_Os04g16740.1 | Q7DNB0 | ATP synthase subunit alpha | ATPA | -0.60 | -1.41 | -0.05 | 0.37 | -0.06 | 1.76 | 1.3224689 | 1.6288412 |
| Energy production | LOC_Os03g56300.1 | Q7XZW1 | NADH:ubiquinone oxidoreductase subunit S7 (NADH-ubiquinone oxidoreductase 23 kDa subunit, mitochondrial (TYKY)) | NDUFS7 | -0.85 | -0.67 | -1.20 | 0.56 | 1.08 | 1.07 | -1.001661 | 1.5147761 |
| Energy production | LOC_Os05g43360.1 | Q6L4W7 | NADH:ubiquinone oxidoreductase subunit S8 | NDUFS8 | -1.34 | -1.00 | -0.28 | 0.69 | 0.57 | 1.35 | 1.1983204 | 1.7292424 |
| Energy production | LOC_Os07g45090.1 | Q8H2T7 | NADH:ubiquinone oxidoreductase flavoprotein 1 | NDUFV1 | -0.88 | -1.68 | 0.62 | 0.54 | 0.32 | 1.09 | 1.1289335 | 1.5532371 |
| Energy production | LOC_Os12g34094.1 | Q8HCP7 | NADH-ubiquinone oxidoreductase chain 4 | NAD4 | -0.96 | -1.31 | 1.59 | 0.35 | -0.17 | 0.49 | 1.2174915 | 1.7151566 |
| Energy production | LOC_Os05g23720.1 | Q0DJC0 | ADP/ATP Carrier1 | AAC1 | -1.38 | -1.10 | 0.11 | 0.23 | 0.89 | 1.25 | 1.091745 | 1.7718772 |
| Energy production | LOC_Os11g39220.1 | Q2R1G8 | Acyl-coenzyme A oxidase 2 | ACX2 | -0.85 | -1.33 | 0.03 | -0.17 | 1.16 | 1.15 | -1.002405 | 1.5178626 |
| Metabolic homeostasis | LOC_Os07g33954.1 | Q7F1T0 | Glucose-6-phosphate/phosphate-translocator | GPT2 | -0.84 | -1.19 | -0.15 | -0.37 | 1.12 | 1.43 | 1.1997465 | 4.7898016 |
| Metabolic homeostasis | LOC_Os08g08840.1 | Q6YZC3 | Glucose-6-phosphate/phosphate-translocator | GPT2 | -0.34 | -1.72 | 0.38 | -0.42 | 1.03 | 1.07 | 1.0136328 | 2.8602361 |
| Metabolic homeostasis | LOC_Os10g39440.1 | Q336W8 | Monosaccharide-sensing protein 2 | MSSP2 | -0.64 | -1.57 | 1.16 | -0.32 | 0.34 | 1.02 | 1.1218493 | 1.5431205 |
| Metabolic homeostasis | LOC_Os03g20750.1 | Q10M45 | Translocase of the Outer Mitochondrial Membrane 40 | TOM40 | -0.74 | -1.58 | 0.22 | 0.00 | 1.08 | 1.02 | -1.01221 | 1.6845796 |
| Metabolic homeostasis | LOC_Os05g50280.1 | Q688U6 | Sugar transporter ERD6-like 4 | ERDL4 | -0.01 | -1.75 | 1.24 | -0.28 | 0.31 | 0.49 | 1.0426641 | 1.7051317 |
| Nitrogen regulation | LOC_Os10g33170.1 | Q7XDJ1 | NRT1 (nitrate transporter 1)/PTR (peptide transporter) family 5.2 | NPF5.2 | -0.47 | -1.73 | 0.40 | 0.10 | 0.87 | 0.83 | -1.032881 | 6.394927 |
| Nitrogen regulation | LOC_Os02g44080.1 | Q7XA61 | Tonoplast intrinsic protein 2-1 | TIP2-1 | 0.11 | -1.20 | -0.05 | -1.12 | 0.79 | 1.46 | 1.1827915 | 1.9397003 |
| Nitrogen regulation | LOC_Os03g48940.1 | Q851D8 | Chloride channel protein CLC-d | CLC-D | -0.97 | -1.40 | 0.61 | -0.13 | 0.57 | 1.33 | 1.3062191 | 2.6116521 |
| Misc | LOC_Os12g12370.1 | Q2QVL8 | OEP37 (Outer Envelope Protein 37); ion channel | OEP37 | -0.40 | -1.63 | 0.65 | 0.02 | 0.17 | 1.20 | 1.28599 | 2.0012934 |
| Misc | LOC_Os01g40470.1 | Q8RU33 | F-type ATP synthase | H⁺-ATPase | -0.35 | -0.57 | -0.93 | -0.77 | 0.94 | 1.68 | 1.286626 | 2.1532804 |
| Misc | LOC_Os02g21520.1 | Q6EQW2 | Chalcone-flavonone isomerase family protein | CHI | -1.33 | -1.35 | 0.57 | 0.84 | 0.57 | 0.71 | 1.0617314 | 2.4037234 |
