# Supplementary material for: Comprehensive DIA‐MS Proteomics of Root Basal Nodes Elucidates Mechanisms of Salt Tolerance in Rice
Source: Proteomics. 2026 Apr 29;26(9):140–7. doi: 10.1002/pmic.70138 (PMC13519373; doi:10.1002/pmic.70138)
Supplement: Supplementary file 5 [file PMIC-26--s005.docx]

**Figure S1.** (A) Diagram of the data-independent acquisition (DIA) scheme, illustrating isolation windows across the m/z range of 400–1,200. (B) Multi-scatter plot depicting pairwise correlation among the six sample groups.

**Figure S2.** Integrated schematic pathway summarizing key molecular responses to salinity stress in rice root nodes based on DIA-MS proteomic analysis. The illustrated pathway highlights differentially modulated proteins in the salinity-tolerant CSA-Pi9 cultivar relative to the salinity-sensitive DJ cultivar. These proteins are mapped onto functional modules associated with salinity stress adaptation, including ion transport, osmotic regulation, membrane stabilization, energy metabolism, and stress signaling.
