# Supplementary material for: Comprehensive DIA‐MS Proteomics of Root Basal Nodes Elucidates Mechanisms of Salt Tolerance in Rice
Source: Proteomics. 2026 Apr 29;26(9):140–7. doi: 10.1002/pmic.70138 (PMC13519373; doi:10.1002/pmic.70138)
Supplement: Supplementary file 6 [file PMIC-26--s006.docx]

**Table S1.** Gene ontology (GO) enrichment analysis of differentially modulated proteins

| **Category** | **Term** | **Description** | **Count** | **P-Value** | **Fold Enrichment** | **Cluster** |
| --- | --- | --- | --- | --- | --- | --- |
| Biological process | GO:0009056 | catabolic process | 86 | 3.027E-07 | 1.750361339 | C_2 |
| Biological process | GO:0005975 | carbohydrate metabolic process | 71 | 9.14979E-11 | 2.298192679 | C_2 |
| Biological process | GO:0055085 | transmembrane transport (Transport) | 58 | 1.48204E-05 | 1.813170102 | C_2 |
| Biological process | GO:0006629 | lipid metabolic process | 54 | 0.000389987 | 1.640631912 | C_2 |
| Biological process | GO:0043436 | oxoacid metabolic process | 50 | 1.25533E-06 | 2.103788133 | C_2 |
| Biological process | GO:0006082 | organic acid metabolic process | 50 | 1.29777E-06 | 2.101307251 | C_2 |
| Biological process | GO:0019752 | carboxylic acid metabolic process | 48 | 4.10699E-06 | 2.05358008 | C_2 |
| Biological process | GO:0005976 | polysaccharide metabolic process | 31 | 0.000235628 | 2.076660339 | C_2 |
| Biological process | GO:0019637 | organophosphate metabolic process (phosphorus metabolic process) | 31 | 0.008420212 | 1.644022768 | C_2 |
| Biological process | GO:0008610 | lipid biosynthetic process | 30 | 0.003781212 | 1.761359356 | C_2 |
| Biological process | GO:1901135 | carbohydrate derivative metabolic process | 29 | 0.003490184 | 1.794282914 | C_2 |
| Biological process | GO:0046394 | carboxylic acid biosynthetic process | 27 | 0.000482839 | 2.119450697 | C_2 |
| Biological process | GO:0016053 | organic acid biosynthetic process | 27 | 0.000482839 | 2.119450697 | C_2 |
| Biological process | GO:0016052 | carbohydrate catabolic process | 26 | 2.96599E-05 | 2.581037452 | C_2 |
| Biological process | GO:0055086 | nucleobase-containing small molecule metabolic process (small molecule metabolic process) | 26 | 0.000436084 | 2.175099637 | C_2 |
| Biological process | GO:0032787 | monocarboxylic acid metabolic process | 25 | 0.000503254 | 2.194468656 | C_2 |
| Biological process | GO:1901700 | response to oxygen-containing compound (Response to chemical) | 24 | 0.014874 | 1.703816939 | C_2 |
| Biological process | GO:0044282 | small molecule catabolic process | 23 | 2.65033E-07 | 3.725808784 | C_2 |
| Biological process | GO:0016051 | carbohydrate biosynthetic process | 23 | 2.30732E-05 | 2.836255821 | C_2 |
| Biological process | GO:0006091 | generation of precursor metabolites and energy | 23 | 0.000321028 | 2.369011365 | C_2 |
| Biological process | GO:0006811 | monoatomic ion transport | 23 | 0.000968614 | 2.179994501 | C_2 |
| Biological process | GO:0006753 | nucleoside phosphate metabolic process | 23 | 0.002064796 | 2.054330658 | C_2 |
| Biological process | GO:0006520 | amino acid metabolic process | 23 | 0.008207344 | 1.825563324 | C_2 |
| Biological process | GO:0019748 | secondary metabolic process | 22 | 7.57479E-06 | 3.148754062 | C_2 |
| Biological process | GO:0000272 | polysaccharide catabolic process | 21 | 1.67712E-05 | 3.092568556 | C_2 |
| Biological process | GO:0006812 | monoatomic cation transport | 19 | 0.002862871 | 2.191343846 | C_2 |
| Biological process | GO:0090407 | organophosphate biosynthetic process | 19 | 0.03078091 | 1.709912244 | C_2 |
| Biological process | GO:0044248 | cellular catabolic process | 18 | 4.71624E-06 | 3.841239985 | C_2 |
| Biological process | GO:1901137 | carbohydrate derivative biosynthetic process | 18 | 0.028705792 | 1.757498843 | C_2 |
| Biological process | GO:0044550 | secondary metabolite biosynthetic process | 17 | 4.48821E-06 | 4.066100044 | C_2 |
| Biological process | GO:0009117 | nucleotide metabolic process | 17 | 0.005363507 | 2.179312614 | C_2 |
| Biological process | GO:0006720 | isoprenoid metabolic process | 17 | 0.006764056 | 2.125785637 | C_2 |
| Biological process | GO:0009698 | phenylpropanoid metabolic process | 16 | 0.000181203 | 3.150335556 | C_2 |
| Biological process | GO:0006631 | fatty acid metabolic process | 16 | 0.002164905 | 2.479177111 | C_2 |
| Biological process | GO:0034655 | nucleobase-containing compound catabolic process | 16 | 0.003248236 | 2.375878065 | C_2 |
| Biological process | GO:0046395 | carboxylic acid catabolic process | 15 | 7.26013E-05 | 3.611976788 | C_2 |
| Biological process | GO:0016054 | organic acid catabolic process | 15 | 7.26013E-05 | 3.611976788 | C_2 |
| Biological process | GO:0015980 | energy derivation by oxidation of organic compounds | 15 | 0.00011153 | 3.47125042 | C_2 |
| Biological process | GO:0098660 | inorganic ion transmembrane transport | 15 | 0.013033853 | 2.096362998 | C_2 |
| Biological process | GO:0170039 | proteinogenic amino acid metabolic process | 15 | 0.019752582 | 1.987258605 | C_2 |
| Biological process | GO:0170033 | L-amino acid metabolic process | 15 | 0.022778402 | 1.950994761 | C_2 |
| Biological process | GO:1901605 | alpha-amino acid metabolic process | 15 | 0.048011048 | 1.758462384 | C_2 |
| Biological process | GO:0072330 | monocarboxylic acid biosynthetic process | 14 | 0.00240281 | 2.668098362 | C_2 |
| Biological process | GO:0034220 | monoatomic ion transmembrane transport | 14 | 0.022997615 | 2.011832232 | C_2 |
| Biological process | GO:0009699 | phenylpropanoid biosynthetic process | 13 | 3.74974E-05 | 4.412344978 | C_2 |
| Biological process | GO:0008299 | isoprenoid biosynthetic process | 13 | 0.00530312 | 2.545583641 | C_2 |
| Biological process | GO:0009259 | ribonucleotide metabolic process | 13 | 0.007108022 | 2.451302765 | C_2 |
| Biological process | GO:0019693 | ribose phosphate metabolic process | 13 | 0.009736987 | 2.351757475 | C_2 |
| Biological process | GO:0098655 | monoatomic cation transmembrane transport | 13 | 0.017044938 | 2.175099637 | C_2 |
| Biological process | GO:0098662 | inorganic cation transmembrane transport | 13 | 0.028271817 | 2.02312761 | C_2 |
| Biological process | GO:0006721 | terpenoid metabolic process | 13 | 0.033519695 | 1.971473288 | C_2 |
| Biological process | GO:0005985 | sucrose metabolic process | 12 | 8.28769E-08 | 8.727715341 | C_2 |
| Biological process | GO:0005984 | disaccharide metabolic process | 12 | 1.94681E-05 | 5.152506647 | C_2 |
| Biological process | GO:0009311 | oligosaccharide metabolic process | 12 | 2.7361E-05 | 4.972768043 | C_2 |
| Biological process | GO:0006633 | fatty acid biosynthetic process | 11 | 0.008484097 | 2.66680191 | C_2 |
| Biological process | GO:0009063 | amino acid catabolic process | 10 | 0.001273504 | 3.791294784 | C_2 |
| Biological process | GO:1901136 | carbohydrate derivative catabolic process | 10 | 0.007371357 | 2.921161555 | C_2 |
| Biological process | GO:0046434 | organophosphate catabolic process | 10 | 0.008591909 | 2.851053678 | C_2 |
| Biological process | GO:0170035 | L-amino acid catabolic process | 9 | 0.000605263 | 4.716816747 | C_2 |
| Biological process | GO:0170040 | proteinogenic amino acid catabolic process | 9 | 0.001165152 | 4.276580517 | C_2 |
| Biological process | GO:1901606 | alpha-amino acid catabolic process | 9 | 0.002608058 | 3.773453397 | C_2 |
| Biological process | GO:0072523 | purine-containing compound catabolic process | 9 | 0.003474652 | 3.603859986 | C_2 |
| Biological process | GO:1901292 | nucleoside phosphate catabolic process | 9 | 0.005174149 | 3.376247776 | C_2 |
| Biological process | GO:0009134 | nucleoside diphosphate catabolic process | 8 | 0.008080914 | 3.476894729 | C_2 |
| Biological process | GO:0072526 | pyridine-containing compound catabolic process | 7 | 0.016406799 | 3.417358861 | C_2 |
| Biological process | GO:0015698 | inorganic anion transport | 7 | 0.017446325 | 3.371178335 | C_2 |
| Biological process | GO:0009414 | response to water deprivation | 7 | 0.042473967 | 2.741397767 | C_2 |
| Biological process | GO:0009415 | response to water | 7 | 0.048450264 | 2.653906349 | C_2 |
| Biological process | GO:0009065 | glutamine family amino acid catabolic process | 5 | 0.000845931 | 11.13692843 | C_2 |
| Biological process | GO:0046294 | formaldehyde catabolic process | 4 | 0.002261693 | 14.25526839 | C_2 |
| Biological process | GO:0043649 | dicarboxylic acid catabolic process | 4 | 0.003045528 | 12.9593349 | C_2 |
| Biological process | GO:0046185 | aldehyde catabolic process | 4 | 0.015149291 | 7.502772837 | C_2 |
| Biological process | GO:0015689 | molybdate ion transport | 3 | 0.007399434 | 21.38290258 | C_2 |
| Biological process | GO:0006538 | glutamate catabolic process | 3 | 0.024730775 | 11.87939032 | C_2 |
| Biological process | GO:0009059 | macromolecule biosynthetic process (mother term: biosynthetic process (GO:0009058) | 60 | 2.29717E-09 | 2.203578387 | C_3 |
| Biological process | GO:0010467 | gene expression | 52 | 1.40685E-08 | 2.284675075 | C_3 |
| Biological process | GO:0006412 | translation | 33 | 8.56111E-14 | 5.016476854 | C_3 |
| Biological process | GO:0034654 | nucleobase-containing compound biosynthetic process | 27 | 0.003779096 | 1.804159988 | C_3 |
| Biological process | GO:0044085 | cellular component biogenesis (cellular component organization or biogenesis) | 23 | 0.005097041 | 1.882166579 | C_3 |
| Biological process | GO:0006091 | generation of precursor metabolites and energy | 21 | 1.00087E-09 | 5.637275749 | C_3 |
| Biological process | GO:0033036 | macromolecule localization (localization) | 20 | 0.007354254 | 1.935017271 | C_3 |
| Biological process | GO:0051641 | cellular localization | 20 | 0.007918971 | 1.921009907 | C_3 |
| Biological process | GO:0032774 | RNA biosynthetic process | 20 | 0.042553327 | 1.602775307 | C_3 |
| Biological process | GO:0008104 | protein localization | 19 | 0.001796177 | 2.268297881 | C_3 |
| Biological process | GO:0070727 | cellular macromolecule localization | 19 | 0.001847263 | 2.262481732 | C_3 |
| Biological process | GO:0009057 | macromolecule catabolic process (catabolic process) | 18 | 0.033255341 | 1.712966109 | C_3 |
| Biological process | GO:0022613 | ribonucleoprotein complex biogenesis | 17 | 6.04432E-05 | 3.296396854 | C_3 |
| Biological process | GO:0006396 | RNA processing | 16 | 0.043609906 | 1.728015424 | C_3 |
| Biological process | GO:0042254 | ribosome biogenesis | 15 | 7.03089E-05 | 3.628157383 | C_3 |
| Biological process | GO:0009628 | response to abiotic stimulus (response to stimulus) | 14 | 0.014986222 | 2.121258738 | C_3 |
| Biological process | GO:0019684 | photosynthesis, light reaction | 13 | 6.95197E-11 | 14.72500948 | C_3 |
| Biological process | GO:0015979 | photosynthesis | 13 | 8.88888E-07 | 6.422610517 | C_3 |
| Biological process | GO:0015031 | protein transport (Transport) | 13 | 0.037050474 | 1.931921244 | C_3 |
| Biological process | GO:0016192 | vesicle-mediated transport | 12 | 0.029180993 | 2.087209641 | C_3 |
| Biological process | GO:0090407 | organophosphate biosynthetic process | 11 | 0.010501324 | 2.580023028 | C_3 |
| Biological process | GO:0009416 | response to light stimulus | 9 | 0.002607028 | 3.799670278 | C_3 |
| Biological process | GO:0044042 | glucan metabolic process (carbohydrate metabolic process) | 9 | 0.002754262 | 3.765439014 | C_3 |
| Biological process | GO:0009314 | response to radiation | 9 | 0.003507942 | 3.618733598 | C_3 |
| Biological process | GO:0006364 | rRNA processing | 9 | 0.007202084 | 3.202787207 | C_3 |
| Biological process | GO:0016072 | rRNA metabolic process | 9 | 0.0103538 | 3.006933313 | C_3 |
| Biological process | GO:0016052 | carbohydrate catabolic process | 9 | 0.039933434 | 2.32848875 | C_3 |
| Biological process | GO:0005982 | starch metabolic process | 8 | 1.29012E-06 | 14.28935831 | C_3 |
| Biological process | GO:0042274 | ribosomal small subunit biogenesis | 8 | 8.81413E-05 | 7.582108491 | C_3 |
| Biological process | GO:0000272 | polysaccharide catabolic process | 8 | 0.015641744 | 3.070440629 | C_3 |
| Biological process | GO:0016051 | carbohydrate biosynthetic process | 8 | 0.036369975 | 2.571095613 | C_3 |
| Biological process | GO:0009765 | photosynthesis, light harvesting | 7 | 1.60055E-07 | 27.0902418 | C_3 |
| Biological process | GO:0019252 | starch biosynthetic process | 7 | 8.22611E-07 | 20.97309042 | C_3 |
| Biological process | GO:0009250 | glucan biosynthetic process | 7 | 0.001937708 | 5.3732711 | C_3 |
| Biological process | GO:0000271 | polysaccharide biosynthetic process | 7 | 0.017038368 | 3.40400944 | C_3 |
| Biological process | GO:1901293 | nucleoside phosphate biosynthetic process | 7 | 0.02161942 | 3.21864259 | C_3 |
| Biological process | GO:0009251 | glucan catabolic process | 6 | 0.000656696 | 8.573614986 | C_3 |
| Biological process | GO:0005983 | starch catabolic process | 5 | 0.000114591 | 19.35017271 | C_3 |
| Biological process | GO:0009767 | photosynthetic electron transport chain | 5 | 0.000515037 | 13.26868986 | C_3 |
| Biological process | GO:0009651 | response to salt stress | 5 | 0.035210445 | 4.038296914 | C_3 |
| Biological process | GO:0009772 | photosynthetic electron transport in photosystem II | 4 | 0.000137337 | 37.15233161 | C_3 |
| Biological process | GO:0009059 | macromolecule biosynthetic process (mother term: biosynthetic process (GO:0009058) | 131 | 2.10222E-31 | 2.857080634 | C_4 |
| Biological process | GO:0010467 | gene expression | 128 | 2.02251E-37 | 3.339681246 | C_4 |
| Biological process | GO:0019538 | protein metabolic process | 99 | 0.022915263 | 1.206481526 | C_4 |
| Biological process | GO:0090304 | nucleic acid metabolic process | 86 | 1.45596E-12 | 2.199117007 | C_4 |
| Biological process | GO:0016070 | RNA metabolic process | 74 | 8.40014E-14 | 2.546233504 | C_4 |
| Biological process | GO:0034654 | nucleobase-containing compound biosynthetic process | 72 | 6.67217E-16 | 2.857049253 | C_4 |
| Biological process | GO:0032774 | RNA biosynthetic process | 69 | 1.88969E-18 | 3.283716732 | C_4 |
| Biological process | GO:0141187 | nucleic acid biosynthetic process | 69 | 6.53221E-18 | 3.206257534 | C_4 |
| Biological process | GO:0044085 | cellular component biogenesis | 64 | 8.18375E-16 | 3.110170112 | C_4 |
| Biological process | GO:0006396 | RNA processing | 58 | 7.53812E-18 | 3.71988551 | C_4 |
| Biological process | GO:0006412 | translation | 47 | 1.4054E-16 | 4.242840237 | C_4 |
| Biological process | GO:0022613 | ribonucleoprotein complex biogenesis | 42 | 8.77731E-17 | 4.836306408 | C_4 |
| Biological process | GO:0006996 | organelle organization | 38 | 0.00014301 | 1.938911264 | C_4 |
| Biological process | GO:0022607 | cellular component assembly | 36 | 1.54191E-08 | 2.985938693 | C_4 |
| Biological process | GO:0016071 | mRNA metabolic process | 34 | 4.4492E-10 | 3.592596522 | C_4 |
| Biological process | GO:0043933 | protein-containing complex organization | 34 | 9.15291E-10 | 3.492244664 | C_4 |
| Biological process | GO:0042254 | ribosome biogenesis | 31 | 1.75298E-11 | 4.452772436 | C_4 |
| Biological process | GO:0065003 | protein-containing complex assembly | 31 | 1.07902E-09 | 3.75794421 | C_4 |
| Biological process | GO:0006397 | mRNA processing | 27 | 1.24005E-08 | 3.808790085 | C_4 |
| Biological process | GO:0006325 | chromatin organization | 24 | 4.19761E-10 | 5.071900973 | C_4 |
| Biological process | GO:0000377 | RNA splicing, via transesterification reactions with bulged adenosine as nucleophile | 21 | 3.77226E-09 | 5.241155586 | C_4 |
| Biological process | GO:0000375 | RNA splicing, via transesterification reactions | 21 | 3.77226E-09 | 5.241155586 | C_4 |
| Biological process | GO:0006364 | rRNA processing | 21 | 6.28096E-08 | 4.437913351 | C_4 |
| Biological process | GO:0016072 | rRNA metabolic process | 21 | 1.76058E-07 | 4.16653016 | C_4 |
| Biological process | GO:0008380 | RNA splicing | 21 | 2.21734E-07 | 4.107430442 | C_4 |
| Biological process | GO:0009890 | negative regulation of biosynthetic process | 20 | 0.000514411 | 2.473404622 | C_4 |
| Biological process | GO:0009892 | negative regulation of metabolic process | 20 | 0.001528437 | 2.255906874 | C_4 |
| Biological process | GO:0010558 | negative regulation of macromolecule biosynthetic process | 19 | 0.000995996 | 2.409152962 | C_4 |
| Biological process | GO:0010605 | negative regulation of macromolecule metabolic process | 19 | 0.002522661 | 2.21560579 | C_4 |
| Biological process | GO:0000398 | mRNA splicing, via spliceosome | 18 | 1.4195E-07 | 4.964123077 | C_4 |
| Biological process | GO:0006338 | chromatin remodeling | 18 | 1.63853E-07 | 4.914973343 | C_4 |
| Biological process | GO:0010629 | negative regulation of gene expression | 17 | 0.000478099 | 2.765981393 | C_4 |
| Biological process | GO:0071826 | protein-RNA complex organization | 15 | 3.03236E-06 | 4.81019678 | C_4 |
| Biological process | GO:0022618 | protein-RNA complex assembly | 14 | 8.57258E-06 | 4.737404436 | C_4 |
| Biological process | GO:0034728 | nucleosome organization | 13 | 4.90196E-12 | 17.48878049 | C_4 |
| Biological process | GO:0071824 | protein-DNA complex organization | 13 | 5.96254E-08 | 8.148181818 | C_4 |
| Biological process | GO:0006334 | nucleosome assembly | 12 | 2.28064E-11 | 18.38564103 | C_4 |
| Biological process | GO:0065004 | protein-DNA complex assembly | 12 | 1.502E-07 | 8.485680473 | C_4 |
| Biological process | GO:0006351 | DNA-templated transcription | 12 | 0.024668365 | 2.148971029 | C_4 |
| Biological process | GO:0010608 | post-transcriptional regulation of gene expression | 11 | 0.013850422 | 2.476433281 | C_4 |
| Biological process | GO:0018193 | peptidyl-amino acid modification | 11 | 0.017382225 | 2.388685645 | C_4 |
| Biological process | GO:0002181 | cytoplasmic translation | 10 | 4.67755E-05 | 5.930851944 | C_4 |
| Biological process | GO:0080134 | regulation of response to stress | 10 | 0.009426486 | 2.814128728 | C_4 |
| Biological process | GO:0006457 | protein folding | 10 | 0.029923957 | 2.307821049 | C_4 |
| Biological process | GO:0007010 | cytoskeleton organization | 10 | 0.037371925 | 2.215137473 | C_4 |
| Biological process | GO:0006413 | translational initiation | 9 | 1.3373E-05 | 8.137906683 | C_4 |
| Biological process | GO:0072657 | protein localization to membrane (cellular localization) | 9 | 0.002771778 | 3.760699301 | C_4 |
| Biological process | GO:0000226 | microtubule cytoskeleton organization | 9 | 0.004905593 | 3.423533156 | C_4 |
| Biological process | GO:0051668 | localization within membrane | 9 | 0.007541793 | 3.182130178 | C_4 |
| Biological process | GO:0042273 | ribosomal large subunit biogenesis | 8 | 0.00157747 | 4.694206219 | C_4 |
| Biological process | GO:0090150 | establishment of protein localization to membrane | 8 | 0.002965725 | 4.202432234 | C_4 |
| Biological process | GO:0006417 | regulation of translation | 8 | 0.019399283 | 2.941702564 | C_4 |
| Biological process | GO:0051028 | mRNA transport | 7 | 0.000873882 | 6.227394541 | C_4 |
| Biological process | GO:0050657 | nucleic acid transport | 7 | 0.003686357 | 4.708517824 | C_4 |
| Biological process | GO:0051236 | establishment of RNA localization | 7 | 0.003686357 | 4.708517824 | C_4 |
| Biological process | GO:0050658 | RNA transport | 7 | 0.003686357 | 4.708517824 | C_4 |
| Biological process | GO:0006403 | RNA localization | 7 | 0.004402282 | 4.542334842 | C_4 |
| Biological process | GO:0015931 | nucleobase-containing compound transport | 7 | 0.027918987 | 3.040145366 | C_4 |
| Biological process | GO:0140694 | membraneless organelle assembly | 7 | 0.039493506 | 2.797814939 | C_4 |
| Biological process | GO:0006612 | protein targeting to membrane | 6 | 0.011517053 | 4.412553846 | C_4 |
| Biological process | GO:0006366 | transcription by RNA polymerase II | 6 | 0.032536207 | 3.376954474 | C_4 |
| Biological process | GO:0031047 | regulatory ncRNA-mediated gene silencing | 6 | 0.049410608 | 3.008559441 | C_4 |
| Biological process | GO:0006368 | transcription elongation by RNA polymerase II | 5 | 0.001741317 | 9.509814324 | C_4 |
| Biological process | GO:0006354 | DNA-templated transcription elongation | 5 | 0.004324045 | 7.453638254 | C_4 |
| Biological process | GO:0045047 | protein targeting to ER | 5 | 0.006829798 | 6.566300366 | C_4 |
| Biological process | GO:0072599 | establishment of protein localization to endoplasmic reticulum | 5 | 0.006829798 | 6.566300366 | C_4 |
| Biological process | GO:0018208 | peptidyl-proline modification | 5 | 0.013440644 | 5.407541478 | C_4 |
| Biological process | GO:1903313 | positive regulation of mRNA metabolic process | 5 | 0.023135256 | 4.596410256 | C_4 |
| Biological process | GO:0070972 | protein localization to endoplasmic reticulum | 5 | 0.024417296 | 4.521059269 | C_4 |
| Biological process | GO:0019538 | protein metabolic process | 141 | 0.001497655 | 1.254954396 | C_5 |
| Biological process | GO:0009059 | macromolecule biosynthetic process (mother term: biosynthetic process (GO:0009058) | 103 | 2.54857E-07 | 1.640634261 | C_5 |
| Biological process | GO:0010467 | gene expression | 97 | 1.96775E-09 | 1.848375198 | C_5 |
| Biological process | GO:0009056 | catabolic process | 57 | 0.027925826 | 1.311330283 | C_5 |
| Biological process | GO:0044085 | cellular component biogenesis | 51 | 5.51998E-05 | 1.810079691 | C_5 |
| Biological process | GO:0034654 | nucleobase-containing compound biosynthetic process | 48 | 0.018249129 | 1.39107267 | C_5 |
| Biological process | GO:0006996 | organelle organization | 46 | 0.000451679 | 1.714176428 | C_5 |
| Biological process | GO:0006412 | translation | 41 | 2.34128E-08 | 2.703124368 | C_5 |
| Biological process | GO:0032774 | RNA biosynthetic process | 40 | 0.032124166 | 1.390272513 | C_5 |
| Biological process | GO:0141187 | nucleic acid biosynthetic process | 40 | 0.043867513 | 1.357477542 | C_5 |
| Biological process | GO:0043933 | protein-containing complex organization | 32 | 1.21942E-05 | 2.400485427 | C_5 |
| Biological process | GO:0006396 | RNA processing | 32 | 0.023582006 | 1.498907761 | C_5 |
| Biological process | GO:0022607 | cellular component assembly | 30 | 0.002396073 | 1.817284785 | C_5 |
| Biological process | GO:0016071 | mRNA metabolic process | 26 | 0.001357627 | 2.006440226 | C_5 |
| Biological process | GO:0006979 | response to oxidative stress (response to stress) | 25 | 3.93085E-07 | 3.402292742 | C_5 |
| Biological process | GO:0022613 | ribonucleoprotein complex biogenesis | 22 | 0.008769698 | 1.850165373 | C_5 |
| Biological process | GO:0065003 | protein-containing complex assembly | 21 | 0.009705227 | 1.859222126 | C_5 |
| Biological process | GO:0072593 | reactive oxygen species metabolic process | 20 | 4.06511E-07 | 4.131604725 | C_5 |
| Biological process | GO:0042744 | hydrogen peroxide catabolic process | 19 | 5.15332E-07 | 4.275864666 | C_5 |
| Biological process | GO:0042743 | hydrogen peroxide metabolic process | 19 | 5.15332E-07 | 4.275864666 | C_5 |
| Biological process | GO:0006397 | mRNA processing | 18 | 0.018062547 | 1.854467082 | C_5 |
| Biological process | GO:0051604 | protein maturation | 17 | 0.015172914 | 1.939981539 | C_5 |
| Biological process | GO:0042254 | ribosome biogenesis | 17 | 0.030117741 | 1.783368446 | C_5 |
| Biological process | GO:0006457 | protein folding | 16 | 0.000947165 | 2.696779653 | C_5 |
| Biological process | GO:0008380 | RNA splicing | 15 | 0.010918592 | 2.142720536 | C_5 |
| Biological process | GO:0010608 | post-transcriptional regulation of gene expression | 13 | 0.019408154 | 2.137473057 | C_5 |
| Biological process | GO:0006325 | chromatin organization | 13 | 0.029834797 | 2.006440226 | C_5 |
| Biological process | GO:0071826 | protein-RNA complex organization | 11 | 0.010747047 | 2.576247714 | C_5 |
| Biological process | GO:0000398 | mRNA splicing, via spliceosome | 11 | 0.027395789 | 2.215573034 | C_5 |
| Biological process | GO:0000377 | RNA splicing, via transesterification reactions with bulged adenosine as nucleophile | 11 | 0.048952253 | 2.005043469 | C_5 |
| Biological process | GO:0000375 | RNA splicing, via transesterification reactions | 11 | 0.048952253 | 2.005043469 | C_5 |
| Biological process | GO:0034599 | cellular response to oxidative stress | 10 | 2.75953E-06 | 8.221050218 | C_5 |
| Biological process | GO:0062197 | cellular response to chemical stress | 10 | 1.17444E-05 | 6.945370012 | C_5 |
| Biological process | GO:0002181 | cytoplasmic translation | 10 | 0.00049475 | 4.331521083 | C_5 |
| Biological process | GO:0022618 | protein-RNA complex assembly | 10 | 0.020448098 | 2.471358654 | C_5 |
| Biological process | GO:0070925 | organelle assembly | 10 | 0.044782806 | 2.142720536 | C_5 |
| Biological process | GO:0006997 | nucleus organization | 9 | 2.67497E-07 | 12.94815409 | C_5 |
| Biological process | GO:0022411 | cellular component disassembly | 9 | 0.002810658 | 3.737611491 | C_5 |
| Biological process | GO:0009894 | regulation of catabolic process | 9 | 0.03689411 | 2.369596827 | C_5 |
| Biological process | GO:0009834 | plant-type secondary cell wall biogenesis | 8 | 0.000612369 | 5.462121501 | C_5 |
| Biological process | GO:0032984 | protein-containing complex disassembly | 8 | 0.001705177 | 4.603788122 | C_5 |
| Biological process | GO:0042274 | ribosomal small subunit biogenesis | 8 | 0.010936909 | 3.288420087 | C_5 |
| Biological process | GO:0006402 | mRNA catabolic process | 8 | 0.017903997 | 2.983936746 | C_5 |
| Biological process | GO:0009832 | plant-type cell wall biogenesis | 8 | 0.025369012 | 2.778148005 | C_5 |
| Biological process | GO:0006401 | RNA catabolic process | 8 | 0.040164434 | 2.517696629 | C_5 |
| Biological process | GO:0000302 | response to reactive oxygen species | 7 | 0.003924111 | 4.622656106 | C_5 |
| Biological process | GO:1903311 | regulation of mRNA metabolic process | 7 | 0.022023175 | 3.204341164 | C_5 |
| Biological process | GO:0009056 | catabolic process | 39 | 0.000626936 | 1.75887913 | C_6 |
| Biological process | GO:0005975 | carbohydrate metabolic process | 34 | 3.17206E-06 | 2.438648085 | C_6 |
| Biological process | GO:0043436 | oxoacid metabolic process | 27 | 2.58823E-05 | 2.517316884 | C_6 |
| Biological process | GO:0006082 | organic acid metabolic process | 27 | 2.64061E-05 | 2.51434835 | C_6 |
| Biological process | GO:0019752 | carboxylic acid metabolic process | 25 | 0.000142834 | 2.370022899 | C_6 |
| Biological process | GO:0044283 | small molecule biosynthetic process | 20 | 0.000306043 | 2.568102862 | C_6 |
| Biological process | GO:1901135 | carbohydrate derivative metabolic process | 18 | 0.001053138 | 2.467786344 | C_6 |
| Biological process | GO:0006520 | amino acid metabolic process | 17 | 0.000193007 | 2.989923766 | C_6 |
| Biological process | GO:0009628 | response to abiotic stimulus | 16 | 0.011159897 | 2.061185331 | C_6 |
| Biological process | GO:0019637 | organophosphate metabolic process (phosphorus metabolic process) | 16 | 0.023566771 | 1.880218167 | C_6 |
| Biological process | GO:0016053 | organic acid biosynthetic process | 15 | 0.001932111 | 2.609113315 | C_6 |
| Biological process | GO:0046394 | carboxylic acid biosynthetic process | 15 | 0.001932111 | 2.609113315 | C_6 |
| Biological process | GO:0005976 | polysaccharide metabolic process | 15 | 0.007757325 | 2.226574145 | C_6 |
| Biological process | GO:1901605 | alpha-amino acid metabolic process | 14 | 0.000133472 | 3.63673777 | C_6 |
| Biological process | GO:0055086 | nucleobase-containing small molecule metabolic process (nucleobase-containing compund metabolic process | 14 | 0.003032363 | 2.595230709 | C_6 |
| Biological process | GO:0006979 | response to oxidative stress | 13 | 0.000393195 | 3.468240267 | C_6 |
| Biological process | GO:0170039 | proteinogenic amino acid metabolic process | 12 | 0.000636398 | 3.52278794 | C_6 |
| Biological process | GO:0170033 | L-amino acid metabolic process | 12 | 0.000741363 | 3.458503489 | C_6 |
| Biological process | GO:0006753 | nucleoside phosphate metabolic process | 12 | 0.012760299 | 2.375012421 | C_6 |
| Biological process | GO:0042744 | hydrogen peroxide catabolic process | 11 | 9.37188E-05 | 4.852853592 | C_6 |
| Biological process | GO:0042743 | hydrogen peroxide metabolic process | 11 | 9.37188E-05 | 4.852853592 | C_6 |
| Biological process | GO:0072593 | reactive oxygen species metabolic process | 11 | 0.00018921 | 4.454670733 | C_6 |
| Biological process | GO:0016052 | carbohydrate catabolic process | 11 | 0.015987506 | 2.419667947 | C_6 |
| Biological process | GO:0019748 | secondary metabolic process | 10 | 0.004478041 | 3.171452329 | C_6 |
| Biological process | GO:0016051 | carbohydrate biosynthetic process | 10 | 0.011285725 | 2.732496989 | C_6 |
| Biological process | GO:0008652 | amino acid biosynthetic process | 9 | 0.005414117 | 3.368352924 | C_6 |
