# Supplementary material for: Comprehensive DIA‐MS Proteomics of Root Basal Nodes Elucidates Mechanisms of Salt Tolerance in Rice
Source: Proteomics. 2026 Apr 29;26(9):140–7. doi: 10.1002/pmic.70138 (PMC13519373; doi:10.1002/pmic.70138)
Supplement: Supplementary file 7 [file PMIC-26--s007.docx]

**Table S7.** RNA-seq analysis for validation of selected proteins with increased abundance in CSA-Pi9_S sample

| **Locus IDs** | **Gene description** | **Gene symbols** | | **DJ_C** | | **DJ_S** | **CSA_C** | **CSA_S** | **CSA-Pi9_C** | **CSA-Pi9_S** | **FC (CSA-Pi9_S vs CSA-Pi9_C)** | **FC (CSA-Pi9_S vs DJ_S)** |
| --- | --- | --- | --- | --- | --- | --- | --- | --- | --- | --- | --- | --- |
| LOC_Os09g21000 | Potassium transporter 23 (High-affinity potassium transporter) | | OsHAK23 | | 11.36 | 11.43 | 12.22 | 12.03 | 12.23 | 12.23 | 1.005542 | 1.740326 |
| LOC_Os04g54200 | Diacylglycerol kinase 1 | | DGK1 | | 10.31 | 9.974 | 12.17 | 12.03 | 12.05 | 11.98 | -1.04851 | 4.020982 |
| LOC_Os11g35710 | Cycloartenol synthase 1 | | CAS1 | | 10.6 | 10.43 | 10.29 | 10.57 | 13.59 | 13.62 | 1.022996 | 9.111355 |
| LOC_Os04g28620 | Fatty acyl-CoA reductase | | FAR1 | | 9.961 | 9.904 | 11.4 | 10.71 | 11.22 | 11.32 | 1.076712 | 2.67553 |
| LOC_Os08g44360 | Fatty acyl-CoA reductase | | FAR1 | | 13.38 | 13.16 | 13.82 | 13.74 | 13.98 | 14.08 | 1.07507 | 1.890549 |
| LOC_Os04g02640 | 3-ketoacyl-CoA synthase 6 | | KCS6 | | 9.118 | 6.714 | 11.6 | 10.9 | 11.7 | 11.58 | -1.08366 | 29.24147 |
| LOC_Os09g34930 | 3-ketoacyl-CoA synthase 4 | | KCS4 | | 9.664 | 9.403 | 9.846 | 9.812 | 9.837 | 10.17 | 1.26353 | 1.707488 |
| LOC_Os09g23550 | cinnamyl alcohol dehydrogenase 8C | | CAD8C | | 12.2 | 12.01 | 13.58 | 13.52 | 13.72 | 13.79 | 1.051113 | 3.421752 |
| LOC_Os09g23540 | Cinnamyl alcohol dehydrogenase 8B | | CAD8B | | 9.562 | 9.353 | 12.92 | 12.83 | 13.19 | 13.24 | 1.035828 | 14.7597 |
| LOC_Os06g24704 | Acyl-coenzyme A oxidase 3 | | ACX3 | | 10.67 | 10.67 | 11.26 | 11.22 | 11.51 | 11.47 | -1.02691 | 1.751424 |
| LOC_Os08g08840 | Glucose-6-phosphate/phosphate-translocator | | GPT2 | | 14.31 | 14.15 | 14.28 | 14.33 | 14.96 | 14.9 | -1.04123 | 1.684381 |
| LOC_Os10g33170 | NRT1 (nitrate transporter 1)/PTR (peptide transporter) family 5.2 | | PTR2 | | 11.03 | 10.67 | 11.29 | 11.11 | 11.44 | 11.54 | 1.074272 | 1.830477 |
| LOC_Os02g47510 | NCED1 (NINE-CIS-EPOXYCAROTENOID DIOXYGENASE 1) | | NCED1 | | 9.954 | 9.663 | 10.36 | 10.57 | 10.88 | 11.13 | 1.181678 | 2.755523 |
| LOC_Os01g14410 | ELIP1 (EARLY LIGHT-INDUCIBLE PROTEIN); chlorophyll binding | | ELIP1 | | 8.289 | 8.147 | 10.55 | 10.19 | 10.66 | 11.43 | 1.696656 | 9.709187 |
| LOC_Os03g22810 | CSD1 (COPPER/ZINC SUPEROXIDE DISMUTASE 1); superoxide dismutase | | CSD1 | | 13.93 | 13.6 | 14.58 | 14.39 | 14.83 | 14.68 | -1.10997 | 2.117548 |
| LOC_Os11g42500 | disease resistance-responsive family protein | | - | | 11.99 | 11.85 | 11.85 | 12.22 | 12.08 | 13.01 | 1.905369 | 2.23255 |
| LOC_Os03g51930 | unknown protein | | - | | 10.37 | 10.32 | 10.99 | 11.05 | 11.34 | 11.49 | 1.114233 | 2.250129 |
| LOC_Os10g06140 | WAK2; ATP binding / calcium ion binding / protein kinase/ protein serine/threonine kinase | | WAK2 | | 7.331 | 6.723 | 4.073 | 4.049 | 5.997 | 6.207 | 1.156799 | -1.43021 |
| LOC_Os11g20160 | O-methyltransferase family 2 protein | | - | | 10.83 | 10.82 | 11.23 | 11.44 | 12.16 | 12.22 | 1.039255 | 2.646094 |
| LOC_Os10g11140 | PGM (PHOSPHOGLUCOMUTASE); phosphoglucomutase | | PGM | | 13.26 | 13.1 | 14.31 | 14.5 | 14.55 | 14.61 | 1.04431 | 2.852962 |
| LOC_Os04g53780 | BAN (BANYULS); oxidoreductase | | BAN | | 8.545 | 8.183 | 9.32 | 9.167 | 9.751 | 9.668 | -1.05915 | 2.799845 |
| LOC_Os07g43390 | DPE1 (DISPROPORTIONATING ENZYME); 4-alpha-glucanotransferase/ catalytic/ cation binding | | DPE1 | | 12.91 | 12.89 | 13.14 | 13.34 | 13.56 | 13.77 | 1.156615 | 1.846225 |
| LOC_Os09g12660 | ADG1 (ADP GLUCOSE PYROPHOSPHORYLASE 1); glucose-1-phosphate adenylyltransferase | | ADG1 | | 14.8 | 14.42 | 15.59 | 15.71 | 16.22 | 16.37 | 1.113845 | 3.864001 |
| LOC_Os06g06560 | SSI1 (SUPPRESSOR OF SALICYLIC ACID INSENSITIVITY 1); starch synthase/ transferase, transferring glycosyl groups | | SSI1 | | 11.63 | 11.71 | 11.84 | 12.02 | 12.69 | 12.71 | 1.020669 | 2.004124 |
| LOC_Os05g32710 | isoamylase, putative / starch debranching enzyme, putative | | ATISA2 | | 11.65 | 11.57 | 11.18 | 11.22 | 12.31 | 12.31 | -1.00228 | 1.666063 |
| LOC_Os03g55090 | glucan phosphorylase, putative | | - | | 14.74 | 15.01 | 15.06 | 15.41 | 15.67 | 15.74 | 1.051189 | 1.668306 |
| LOC_Os01g63270 | PHS2 (ALPHA-GLUCAN PHOSPHORYLASE 2); phosphorylase/ transferase, transferring glycosyl groups | | ATPHS2 | | 13.76 | 13.88 | 14.31 | 14.47 | 14.75 | 14.77 | 1.009509 | 1.848707 |
| LOC_Os06g16640 | unknown protein | | - | | 10.81 | 11.59 | 11.83 | 12.51 | 11.75 | 12.53 | 1.719794 | 1.930169 |
| LOC_Os01g40550 | Unknown | | - | | 8.294 | 7.923 | 8.767 | 8.755 | 8.04 | 8.515 | 1.390075 | 1.507215 |
| LOC_Os11g03390 | forkhead-associated domain-containing protein / FHA domain-containing protein | | - | | 7.315 | 7.539 | 9.092 | 9.132 | 9.736 | 9.857 | 1.087737 | 4.98795 |
