# Supplementary material for: Comprehensive DIA‐MS Proteomics of Root Basal Nodes Elucidates Mechanisms of Salt Tolerance in Rice
Source: Proteomics. 2026 Apr 29;26(9):140–7. doi: 10.1002/pmic.70138 (PMC13519373; doi:10.1002/pmic.70138)
Supplement: Supplementary file 8 [file PMIC-26--s008.docx]

**Table S2.** MapMan analysis of the whole differentially modulated proteins in each sample

| **Locus IDs** | **Uniprot IDs** | **Functional annotation** | **TAIR10** | **Symbols** | **Cluster** | **Cat** |
| --- | --- | --- | --- | --- | --- | --- |
| LOC_Os09g17740.1 | P12330 | PS.lightreaction.photosystem II.LHC-II | AT2G34430 | LHB1B1 | C_3 | increased in CSA-Pi9 sample sets |
| LOC_Os07g37240.1 | Q6Z411 | PS.lightreaction.photosystem II.LHC-II | AT5G01530 | - | C_3 | increased in CSA-Pi9 sample sets |
| LOC_Os07g37550.1 | Q6ZF30 | PS.lightreaction.photosystem II.LHC-II | AT5G54270 | LHCB3 | C_3 | increased in CSA-Pi9 sample sets |
| LOC_Os08g33820.1 | Q6YWJ7 | PS.lightreaction.photosystem II.LHC-II | AT3G47470 | LHCA4 | C_2 | increased in CSA-Pi9 sample sets |
| LOC_Os01g52240.1 | Q8W0E6 | PS.lightreaction.photosystem II.LHC-II | AT2G34430 | LHB1B1 | C_3 | increased in CSA-Pi9 sample sets |
| LOC_Os11g13890.1 | Q53N83 | PS.lightreaction.photosystem II.LHC-II | AT4G10340 | LHCB5 | C_3 | increased in CSA-Pi9 sample sets |
| LOC_Os01g41710.1 | I3QD41 | PS.lightreaction.photosystem II.LHC-II | AT2G34430 | LHB1B1 | C_3 | increased in CSA-Pi9 sample sets |
| LOC_Os02g24632.1 | Q6Z1U5 | PS.lightreaction.photosystem II.PSII polypeptide subunits | ATCG00280 | PSBC | C_3 | increased in CSA-Pi9 sample sets |
| LOC_Os06g39708.1 | Q69VC8 | PS.lightreaction.photosystem II.PSII polypeptide subunits | ATCG00680 | PSBB | C_3 | increased in CSA-Pi9 sample sets |
| LOC_Os04g16770.1 | Q0JEM7 | PS.lightreaction.photosystem II.PSII polypeptide subunits | ATCG00020 | PSBA | C_3 | increased in CSA-Pi9 sample sets |
| LOC_Os02g24634.1 | Q6Z1U4 | PS.lightreaction.photosystem II.PSII polypeptide subunits | ATCG00270 | PSBD | C_2 | increased in CSA-Pi9 sample sets |
| LOC_Os06g39728.1 | Q7F2L7 | PS.lightreaction.photosystem II.PSII polypeptide subunits | ATCG00580 | PSBE | C_3 | increased in CSA-Pi9 sample sets |
| LOC_Os07g32880.1 | Q84NW1 | PS.lightreaction.ATP synthase.gamma chain | AT4G04640 | ATPC1 | C_3 | increased in CSA-Pi9 sample sets |
| LOC_Os10g21236.1 | Q8S6G0 | PS.lightreaction.ATP synthase.chloroplastic subunit a | ATCG00150 | ATPI | C_2 | increased in CSA-Pi9 sample sets |
| LOC_Os10g37180.1 | A3C6G9 | PS.photorespiration.glycine cleavage.H protein | AT1G32470 | - | C_4 | increased in CSA-Pi9 sample sets |
| LOC_Os01g62420.1 | Q8LR75 | PS.calvin cycle.TPI | AT3G55440 | ATCTIMC | C_2 | increased in CSA-Pi9 sample sets |
| LOC_Os01g05490.1 | P48494 | PS.calvin cycle.TPI | AT3G55440 | ATCTIMC | C_2 | increased in CSA-Pi9 sample sets |
| LOC_Os06g45370.1 | Q655Y9 | PS.calvin cycle.FBPase | AT5G64380 | - | C_2 | increased in CSA-Pi9 sample sets |
| LOC_Os11g12810.1 | Q53JI9 | major CHO metabolism.synthesis.sucrose.SPS | AT4G10120 | ATSPS4F | C_2 | increased in CSA-Pi9 sample sets |
| LOC_Os06g43630.1 | Q67WN8 | major CHO metabolism.synthesis.sucrose.SPS | AT5G20280 | ATSPS1F | C_2 | increased in CSA-Pi9 sample sets |
| LOC_Os01g27880.1 | Q94E75 | major CHO metabolism.synthesis.sucrose.SPP | AT2G35840 | - | C_2 | increased in CSA-Pi9 sample sets |
| LOC_Os08g25734.1 | P15280 | major CHO metabolism.synthesis.starch.AGPase | AT5G48300 | ADG1 | C_3 | increased in CSA-Pi9 sample sets |
| LOC_Os09g12660.1 | Q69T99 | major CHO metabolism.synthesis.starch.AGPase | AT5G48300 | ADG1 | C_3 | increased in CSA-Pi9 sample sets |
| LOC_Os03g52460.1 | Q6AVT2 | major CHO metabolism.synthesis.starch.AGPase | AT5G19220 | ADG2 | C_2 | increased in CSA-Pi9 sample sets |
| LOC_Os01g44220.1 | Q7G065 | major CHO metabolism.synthesis.starch.AGPase | AT1G27680 | APL2 | C_2 | increased in CSA-Pi9 sample sets |
| LOC_Os05g50380.1 | Q688T8 | major CHO metabolism.synthesis.starch.AGPase | AT1G27680 | APL2 | C_2 | increased in CSA-Pi9 sample sets |
| LOC_Os05g45720.1 | Q84VC7 | major CHO metabolism.synthesis.starch.starch synthase | AT4G18240 | ATSS4 | C_3 | increased in CSA-Pi9 sample sets |
| LOC_Os02g32660.1 | Q6H6P8 | major CHO metabolism.synthesis.starch.starch branching | AT5G03650 | SBE2.2 | C_3 | increased in CSA-Pi9 sample sets |
| LOC_Os08g40930.1 | D0TZF0 | major CHO metabolism.synthesis.starch.debranching | AT2G39930 | ISA1 | C_3 | increased in CSA-Pi9 sample sets |
| LOC_Os05g32710.1 | Q6AU80 | major CHO metabolism.synthesis.starch.debranching | AT1G03310 | ATISA2 | C_3 | increased in CSA-Pi9 sample sets |
| LOC_Os01g63220.1 | Q5N7A1 | major CHO metabolism.degradation.sucrose.fructokinase | AT3G54090 | - | C_4 | increased in CSA-Pi9 sample sets |
| LOC_Os04g24430.1 | H6TFZ4 | major CHO metabolism.degradation.sucrose.Susy | AT1G73370 | SUS6 | C_2 | increased in CSA-Pi9 sample sets |
| LOC_Os02g58480.1 | Q6K973 | major CHO metabolism.degradation.sucrose.Susy | AT1G73370 | SUS6 | C_2 | increased in CSA-Pi9 sample sets |
| LOC_Os06g09450.1 | I3QD82 | major CHO metabolism.degradation.sucrose.Susy | AT3G43190 | SUS4 | C_2 | increased in CSA-Pi9 sample sets |
| LOC_Os01g51754.4 | Q5JLT1 | major CHO metabolism.degradation.starch.starch cleavage.alpha amylase | AT1G69830 | ATAMY3 | C_2 | increased in CSA-Pi9 sample sets |
| LOC_Os07g35940.1 | Q6Z5B2 | major CHO metabolism.degradation.starch.starch cleavage.beta amylase | AT4G15210 | ATBETA-AMY | C_2 | increased in CSA-Pi9 sample sets |
| LOC_Os09g39570.1 | Q652P5 | major CHO metabolism.degradation.starch.starch cleavage.beta amylase | AT4G00490 | BAM2 | C_2 | increased in CSA-Pi9 sample sets |
| LOC_Os01g63270.1 | Q8LQ33 | major CHO metabolism.degradation.starch.starch phosphorylase | AT3G46970 | ATPHS2 | C_3 | increased in CSA-Pi9 sample sets |
| LOC_Os07g43390.1 | F2ZBU6 | major CHO metabolism.degradation.starch.D enzyme | AT5G64860 | DPE1 | C_3 | increased in CSA-Pi9 sample sets |
| LOC_Os09g29404.1 | B9G434 | major CHO metabolism.degradation.starch.ISA3 | AT4G09020 | ATISA3 | C_3 | increased in CSA-Pi9 sample sets |
| LOC_Os09g23350.1 | Q6ERD9 | minor CHO metabolism.trehalose.TPS | AT1G68020 | ATTPS6 | C_3 | increased in CSA-Pi9 sample sets |
| LOC_Os03g13390.2 | Q10PE7 | minor CHO metabolism.others | AT1G59960 | - | C_2 | increased in CSA-Pi9 sample sets |
| LOC_Os09g15820.1 | Q6K2P3 | minor CHO metabolism.others | AT5G66530 | - | C_2 | increased in CSA-Pi9 sample sets |
| LOC_Os01g48200.1 | Q0JKI5 | minor CHO metabolism.callose | AT4G03550 | ATGSL05 | C_2 | increased in CSA-Pi9 sample sets |
| LOC_Os01g33420.1 | Q5QLK3 | minor CHO metabolism.galactose.alpha-galactosidases | AT3G26380 | - | C_2 | increased in CSA-Pi9 sample sets |
| LOC_Os08g37380.1 | Q6ZB94 | glycolysis.plastid branch.glucose-6-phosphate isomerase | AT4G24620 | PGI1 | C_2 | increased in CSA-Pi9 sample sets |
| LOC_Os01g53680.1 | Q5JNC6 | glycolysis.plastid branch.phosphofructokinase (PFK) | AT4G26270 | PFK3 | C_2 | increased in CSA-Pi9 sample sets |
| LOC_Os03g46910.1 | Q84R73 | glycolysis.plastid branch.pyruvate kinase (PK) | AT3G22960 | PKP1 | C_2 | increased in CSA-Pi9 sample sets |
| LOC_Os01g06660.1 | Q9LGL5 | fermentation.PDC | AT4G33070 | - | C_2 | increased in CSA-Pi9 sample sets |
| LOC_Os02g10070.1 | Q7F8R1 | TCA / org. transformation.TCA.CS | AT2G44350 | ATCS | C_3 | increased in CSA-Pi9 sample sets |
| LOC_Os07g04240.1 | Q6ZDY8 | TCA / org. transformation.TCA.succinate dehydrogenase | AT5G66760 | SDH1-1 | C_2 | increased in CSA-Pi9 sample sets |
| LOC_Os11g47330.1 | Q2QZ86 | TCA / org. transformation.other organic acid transformaitons.atp-citrate lyase | AT1G09430 | ACLA-3 | C_2 | increased in CSA-Pi9 sample sets |
| LOC_Os03g56300.1 | Q7XZW1 | mitochondrial electron transport / ATP synthesis.NADH-DH.localisation not clear | AT1G79010 | - | C_3 | increased in CSA-Pi9 sample sets |
| LOC_Os07g45090.1 | Q8H2T7 | mitochondrial electron transport / ATP synthesis.NADH-DH.localisation not clear | AT5G08530 | CI51 | C_2 | increased in CSA-Pi9 sample sets |
| LOC_Os12g34094.1 | Q8HCP7 | mitochondrial electron transport / ATP synthesis.NADH-DH.localisation not clear | ATMG00580 | NAD4 | C_2 | increased in CSA-Pi9 sample sets |
| LOC_Os05g43360.1 | Q6L4W7 | mitochondrial electron transport / ATP synthesis.NADH-DH.localisation not clear | AT4G02580 | - | C_3 | increased in CSA-Pi9 sample sets |
| LOC_Os06g05080.1 | Q9SNQ3 | mitochondrial electron transport / ATP synthesis.cytochrome c oxidase | AT1G80230 | - | C_2 | increased in CSA-Pi9 sample sets |
| LOC_Os03g56190.1 | Q7XZW4 | mitochondrial electron transport / ATP synthesis.cytochrome c oxidase | AT4G37830 | - | C_2 | increased in CSA-Pi9 sample sets |
| LOC_Os04g16740.1 | Q7DNB0 | mitochondrial electron transport / ATP synthesis.F1-ATPase | ATCG00120 | ATPA | C_3 | increased in CSA-Pi9 sample sets |
| LOC_Os06g43850.1 | Q67W57 | mitochondrial electron transport / ATP synthesis.F1-ATPase | AT5G13450 | - | C_3 | increased in CSA-Pi9 sample sets |
| LOC_Os05g47980.1 | A0A067Y2M2 | mitochondrial electron transport / ATP synthesis.F1-ATPase | AT5G08690 | - | C_2 | increased in CSA-Pi9 sample sets |
| LOC_Os01g52200.1 | A2ZXB0 | mitochondrial electron transport / ATP synthesis.F1-ATPase | AT4G29480 | - | C_4 | increased in CSA-Pi9 sample sets |
| LOC_Os03g16980.1 | Q8W3J0 | cell wall.precursor synthesis.UXS | AT2G28760 | UXS6 | C_3 | increased in CSA-Pi9 sample sets |
| LOC_Os07g36630.1 | Q84S18 | cell wall.cellulose synthesis.cellulose synthase | AT3G03050 | CSLD3 | C_2 | increased in CSA-Pi9 sample sets |
| LOC_Os01g19220.1 | Q94IY5 | cell wall.degradation.cellulases and beta -1,4-glucanases | AT5G64570 | XYL4 | C_2 | increased in CSA-Pi9 sample sets |
| LOC_Os03g53860.1 | Q8W2Z2 | cell wall.degradation.cellulases and beta -1,4-glucanases | AT5G20950 | - | C_2 | increased in CSA-Pi9 sample sets |
| LOC_Os01g19220.1 | Q94IY5 | cell wall.degradation.cellulases and beta -1,4-glucanases | AT5G64570 | XYL4 | C_2 | increased in CSA-Pi9 sample sets |
| LOC_Os01g54300.1 | Q0JJD4 | cell wall.degradation.mannan-xylose-arabinose-fucose | AT2G20680 | - | C_2 | increased in CSA-Pi9 sample sets |
| LOC_Os02g57770.1 | Q6K9X1 | cell wall.modification | AT2G36870 | - | C_2 | increased in CSA-Pi9 sample sets |
| LOC_Os01g57854.1 | Q5ZAW1 | cell wall.pectin*esterases.PME | AT1G11580 | ATPMEPCRA | C_2 | increased in CSA-Pi9 sample sets |
| LOC_Os06g09340.1 | Q69Y54 | cell wall.pectin*esterases.PME | AT2G26440 | - | C_2 | increased in CSA-Pi9 sample sets |
| LOC_Os08g34900.1 | Q6ZLF6 | cell wall.pectin*esterases.PME | AT3G43270 | - | C_2 | increased in CSA-Pi9 sample sets |
| LOC_Os04g57850.1 | Q7XQ93 | lipid metabolism.FA synthesis and FA elongation.acyl coa ligase | AT5G16370 | AAE5 | C_2 | increased in CSA-Pi9 sample sets |
| LOC_Os05g49290.1 | Q688V9 | lipid metabolism.FA synthesis and FA elongation.beta ketoacyl CoA synthase | AT1G19440 | KCS4 | C_2 | increased in CSA-Pi9 sample sets |
| LOC_Os06g39750.1 | Q69X62 | lipid metabolism.FA synthesis and FA elongation.beta ketoacyl CoA synthase | AT2G26640 | KCS11 | C_2 | increased in CSA-Pi9 sample sets |
| LOC_Os04g02640.1 | B7F5B4 | lipid metabolism.FA synthesis and FA elongation.beta ketoacyl CoA synthase | AT1G68530 | CUT1 | C_2 | increased in CSA-Pi9 sample sets |
| LOC_Os09g19650.1 | Q6K558 | lipid metabolism.FA synthesis and FA elongation.beta ketoacyl CoA synthase | AT4G34510 | KCS17 | C_2 | increased in CSA-Pi9 sample sets |
| LOC_Os09g34930.1 | Q650T5 | lipid metabolism.FA synthesis and FA elongation.beta ketoacyl CoA synthase | AT1G19440 | KCS4 | C_2 | increased in CSA-Pi9 sample sets |
| LOC_Os03g12030.1 | Q10PV5 | lipid metabolism.FA synthesis and FA elongation.beta ketoacyl CoA synthase | AT1G68530 | CUT1 | C_2 | increased in CSA-Pi9 sample sets |
| LOC_Os03g17520.1 | Q8H8U0 | lipid metabolism.Phospholipid synthesis | AT2G39290 | PGPS1 | C_2 | increased in CSA-Pi9 sample sets |
| LOC_Os11g41900.1 | Q2R0P3 | lipid metabolism.Phospholipid synthesis | AT3G57650 | LPAT2 | C_2 | increased in CSA-Pi9 sample sets |
| LOC_Os04g54200.1 | Q7XQT2 | lipid metabolism.Phospholipid synthesis.diacylglycerol kinase | AT2G20900 | DGK5 | C_2 | increased in CSA-Pi9 sample sets |
| LOC_Os01g70380.1 | Q5JK39 | lipid metabolism.''exotics'' (steroids, squalene etc).sphingolipids.serine C-palmitoyltransferase | AT5G23670 | LCB2 | C_2 | increased in CSA-Pi9 sample sets |
| LOC_Os05g49840.1 | Q6F357 | lipid metabolism.lipid degradation.lipases.triacylglycerol lipase | AT4G18550 | - | C_2 | increased in CSA-Pi9 sample sets |
| LOC_Os03g40670.1 | Q6AUZ6 | lipid metabolism.lipid degradation.lysophospholipases.glycerophosphodiester phosphodiesterase | AT5G08030 | - | C_2 | increased in CSA-Pi9 sample sets |
| LOC_Os01g55780.1 | Q8LR39 | lipid metabolism.lipid degradation.lysophospholipases.glycerophosphodiester phosphodiesterase | AT5G08030 | - | C_2 | increased in CSA-Pi9 sample sets |
| LOC_Os11g38050.1 | A0A0P0Y3X8 | lipid metabolism.lipid degradation.lysophospholipases.phosphoinositide phospholipase C | AT3G03530 | NPC4 | C_2 | increased in CSA-Pi9 sample sets |
| LOC_Os11g39220.1 | Q2R1G8 | lipid metabolism.lipid degradation.beta-oxidation.acyl CoA DH | AT5G65110 | ACX2 | C_2 | increased in CSA-Pi9 sample sets |
| LOC_Os06g24704.1 | Q69XR7 | lipid metabolism.lipid degradation.beta-oxidation.acyl CoA DH | AT1G06290 | ACX3 | C_2 | increased in CSA-Pi9 sample sets |
| LOC_Os08g44360.1 | Q6ZJ06 | lipid metabolism.lipid degradation.beta-oxidation.acyl CoA reductase | AT5G22500 | FAR1 | C_2 | increased in CSA-Pi9 sample sets |
| LOC_Os04g28620.1 | Q7XRZ6 | lipid metabolism.lipid degradation.beta-oxidation.acyl CoA reductase | AT5G22500 | FAR1 | C_2 | increased in CSA-Pi9 sample sets |
| LOC_Os08g14580.1 | Q7XXR4 | N-metabolism.ammonia metabolism.unspecified | AT4G25840 | GPP1 | C_2 | increased in CSA-Pi9 sample sets |
| LOC_Os08g36320.2 | Q9AQU4 | amino acid metabolism.synthesis.central amino acid metabolism.GABA.Glutamate decarboxylase | AT5G17330 | GAD | C_2 | increased in CSA-Pi9 sample sets |
| LOC_Os03g51080.1 | Q6ASV4 | amino acid metabolism.synthesis.central amino acid metabolism.GABA.Glutamate decarboxylase | AT5G17330 | GAD | C_2 | increased in CSA-Pi9 sample sets |
| LOC_Os04g37500.1 | Q7XV18 | amino acid metabolism.synthesis.central amino acid metabolism.GABA.Glutamate decarboxylase | AT2G02010 | GAD4 | C_2 | increased in CSA-Pi9 sample sets |
| LOC_Os03g18130.1 | Q10MX3 | amino acid metabolism.synthesis.aspartate family.asparagine | AT3G47340 | ASN1 | C_2 | increased in CSA-Pi9 sample sets |
| LOC_Os12g42876.1 | Q2QLY5 | amino acid metabolism.synthesis.aspartate family.methionine | AT5G17920 | ATCIMS | C_2 | increased in CSA-Pi9 sample sets |
| LOC_Os04g31960.1 | Q7XKQ8 | amino acid metabolism.synthesis.branched chain group.common | AT3G48560 | CSR1 | C_2 | increased in CSA-Pi9 sample sets |
| LOC_Os11g14950.1 | Q53L28 | amino acid metabolism.synthesis.branched chain group.common.acetolactate synthase | AT2G31810 | - | C_2 | increased in CSA-Pi9 sample sets |
| LOC_Os03g01600.1 | Q8H7T7 | amino acid metabolism.synthesis.branched chain group.common.branched-chain amino acid aminotransferase | AT5G65780 | ATBCAT-5 | C_2 | increased in CSA-Pi9 sample sets |
| LOC_Os04g08350.2 | Q7XS58 | amino acid metabolism.synthesis.serine-glycine-cysteine group.cysteine.OASTL | AT3G61440 | ATCYSC1 | C_2 | increased in CSA-Pi9 sample sets |
| LOC_Os03g44150.1 | Q10G56 | amino acid metabolism.degradation.glutamate family.arginine | AT5G46180 | DELTA-OAT | C_2 | increased in CSA-Pi9 sample sets |
| LOC_Os04g01590.1 | Q7X7N2 | amino acid metabolism.degradation.glutamate family.arginine | AT4G08900 | - | C_2 | increased in CSA-Pi9 sample sets |
| LOC_Os04g46370.1 | Q0JB91 | amino acid metabolism.degradation.aspartate family.asparagine.L-asparaginase | AT5G61540 | - | C_2 | increased in CSA-Pi9 sample sets |
| LOC_Os01g49330.1 | Q5N7L5 | amino acid metabolism.degradation.aspartate family.methionine | AT1G66520 | pde194 | C_4 | increased in CSA-Pi9 sample sets |
| LOC_Os02g19970.1 | Q6K5U4 | amino acid metabolism.degradation.aromatic aa.tyrosine | AT5G36160 | - | C_2 | increased in CSA-Pi9 sample sets |
| LOC_Os12g01530.1 | Q8L5K0 | metal handling.binding, chelation and storage | AT2G40300 | ATFER4 | C_2 | increased in CSA-Pi9 sample sets |
| LOC_Os05g33840.1 | O22567 | secondary metabolism.isoprenoids.non-mevalonate pathway.DXS | AT4G15560 | CLA1 | C_2 | increased in CSA-Pi9 sample sets |
| LOC_Os02g39160.1 | Q6K8J4 | secondary metabolism.isoprenoids.non-mevalonate pathway.HDS | AT5G60600 | GCPE | C_2 | increased in CSA-Pi9 sample sets |
| LOC_Os02g44780.1 | Q6ET88 | secondary metabolism.isoprenoids.non-mevalonate pathway.geranylgeranyl pyrophosphate synthase | AT4G38460 | GGR | C_2 | increased in CSA-Pi9 sample sets |
| LOC_Os01g02020.1 | Q9ASK5 | secondary metabolism.isoprenoids.mevalonate pathway.acetyl-CoA C-acyltransferase | AT5G47720 | - | C_2 | increased in CSA-Pi9 sample sets |
| LOC_Os07g36190.1 | Q8GVZ0 | secondary metabolism.isoprenoids.mevalonate pathway.isopentenyl pyrophosphate:dimethyllallyl pyrophosphate isomerase | AT3G02780 | IPP2 | C_3 | increased in CSA-Pi9 sample sets |
| LOC_Os05g34180.1 | Q6AUB0 | secondary metabolism.isoprenoids.mevalonate pathway.isopentenyl pyrophosphate:dimethyllallyl pyrophosphate isomerase | AT3G02780 | IPP2 | C_3 | increased in CSA-Pi9 sample sets |
| LOC_Os05g46580.1 | Q65XM9 | secondary metabolism.isoprenoids.mevalonate pathway.farnesyl pyrophosphate synthetase | AT5G47770 | FPS1 | C_3 | increased in CSA-Pi9 sample sets |
| LOC_Os02g19970.1 | Q6K5U4 | secondary metabolism.isoprenoids.tocopherol biosynthesis | AT5G36160 | - | C_2 | increased in CSA-Pi9 sample sets |
| LOC_Os02g09750.1 | Q6YUU2 | secondary metabolism.isoprenoids.carotenoids.lycopene beta cyclase | AT3G10230 | LYC | C_2 | increased in CSA-Pi9 sample sets |
| LOC_Os04g27340.1 | A3ASJ4 | secondary metabolism.isoprenoids.terpenoids | AT3G14520 | - | C_2 | increased in CSA-Pi9 sample sets |
| LOC_Os04g01810.1 | Q7X8W2 | secondary metabolism.isoprenoids.terpenoids | AT5G44630 | - | C_2 | increased in CSA-Pi9 sample sets |
| LOC_Os11g35710.1 | Q0IS49 | secondary metabolism.isoprenoids.terpenoids | AT2G07050 | CAS1 | C_2 | increased in CSA-Pi9 sample sets |
| LOC_Os12g16290.1 | Q2QUH0 | secondary metabolism.phenylpropanoids | AT1G32100 | ATPRR1 | C_2 | increased in CSA-Pi9 sample sets |
| LOC_Os01g67530.1 | A0A0P0VBX1 | secondary metabolism.phenylpropanoids | AT5G63380 | - | C_2 | increased in CSA-Pi9 sample sets |
| LOC_Os02g41650.1 | Q6K6Q1 | secondary metabolism.phenylpropanoids.lignin biosynthesis.PAL | AT2G37040 | pal1 | C_2 | increased in CSA-Pi9 sample sets |
| LOC_Os09g23540.1 | Q6ERW9 | secondary metabolism.phenylpropanoids.lignin biosynthesis.CAD | AT4G37980 | ELI3-1 | C_2 | increased in CSA-Pi9 sample sets |
| LOC_Os09g23550.1 | Q6ERW7 | secondary metabolism.phenylpropanoids.lignin biosynthesis.CAD | AT4G37970 | ATCAD6 | C_2 | increased in CSA-Pi9 sample sets |
| LOC_Os09g23560.1 | Q6ERW5 | secondary metabolism.phenylpropanoids.lignin biosynthesis.CAD | AT4G37980 | ELI3-1 | C_2 | increased in CSA-Pi9 sample sets |
| LOC_Os03g12270.1 | Q10PS6 | secondary metabolism.phenylpropanoids.lignin biosynthesis.CAD | AT4G39330 | ATCAD9 | C_2 | increased in CSA-Pi9 sample sets |
| LOC_Os10g29470.1 | Q337Y2 | secondary metabolism.phenylpropanoids.lignin biosynthesis.CAD | AT4G39330 | ATCAD9 | C_2 | increased in CSA-Pi9 sample sets |
| LOC_Os02g56920.1 | Q6K3D8 | secondary metabolism.wax | AT1G02205 | CER1 | C_2 | increased in CSA-Pi9 sample sets |
| LOC_Os02g21520.1 | Q6EQW2 | secondary metabolism.flavonoids.chalcones | AT1G53520 | - | C_2 | increased in CSA-Pi9 sample sets |
| LOC_Os03g13390.2 | Q10PE7 | secondary metabolism.flavonoids.chalcones | AT1G59960 | - | C_2 | increased in CSA-Pi9 sample sets |
| LOC_Os12g16290.1 | Q2QUH0 | secondary metabolism.phenylpropanoids | AT1G32100 | ATPRR1 | C_2 | increased in CSA-Pi9 sample sets |
| LOC_Os06g27770.1 | Q69XS7 | secondary metabolism.flavonoids.isoflavones.isoflavone reductase | AT4G39230 | - | C_2 | increased in CSA-Pi9 sample sets |
| LOC_Os01g63200.1 | Q0JHP8 | secondary metabolism.simple phenols | AT3G09220 | LAC7 | C_2 | increased in CSA-Pi9 sample sets |
| LOC_Os03g57680.1 | Q852M2 | hormone metabolism.abscisic acid.synthesis-degradation | AT5G20960 | AAO1 | C_2 | increased in CSA-Pi9 sample sets |
| LOC_Os01g38580.1 | Q93VD5 | hormone metabolism.abscisic acid.synthesis-degradation | AT4G32810 | CCD8 | C_2 | increased in CSA-Pi9 sample sets |
| LOC_Os12g44310.2 | Q2QLJ0 | hormone metabolism.abscisic acid.synthesis-degradation.synthesis.9-cis-epoxycarotenoid dioxygenase | AT3G63520 | CCD1 | C_2 | increased in CSA-Pi9 sample sets |
| LOC_Os03g49350.1 | Q7G794 | hormone metabolism.jasmonate.synthesis-degradation.lipoxygenase | AT3G22400 | LOX5 | C_2 | increased in CSA-Pi9 sample sets |
| LOC_Os04g56620.1 | Q7XM29 | Co-factor and vitamine metabolism | AT5G20990 | B73 | C_2 | increased in CSA-Pi9 sample sets |
| LOC_Os06g21980.1 | Q69T90 | Co-factor and vitamine metabolism.pantothenate.pantothenate kinase (PANK) | AT4G35360 | - | C_3 | increased in CSA-Pi9 sample sets |
| LOC_Os06g09910.1 | Q69K55 | Co-factor and vitamine metabolism.pantothenate.phosphopantothenoylcysteine decarboxylase (PPCDC) | AT3G18030 | ATHAL3A | C_3 | increased in CSA-Pi9 sample sets |
| LOC_Os03g44150.1 | Q10G56 | tetrapyrrole synthesis.GSA | AT5G46180 | DELTA-OAT | C_2 | increased in CSA-Pi9 sample sets |
| LOC_Os06g51060.1 | Q42993 | stress.biotic | AT3G12500 | ATHCHIB | C_2 | increased in CSA-Pi9 sample sets |
| LOC_Os05g33140.1 | Q688M5 | stress.biotic | AT3G12500 | ATHCHIB | C_2 | increased in CSA-Pi9 sample sets |
| LOC_Os04g41680.1 | Q7Y1Z0 | stress.biotic | AT3G54420 | ATEP3 | C_2 | increased in CSA-Pi9 sample sets |
| LOC_Os04g32550.1 | Q0JDK9 | stress.biotic | AT1G32210 | ATDAD1 | C_2 | increased in CSA-Pi9 sample sets |
| LOC_Os09g21210.1 | Q6H5B5 | stress.biotic | AT5G15870 | - | C_2 | increased in CSA-Pi9 sample sets |
| LOC_Os07g42520.1 | Q8GS22 | stress.biotic | - | - | C_2 | increased in CSA-Pi9 sample sets |
| LOC_Os06g25010.1 | B7F946 | stress.biotic | AT5G24090 | - | C_2 | increased in CSA-Pi9 sample sets |
| LOC_Os11g42550.1 | Q2R0H3 | stress.biotic.PR-proteins | AT1G65870 | - | C_2 | increased in CSA-Pi9 sample sets |
| LOC_Os11g07830.1 | Q53NQ9 | stress.biotic.PR-proteins | AT1G65870 | - | C_2 | increased in CSA-Pi9 sample sets |
| LOC_Os11g42500.1 | Q2R0I1 | stress.biotic.PR-proteins | AT5G42510 | - | C_2 | increased in CSA-Pi9 sample sets |
| LOC_Os02g36570.1 | Q69S79 | stress.abiotic | AT5G64940 | ATATH13 | C_2 | increased in CSA-Pi9 sample sets |
| LOC_Os12g26290.1 | Q2QRV3 | stress.abiotic | AT3G01420 | ALPHA-DOX1 | C_2 | increased in CSA-Pi9 sample sets |
| LOC_Os05g26902.1 | P0DO03 | stress.abiotic.heat | AT2G22360 | - | C_4 | increased in CSA-Pi9 sample sets |
| LOC_Os05g06440.1 | B9FHF3 | stress.abiotic.heat | AT3G62600 | ATERDJ3B | C_4 | increased in CSA-Pi9 sample sets |
| LOC_Os06g09910.1 | Q69K55 | stress.abiotic.drought/salt | AT3G18030 | ATHAL3A | C_3 | increased in CSA-Pi9 sample sets |
| LOC_Os04g48140.1 | Q7XRW8 | stress.abiotic.drought/salt | AT4G00740 | - | C_2 | increased in CSA-Pi9 sample sets |
| LOC_Os05g06430.1 | A0A067Y3P4 | redox.thioredoxin | AT2G47470 | ATPDIL2-1 | C_4 | increased in CSA-Pi9 sample sets |
| LOC_Os10g28200.1 | A3C4S4 | redox.ascorbate and glutathione.ascorbate.GME | AT5G28840 | GME | C_2 | increased in CSA-Pi9 sample sets |
| LOC_Os11g37890.1 | Q2R1V8 | redox.ascorbate and glutathione.ascorbate.GME | AT5G28840 | GME | C_2 | increased in CSA-Pi9 sample sets |
| LOC_Os11g04740.1 | Q2RAP0 | redox.ascorbate and glutathione.ascorbate.L-Galactono-1,4-lactone dehydrogenase | AT3G47930 | ATGLDH | C_2 | increased in CSA-Pi9 sample sets |
| LOC_Os12g04520.1 | Q2QXY1 | redox.ascorbate and glutathione.ascorbate.L-Galactono-1,4-lactone dehydrogenase | AT3G47930 | ATGLDH | C_2 | increased in CSA-Pi9 sample sets |
| LOC_Os08g15030.1 | Q9LD61 | nucleotide metabolism.synthesis.pyrimidine.aspartate transcarbamoylase | AT3G20330 | - | C_2 | increased in CSA-Pi9 sample sets |
| LOC_Os01g54370.1 | Q0JJD1 | nucleotide metabolism.synthesis.pyrimidine.dihydroorotase | AT4G22930 | PYR4 | C_2 | increased in CSA-Pi9 sample sets |
| LOC_Os01g72240.1 | Q9LDN2 | nucleotide metabolism.synthesis.pyrimidine.orotate phosphoribosyltransferase | AT3G54470 | - | C_2 | increased in CSA-Pi9 sample sets |
| LOC_Os11g25330.1 | Q53KM8 | nucleotide metabolism.degradation | AT5G18280 | ATAPY2 | C_2 | increased in CSA-Pi9 sample sets |
| LOC_Os11g25260.1 | Q53KM3 | nucleotide metabolism.degradation | AT5G18280 | ATAPY2 | C_2 | increased in CSA-Pi9 sample sets |
| LOC_Os05g28180.1 | Q84NP7 | nucleotide metabolism.degradation | AT2G38280 | FAC1 | C_2 | increased in CSA-Pi9 sample sets |
| LOC_Os11g03290.1 | B9GBL8 | nucleotide metabolism.degradation | AT5G18280 | ATAPY2 | C_2 | increased in CSA-Pi9 sample sets |
| LOC_Os09g39440.1 | Q652Q8 | nucleotide metabolism.degradation.pyrimidine.uridine nucleosidase | AT2G36310 | URH1 | C_2 | increased in CSA-Pi9 sample sets |
| LOC_Os03g31170.1 | Q84MH2 | nucleotide metabolism.degradation.pyrimidine.uridine nucleosidase | AT1G05620 | URH2 | C_2 | increased in CSA-Pi9 sample sets |
| LOC_Os07g30170.1 | Q8H530 | nucleotide metabolism.degradation.pyrimidine.beta-ureidopropionase | AT5G64370 | BETA-UP | C_2 | increased in CSA-Pi9 sample sets |
| LOC_Os12g40130.1 | Q2QMT1 | nucleotide metabolism.salvage.phosphoribosyltransferases.aprt | AT1G27450 | APT1 | C_2 | increased in CSA-Pi9 sample sets |
| LOC_Os02g40010.1 | Q6K5Y7 | nucleotide metabolism.salvage.phosphoribosyltransferases.aprt | AT1G80050 | APT2 | C_4 | increased in CSA-Pi9 sample sets |
| LOC_Os12g39860.1 | Q2QMV8 | nucleotide metabolism.salvage.phosphoribosyltransferases.aprt | AT1G27450 | APT1 | C_2 | increased in CSA-Pi9 sample sets |
| LOC_Os06g04910.1 | Q5VSC2 | nucleotide metabolism.salvage.NUDIX hydrolases | AT5G20070 | ATNUDT19 | C_2 | increased in CSA-Pi9 sample sets |
| LOC_Os04g01530.1 | B9FD10 | nucleotide metabolism.phosphotransfer and pyrophosphatases.uridylate kinase | AT4G25280 | - | C_4 | increased in CSA-Pi9 sample sets |
| LOC_Os02g01250.1 | Q6YU78 | RNA.processing | AT1G20580 | - | C_4 | increased in CSA-Pi9 sample sets |
| LOC_Os10g31900.1 | Q7XDP6 | RNA.processing | AT2G18510 | emb2444 | C_4 | increased in CSA-Pi9 sample sets |
| LOC_Os11g41820.1 | Q2R0Q1 | RNA.processing.splicing | AT1G60900 | - | C_4 | increased in CSA-Pi9 sample sets |
| LOC_Os10g02630.1 | Q33BE1 | RNA.processing.splicing | AT3G50670 | U1-70K | C_4 | increased in CSA-Pi9 sample sets |
| LOC_Os08g32100.1 | Q6ZA70 | RNA.processing.splicing | AT1G60200 | - | C_4 | increased in CSA-Pi9 sample sets |
| LOC_Os07g47630.1 | Q0D3Q1 | RNA.processing.splicing | AT1G09140 | - | C_4 | increased in CSA-Pi9 sample sets |
| LOC_Os02g58090.1 | Q6K7Q7 | RNA.processing.splicing | AT4G21660 | - | C_4 | increased in CSA-Pi9 sample sets |
| LOC_Os03g18720.1 | Q10MR0 | RNA.processing.splicing | AT2G30260 | U2B'' | C_4 | increased in CSA-Pi9 sample sets |
| LOC_Os05g48960.1 | Q6AUG0 | RNA.processing.splicing | AT5G42820 | ATU2AF35B | C_4 | increased in CSA-Pi9 sample sets |
| LOC_Os03g22380.1 | Q10LL5 | RNA.processing.splicing | AT3G49430 | SRp34a | C_3 | increased in CSA-Pi9 sample sets |
| LOC_Os02g03040.1 | Q6Z725 | RNA.processing.splicing | AT5G52040 | - | C_4 | increased in CSA-Pi9 sample sets |
| LOC_Os02g05310.1 | A3A338 | RNA.processing.splicing | AT5G64270 | - | C_4 | increased in CSA-Pi9 sample sets |
| LOC_Os02g39720.2 | B9F110 | RNA.processing.splicing | AT1G23860 | SRZ-21 | C_3 | increased in CSA-Pi9 sample sets |
| LOC_Os03g50090.1 | Q53RK8 | RNA.processing.RNA helicase | AT2G33730 | - | C_4 | increased in CSA-Pi9 sample sets |
| LOC_Os03g61220.1 | Q0DM51 | RNA.processing.RNA helicase | AT5G26742 | emb1138 | C_4 | increased in CSA-Pi9 sample sets |
| LOC_Os12g41715.1 | Q0ILZ4 | RNA.processing.RNA helicase | AT3G22330 | PMH2 | C_4 | increased in CSA-Pi9 sample sets |
| LOC_Os02g19860.1 | Q6K4U8 | RNA.processing.RNA helicase | AT3G26560 | - | C_4 | increased in CSA-Pi9 sample sets |
| LOC_Os01g68320.1 | Q5N7W4 | RNA.processing.RNA helicase | AT5G63120 | - | C_4 | increased in CSA-Pi9 sample sets |
| LOC_Os07g20580.1 | Q6YS30 | RNA.processing.RNA helicase | AT1G31970 | STRS1 | C_3 | increased in CSA-Pi9 sample sets |
| LOC_Os03g19960.1 | Q10MC7 | RNA.processing.RNA helicase | AT2G47250 | - | C_3 | increased in CSA-Pi9 sample sets |
| LOC_Os01g10050.1 | Q5QMN3 | RNA.processing.RNA helicase | AT1G55150 | - | C_4 | increased in CSA-Pi9 sample sets |
| LOC_Os01g36860.1 | Q5JKF2 | RNA.processing.RNA helicase | AT3G01540 | DRH1 | C_4 | increased in CSA-Pi9 sample sets |
| LOC_Os08g24760.1 | B9G0E3 | RNA.processing.RNA helicase | AT1G32490 | EMB2733 | C_3 | increased in CSA-Pi9 sample sets |
| LOC_Os07g05050.1 | B9FVG6 | RNA.processing.RNA helicase | AT3G22330 | PMH2 | C_4 | increased in CSA-Pi9 sample sets |
| LOC_Os07g32430.1 | Q7XIR8 | RNA.processing.RNA helicase | AT5G13010 | EMB3011 | C_4 | increased in CSA-Pi9 sample sets |
| LOC_Os01g65220.1 | Q5N739 | RNA.processing.ribonucleases | AT1G75660 | XRN3 | C_3 | increased in CSA-Pi9 sample sets |
| LOC_Os09g36700.1 | Q69JF4 | RNA.processing.ribonucleases | AT1G14220 | - | C_4 | increased in CSA-Pi9 sample sets |
| LOC_Os01g56550.1 | Q5N8X8 | RNA.transcription | AT1G75510 | - | C_4 | increased in CSA-Pi9 sample sets |
| LOC_Os02g49150.1 | B7F9H3 | RNA.transcription | AT5G09920 | RPB15.9 | C_4 | increased in CSA-Pi9 sample sets |
| LOC_Os01g57946.1 | Q8S1Q6 | RNA.transcription | ATCG00740 | RPOA | C_4 | increased in CSA-Pi9 sample sets |
| LOC_Os03g46190.1 | Q75GC3 | RNA.transcription | AT3G22590 | - | C_3 | increased in CSA-Pi9 sample sets |
| LOC_Os02g24614.1 | Q6Z1U9 | RNA.transcription | ATCG00180 | RPOC1 | C_3 | increased in CSA-Pi9 sample sets |
| LOC_Os06g48530.1 | Q5Z4U6 | RNA.regulation of transcription.C2H2 zinc finger family | AT2G27100 | SE | C_4 | increased in CSA-Pi9 sample sets |
| LOC_Os08g40560.1 | Q7EZ93 | RNA.regulation of transcription.C2H2 zinc finger family | AT2G27100 | SE | C_4 | increased in CSA-Pi9 sample sets |
| LOC_Os02g02390.1 | Q6Z7B3 | RNA.regulation of transcription.HSF,Heat-shock transcription factor family | AT1G12800 | - | C_4 | increased in CSA-Pi9 sample sets |
| LOC_Os05g41510.1 | B9FKX6 | RNA.regulation of transcription.Global transcription factor group | AT1G65440 | GTB1 | C_4 | increased in CSA-Pi9 sample sets |
| LOC_Os06g10620.1 | B9FS39 | RNA.regulation of transcription.Global transcription factor group | AT4G08350 | GTA02 | C_4 | increased in CSA-Pi9 sample sets |
| LOC_Os04g25550.1 | Q7X923 | RNA.regulation of transcription.Global transcription factor group | AT4G10710 | SPT16 | C_4 | increased in CSA-Pi9 sample sets |
| LOC_Os01g08970.1 | Q9LGR0 | RNA.regulation of transcription.Nucleosome/chromatin assembly factor group | AT3G28730 | ATHMG | C_4 | increased in CSA-Pi9 sample sets |
| LOC_Os01g44230.1 | Q9SBW2 | RNA.regulation of transcription.putative transcription regulator | AT3G48670 | - | C_4 | increased in CSA-Pi9 sample sets |
| LOC_Os05g43060.1 | B9FL11 | RNA.regulation of transcription.putative transcription regulator | AT5G04290 | KTF1 | C_4 | increased in CSA-Pi9 sample sets |
| LOC_Os05g42300.1 | Q60EI5 | RNA.regulation of transcription.putative transcription regulator | AT1G03530 | ATNAF1 | C_4 | increased in CSA-Pi9 sample sets |
| LOC_Os11g09990.1 | Q53LJ0 | RNA.regulation of transcription.unclassified | AT5G07900 | - | C_3 | increased in CSA-Pi9 sample sets |
| LOC_Os02g54200.1 | Q6K7E2 | RNA.regulation of transcription.unclassified | AT2G44020 | - | C_4 | increased in CSA-Pi9 sample sets |
| LOC_Os12g09580.1 | Q2QWE9 | RNA.regulation of transcription.unclassified | AT5G23570 | SGS3 | C_4 | increased in CSA-Pi9 sample sets |
| LOC_Os01g61830.1 | Q0JHZ2 | RNA.regulation of transcription.unclassified | AT2G20280 | - | C_4 | increased in CSA-Pi9 sample sets |
| LOC_Os05g43440.1 | Q6L4W0 | RNA.regulation of transcription.unclassified | AT4G11060 | MTSSB | C_4 | increased in CSA-Pi9 sample sets |
| LOC_Os04g42010.1 | Q7XQH8 | RNA.regulation of transcription.unclassified | AT4G32720 | AtLa1 | C_4 | increased in CSA-Pi9 sample sets |
| LOC_Os11g14300.1 | Q0ITJ6 | RNA.RNA binding | AT2G43970 | - | C_4 | increased in CSA-Pi9 sample sets |
| LOC_Os11g04390.1 | Q2RAS7 | RNA.RNA binding | AT3G52660 | - | C_4 | increased in CSA-Pi9 sample sets |
| LOC_Os03g17010.1 | Q10N90 | RNA.RNA binding | AT5G02530 | - | C_4 | increased in CSA-Pi9 sample sets |
| LOC_Os03g17710.1 | Q10N19 | RNA.RNA binding | AT2G37340 | RSZ33 | C_4 | increased in CSA-Pi9 sample sets |
| LOC_Os09g03610.1 | Q6K271 | RNA.RNA binding | AT4G16280 | FCA | C_4 | increased in CSA-Pi9 sample sets |
| LOC_Os07g27300.1 | Q84YS0 | RNA.RNA binding | AT5G51410 | - | C_4 | increased in CSA-Pi9 sample sets |
| LOC_Os10g30370.1 | Q337U3 | RNA.RNA binding | AT2G16940 | - | C_4 | increased in CSA-Pi9 sample sets |
| LOC_Os01g06290.1 | Q5ZCD9 | RNA.RNA binding | AT2G37340 | RSZ33 | C_3 | increased in CSA-Pi9 sample sets |
| LOC_Os01g16090.1 | Q9LJ04 | RNA.RNA binding | AT3G56860 | UBA2A | C_4 | increased in CSA-Pi9 sample sets |
| LOC_Os08g42980.1 | Q6ZJG0 | RNA.RNA binding | AT2G43970 | - | C_4 | increased in CSA-Pi9 sample sets |
| LOC_Os11g01869.1 | Q0IV63 | RNA.RNA binding | AT1G09700 | HYL1 | C_4 | increased in CSA-Pi9 sample sets |
| LOC_Os03g46610.1 | Q7Y183 | DNA.synthesis/chromatin structure | AT5G60990 | - | C_4 | increased in CSA-Pi9 sample sets |
| LOC_Os05g46230.1 | Q53WK4 | DNA.synthesis/chromatin structure | AT5G56950 | NFA03 | C_4 | increased in CSA-Pi9 sample sets |
| LOC_Os01g58680.1 | Q5VQJ9 | DNA.synthesis/chromatin structure | AT2G41460 | ARP | C_4 | increased in CSA-Pi9 sample sets |
| LOC_Os12g09120.1 | Q2QWJ7 | DNA.synthesis/chromatin structure | AT3G09100 | - | C_4 | increased in CSA-Pi9 sample sets |
| LOC_Os03g50090.1 | Q53RK8 | DNA.synthesis/chromatin structure | AT2G33730 | - | C_4 | increased in CSA-Pi9 sample sets |
| LOC_Os02g42860.1 | Q6H7S2 | DNA.synthesis/chromatin structure | AT4G00660 | - | C_4 | increased in CSA-Pi9 sample sets |
| LOC_Os01g68320.1 | Q5N7W4 | DNA.synthesis/chromatin structure | AT5G63120 | - | C_4 | increased in CSA-Pi9 sample sets |
| LOC_Os07g20580.1 | Q6YS30 | DNA.synthesis/chromatin structure | AT1G31970 | STRS1 | C_3 | increased in CSA-Pi9 sample sets |
| LOC_Os10g32540.1 | Q8H902 | DNA.synthesis/chromatin structure | AT3G04260 | PTAC3 | C_3 | increased in CSA-Pi9 sample sets |
| LOC_Os07g45360.1 | Q5K5B6 | DNA.synthesis/chromatin structure | AT3G09720 | - | C_4 | increased in CSA-Pi9 sample sets |
| LOC_Os01g10050.1 | Q5QMN3 | DNA.synthesis/chromatin structure | AT1G55150 | - | C_4 | increased in CSA-Pi9 sample sets |
| LOC_Os03g25450.1 | Q10KT9 | DNA.synthesis/chromatin structure | AT3G57150 | NAP57 | C_4 | increased in CSA-Pi9 sample sets |
| LOC_Os06g05660.1 | Q5VND6 | DNA.synthesis/chromatin structure | AT2G19480 | NFA02 | C_4 | increased in CSA-Pi9 sample sets |
| LOC_Os07g44190.1 | Q8H5T5 | DNA.synthesis/chromatin structure | AT3G57150 | NAP57 | C_4 | increased in CSA-Pi9 sample sets |
| LOC_Os04g45040.1 | Q7XMK8 | DNA.synthesis/chromatin structure | AT4G00660 | - | C_4 | increased in CSA-Pi9 sample sets |
| LOC_Os02g49270.1 | A0A0P0VP26 | DNA.synthesis/chromatin structure | AT4G26600 | - | C_4 | increased in CSA-Pi9 sample sets |
| LOC_Os10g35990.1 | Q109G2 | DNA.synthesis/chromatin structure | AT4G00660 | - | C_4 | increased in CSA-Pi9 sample sets |
| LOC_Os01g53560.1 | Q5JND9 | DNA.repair | AT3G14890 | - | C_4 | increased in CSA-Pi9 sample sets |
| LOC_Os09g02270.1 | Q6YX89 | DNA.unspecified | AT4G35220 | - | C_2 | increased in CSA-Pi9 sample sets |
| LOC_Os02g33500.1 | Q6ER90 | protein.aa activation.threonine-tRNA ligase | AT2G04842 | EMB2761 | C_2 | increased in CSA-Pi9 sample sets |
| LOC_Os03g38980.1 | Q6F2U9 | protein.aa activation.lysine-tRNA ligase | AT3G11710 | ATKRS-1 | C_4 | increased in CSA-Pi9 sample sets |
| LOC_Os07g06940.1 | B9FVM5 | protein.aa activation.valine-tRNA ligase | AT5G16715 | EMB2247 | C_2 | increased in CSA-Pi9 sample sets |
| LOC_Os03g11120.1 | Q10Q53 | protein.aa activation.methionine-tRNA ligase | AT3G55400 | OVA1 | C_2 | increased in CSA-Pi9 sample sets |
| LOC_Os02g02860.1 | Q6YUS0 | protein.aa activation.glutamate-tRNA ligase | AT5G64050 | ATERS | C_2 | increased in CSA-Pi9 sample sets |
| LOC_Os01g09000.1 | Q5VRX8 | protein.aa activation.glutamine-tRNA ligase | AT1G25350 | OVA9 | C_2 | increased in CSA-Pi9 sample sets |
| LOC_Os02g51830.1 | Q6Z695 | protein.aa activation.histidine-tRNA ligase | AT3G46100 | ATHRS1 | C_4 | increased in CSA-Pi9 sample sets |
| LOC_Os04g16738.1 | E9KIP2 | protein.synthesis.ribosomal protein.prokaryotic.chloroplast.30S subunit.S4 | ATCG00380 | RPS4 | C_4 | increased in CSA-Pi9 sample sets |
| LOC_Os03g34040.1 | Q850W6 | protein.synthesis.ribosomal protein.prokaryotic.chloroplast.30S subunit.S5 | AT2G33800 | - | C_4 | increased in CSA-Pi9 sample sets |
| LOC_Os03g63950.1 | Q84M68 | protein.synthesis.ribosomal protein.prokaryotic.chloroplast.30S subunit.S30A | AT5G24490 | - | C_4 | increased in CSA-Pi9 sample sets |
| LOC_Os03g03360.1 | Q9ZST0 | protein.synthesis.ribosomal protein.prokaryotic.chloroplast.50S subunit.L5 | AT4G01310 | - | C_4 | increased in CSA-Pi9 sample sets |
| LOC_Os03g24020.1 | Q10L93 | protein.synthesis.ribosomal protein.prokaryotic.chloroplast.50S subunit.L6 | AT1G05190 | emb2394 | C_4 | increased in CSA-Pi9 sample sets |
| LOC_Os02g57670.1 | Q6KA00 | protein.synthesis.ribosomal protein.prokaryotic.chloroplast.50S subunit.L9 | AT3G44890 | RPL9 | C_4 | increased in CSA-Pi9 sample sets |
| LOC_Os03g17580.1 | Q10N30 | protein.synthesis.ribosomal protein.prokaryotic.chloroplast.50S subunit.L10 | AT5G13510 | - | C_3 | increased in CSA-Pi9 sample sets |
| LOC_Os03g03020.1 | Q9AYT6 | protein.synthesis.ribosomal protein.prokaryotic.chloroplast.50S subunit.L11 | AT1G32990 | PRPL11 | C_4 | increased in CSA-Pi9 sample sets |
| LOC_Os01g57958.1 | E9KIR7 | protein.synthesis.ribosomal protein.prokaryotic.chloroplast.50S subunit.L16 | ATCG00790 | RPL16 | C_4 | increased in CSA-Pi9 sample sets |
| LOC_Os03g61260.1 | Q8SAY0 | protein.synthesis.ribosomal protein.prokaryotic.chloroplast.50S subunit.L18 | AT1G48350 | - | C_4 | increased in CSA-Pi9 sample sets |
| LOC_Os02g43600.1 | Q6H8H3 | protein.synthesis.ribosomal protein.prokaryotic.chloroplast.50S subunit.L19 | AT4G17560 | - | C_3 | increased in CSA-Pi9 sample sets |
| LOC_Os06g46930.1 | Q653V9 | protein.synthesis.ribosomal protein.prokaryotic.chloroplast.50S subunit.L24 | AT5G54600 | - | C_3 | increased in CSA-Pi9 sample sets |
| LOC_Os03g26860.1 | Q10KG1 | protein.synthesis.ribosomal protein.prokaryotic.mitochondrion.30S subunit.S11 | AT1G31817 | NFD3 | C_4 | increased in CSA-Pi9 sample sets |
| LOC_Os09g08890.1 | Q67TM6 | protein.synthesis.ribosomal protein.prokaryotic.mitochondrion.50S subunit.L5 | ATMG00210 | RPL5 | C_4 | increased in CSA-Pi9 sample sets |
| LOC_Os02g01230.1 | Q6YU81 | protein.synthesis.ribosomal protein.prokaryotic.unknown organellar.30S subunit.S5 | AT1G64880 | - | C_4 | increased in CSA-Pi9 sample sets |
| LOC_Os10g35690.1 | Q8LNG5 | protein.synthesis.ribosomal protein.prokaryotic.unknown organellar.30S subunit.S18 | AT1G07210 | - | C_4 | increased in CSA-Pi9 sample sets |
| LOC_Os07g41750.1 | Q6YTY2 | protein.synthesis.ribosomal protein.eukaryotic.40S subunit.S3 | AT2G31610 | - | C_3 | increased in CSA-Pi9 sample sets |
| LOC_Os03g38000.1 | Q75G91 | protein.synthesis.ribosomal protein.eukaryotic.40S subunit.S3 | AT5G35530 | - | C_3 | increased in CSA-Pi9 sample sets |
| LOC_Os07g42950.1 | Q8LH97 | protein.synthesis.ribosomal protein.eukaryotic.40S subunit.S6 | AT5G10360 | EMB3010 | C_3 | increased in CSA-Pi9 sample sets |
| LOC_Os05g27940.1 | Q8LJU5 | protein.synthesis.ribosomal protein.eukaryotic.40S subunit.S7 | AT1G48830 | - | C_3 | increased in CSA-Pi9 sample sets |
| LOC_Os03g18570.1 | Q10MS5 | protein.synthesis.ribosomal protein.eukaryotic.40S subunit.S7 | AT1G48830 | - | C_3 | increased in CSA-Pi9 sample sets |
| LOC_Os03g26229.1 | Q0DRI8 | protein.synthesis.ribosomal protein.eukaryotic.40S subunit.S9 | AT5G15200 | - | C_3 | increased in CSA-Pi9 sample sets |
| LOC_Os03g05980.1 | Q2R1J8 | protein.synthesis.ribosomal protein.eukaryotic.40S subunit.S9 | AT5G39850 | - | C_3 | increased in CSA-Pi9 sample sets |
| LOC_Os04g42380.1 | Q0JBY8 | protein.synthesis.ribosomal protein.eukaryotic.40S subunit.S11 | AT3G48930 | EMB1080 | C_3 | increased in CSA-Pi9 sample sets |
| LOC_Os02g06700.2 | Q6H7T1 | protein.synthesis.ribosomal protein.eukaryotic.40S subunit.S14 | AT3G52580 | - | C_3 | increased in CSA-Pi9 sample sets |
| LOC_Os03g58050.1 | Q8H590 | protein.synthesis.ribosomal protein.eukaryotic.40S subunit.S18 | AT4G09800 | RPS18C | C_3 | increased in CSA-Pi9 sample sets |
| LOC_Os03g46490.1 | Q7Y199 | protein.synthesis.ribosomal protein.eukaryotic.40S subunit.S21 | AT5G27700 | - | C_3 | increased in CSA-Pi9 sample sets |
| LOC_Os01g52490.1 | Q8W0C2 | protein.synthesis.ribosomal protein.eukaryotic.40S subunit.S24 | AT5G28060 | - | C_3 | increased in CSA-Pi9 sample sets |
| LOC_Os05g39960.1 | Q0DHB9 | protein.synthesis.ribosomal protein.eukaryotic.40S subunit.S26 | AT3G56340 | - | C_4 | increased in CSA-Pi9 sample sets |
| LOC_Os01g60790.1 | P49216 | protein.synthesis.ribosomal protein.eukaryotic.40S subunit.S26 | AT3G56340 | - | C_4 | increased in CSA-Pi9 sample sets |
| LOC_Os12g21798.1 | Q0INR5 | protein.synthesis.ribosomal protein.eukaryotic.40S subunit.S3A | AT4G34670 | - | C_4 | increased in CSA-Pi9 sample sets |
| LOC_Os02g18550.1 | Q6K8B8 | protein.synthesis.ribosomal protein.eukaryotic.40S subunit.S3A | AT4G34670 | - | C_4 | increased in CSA-Pi9 sample sets |
| LOC_Os03g10340.1 | P49397 | protein.synthesis.ribosomal protein.eukaryotic.40S subunit.S3A | AT4G34670 | - | C_4 | increased in CSA-Pi9 sample sets |
| LOC_Os02g27760.1 | Q6K5R6 | protein.synthesis.ribosomal protein.eukaryotic.40S subunit.S15A | AT5G59850 | - | C_4 | increased in CSA-Pi9 sample sets |
| LOC_Os09g08890.1 | Q67TM6 | protein.synthesis.ribosomal protein.prokaryotic.mitochondrion.50S subunit.L5 | ATMG00210 | RPL5 | C_4 | increased in CSA-Pi9 sample sets |
| LOC_Os01g67134.1 | B7EWY9 | protein.synthesis.ribosomal protein.eukaryotic.60S subunit.L5 | AT3G25520 | ATL5 | C_3 | increased in CSA-Pi9 sample sets |
| LOC_Os02g37862.1 | Q6YY64 | protein.synthesis.ribosomal protein.eukaryotic.60S subunit.L6 | AT1G74050 | - | C_4 | increased in CSA-Pi9 sample sets |
| LOC_Os03g51510.1 | Q6AU10 | protein.synthesis.ribosomal protein.eukaryotic.60S subunit.L6 | AT2G18400 | - | C_3 | increased in CSA-Pi9 sample sets |
| LOC_Os04g39700.1 | Q7XR19 | protein.synthesis.ribosomal protein.eukaryotic.60S subunit.L6 | AT1G74050 | - | C_4 | increased in CSA-Pi9 sample sets |
| LOC_Os04g51630.1 | Q0JAC6 | protein.synthesis.ribosomal protein.eukaryotic.60S subunit.L7 | AT3G13580 | - | C_3 | increased in CSA-Pi9 sample sets |
| LOC_Os08g13690.1 | Q6YYR2 | protein.synthesis.ribosomal protein.eukaryotic.60S subunit.L7 | AT3G13580 | - | C_3 | increased in CSA-Pi9 sample sets |
| LOC_Os02g01332.1 | Q0E4R7 | protein.synthesis.ribosomal protein.eukaryotic.60S subunit.L9 | AT1G33140 | PGY2 | C_3 | increased in CSA-Pi9 sample sets |
| LOC_Os01g10820.1 | Q9LWN1 | protein.synthesis.ribosomal protein.eukaryotic.60S subunit.L11 | AT5G45775 | - | C_3 | increased in CSA-Pi9 sample sets |
| LOC_Os02g47140.1 | Q6Z8E0 | protein.synthesis.ribosomal protein.eukaryotic.60S subunit.L12 | AT2G37190 | - | C_4 | increased in CSA-Pi9 sample sets |
| LOC_Os02g40880.1 | Q6K1Q6 | protein.synthesis.ribosomal protein.eukaryotic.60S subunit.L14 | AT2G20450 | - | C_3 | increased in CSA-Pi9 sample sets |
| LOC_Os03g22340.1 | Q762A6 | protein.synthesis.ribosomal protein.eukaryotic.60S subunit.L22 | AT3G05560 | - | C_4 | increased in CSA-Pi9 sample sets |
| LOC_Os02g56960.1 | Q9AV77 | protein.synthesis.ribosomal protein.eukaryotic.60S subunit.L23 | AT3G04400 | emb2171 | C_4 | increased in CSA-Pi9 sample sets |
| LOC_Os02g18380.1 | Q6K4T1 | protein.synthesis.ribosomal protein.eukaryotic.60S subunit.L27 | AT4G15000 | - | C_3 | increased in CSA-Pi9 sample sets |
| LOC_Os10g41470.1 | Q7XC31 | protein.synthesis.ribosomal protein.eukaryotic.60S subunit.L27 | AT4G15000 | - | C_3 | increased in CSA-Pi9 sample sets |
| LOC_Os02g48660.1 | Q6ZGV5 | protein.synthesis.ribosomal protein.eukaryotic.60S subunit.L31 | AT2G19740 | - | C_3 | increased in CSA-Pi9 sample sets |
| LOC_Os08g23710.1 | P0DKK7 | protein.synthesis.ribosomal protein.eukaryotic.60S subunit.L7A | AT3G62870 | - | C_3 | increased in CSA-Pi9 sample sets |
| LOC_Os03g13800.1 | Q10PA5 | protein.synthesis.ribosomal protein.eukaryotic.60S subunit.L7A | AT5G20160 | - | C_3 | increased in CSA-Pi9 sample sets |
| LOC_Os08g02340.1 | Q69UI8 | protein.synthesis.ribosomal protein.eukaryotic.60S subunit.P1 | AT5G24510 | - | C_4 | increased in CSA-Pi9 sample sets |
| LOC_Os01g54870.1 | Q943F3 | protein.synthesis.ribosomal protein.eukaryotic.60S subunit.L18A | AT1G29970 | - | C_3 | increased in CSA-Pi9 sample sets |
| LOC_Os01g47660.1 | Q0JKK9 | protein.synthesis.ribosomal protein.eukaryotic.60S subunit.L18A | AT1G29970 | - | C_3 | increased in CSA-Pi9 sample sets |
| LOC_Os02g55370.1 | P51426 | protein.synthesis.ribosomal protein.unknown.large subunit.L39 | - | - | C_4 | increased in CSA-Pi9 sample sets |
| LOC_Os05g49970.2 | Q0DFR3 | protein.synthesis.initiation | AT1G17220 | FUG1 | C_4 | increased in CSA-Pi9 sample sets |
| LOC_Os09g15770.1 | Q6K2P9 | protein.synthesis.initiation | AT1G36730 | - | C_4 | increased in CSA-Pi9 sample sets |
| LOC_Os05g49970.1 | B9G7K8 | protein.synthesis.initiation | AT1G17220 | FUG1 | C_4 | increased in CSA-Pi9 sample sets |
| LOC_Os07g03230.1 | Q84LG6 | protein.synthesis.initiation | AT3G56150 | EIF3C | C_4 | increased in CSA-Pi9 sample sets |
| LOC_Os09g34010.1 | Q69IM0 | protein.synthesis.initiation | AT4G11160 | - | C_4 | increased in CSA-Pi9 sample sets |
| LOC_Os06g23440.1 | Q7Y1V2 | protein.synthesis.initiation | AT2G04520 | - | C_4 | increased in CSA-Pi9 sample sets |
| LOC_Os04g42140.1 | Q84PB3 | protein.synthesis.initiation | AT5G57870 | - | C_4 | increased in CSA-Pi9 sample sets |
| LOC_Os03g21550.1 | Q10LV9 | protein.synthesis.initiation | AT5G20920 | EIF2 BETA | C_4 | increased in CSA-Pi9 sample sets |
| LOC_Os07g40580.1 | Q84ZK1 | protein.synthesis.initiation | AT1G13950 | EIF-5A | C_4 | increased in CSA-Pi9 sample sets |
| LOC_Os12g32240.1 | Q2QQ48 | protein.synthesis.initiation | AT1G13950 | EIF-5A | C_3 | increased in CSA-Pi9 sample sets |
| LOC_Os07g02210.1 | Q8GRL5 | protein.synthesis.initiation | AT1G13950 | EIF-5A | C_3 | increased in CSA-Pi9 sample sets |
| LOC_Os02g39350.1 | B9F0Z2 | protein.synthesis.initiation | AT1G73180 | - | C_4 | increased in CSA-Pi9 sample sets |
| LOC_Os03g60130.1 | Q84TW3 | protein.synthesis.elongation | AT2G38560 | TFIIS | C_4 | increased in CSA-Pi9 sample sets |
| LOC_Os01g71230.1 | Q8RUI4 | protein.synthesis.elongation | AT3G12390 | - | C_4 | increased in CSA-Pi9 sample sets |
| LOC_Os08g09350.1 | Q6Z1C0 | protein.synthesis.ribosomal RNA | AT3G18610 | ATRANGAP1 | C_4 | increased in CSA-Pi9 sample sets |
| LOC_Os04g52960.1 | Q7XTT4 | protein.synthesis.ribosomal RNA | AT1G48920 | ATNUC-L1 | C_4 | increased in CSA-Pi9 sample sets |
| LOC_Os01g69250.1 | Q5JJI4 | protein.targeting.mitochondria | AT1G27390 | TOM20-2 | C_4 | increased in CSA-Pi9 sample sets |
| LOC_Os03g20750.1 | Q10M45 | protein.targeting.mitochondria | AT3G20000 | TOM40 | C_2 | increased in CSA-Pi9 sample sets |
| LOC_Os05g02060.1 | Q65XV6 | protein.targeting.mitochondria | AT2G28900 | OEP16 | C_3 | increased in CSA-Pi9 sample sets |
| LOC_Os01g21820.1 | Q657P0 | protein.targeting.chloroplast | AT4G01800 | - | C_3 | increased in CSA-Pi9 sample sets |
| LOC_Os02g51810.1 | Q6Z697 | protein.targeting.chloroplast | AT5G09420 | ATTOC64-V | C_2 | increased in CSA-Pi9 sample sets |
| LOC_Os11g05556.1 | Q53QG1 | protein.targeting.chloroplast | AT5G03940 | FFC | C_4 | increased in CSA-Pi9 sample sets |
| LOC_Os08g43470.1 | Q6YT06 | protein.targeting.secretory pathway.ER | AT4G38790 | - | C_3 | increased in CSA-Pi9 sample sets |
| LOC_Os03g53140.1 | Q75KX1 | protein.targeting.secretory pathway.golgi | AT5G43670 | - | C_3 | increased in CSA-Pi9 sample sets |
| LOC_Os02g21250.1 | Q6Z844 | protein.targeting.secretory pathway.unspecified | AT3G09800 | - | C_3 | increased in CSA-Pi9 sample sets |
| LOC_Os08g37444.1 | Q6Z246 | protein.targeting.secretory pathway.unspecified | AT4G30600 | - | C_4 | increased in CSA-Pi9 sample sets |
| LOC_Os08g18110.1 | Q6YS11 | protein.targeting.secretory pathway.unspecified | AT3G56190 | ALPHA-SNAP2 | C_3 | increased in CSA-Pi9 sample sets |
| LOC_Os01g66560.1 | Q5N842 | protein.targeting.secretory pathway.unspecified | AT1G67680 | - | C_4 | increased in CSA-Pi9 sample sets |
| LOC_Os02g49980.1 | Q6Z2K0 | protein.targeting.secretory pathway.unspecified | AT5G37680 | ATARLA1A | C_3 | increased in CSA-Pi9 sample sets |
| LOC_Os06g23430.1 | P49964 | protein.targeting.secretory pathway.unspecified | AT1G48160 | - | C_4 | increased in CSA-Pi9 sample sets |
| LOC_Os05g38590.1 | Q0DHK0 | protein.postranslational modification | AT5G59770 | - | C_2 | increased in CSA-Pi9 sample sets |
| LOC_Os07g23110.1 | Q7EYV7 | protein.postranslational modification | AT2G31320 | PARP2 | C_4 | increased in CSA-Pi9 sample sets |
| LOC_Os01g05694.1 | Q5ZEJ0 | protein.postranslational modification | AT5G10480 | PAS2 | C_2 | increased in CSA-Pi9 sample sets |
| LOC_Os02g38230.1 | Q6ZI50 | protein.postranslational modification | AT5G50200 | WR3 | C_2 | increased in CSA-Pi9 sample sets |
| LOC_Os05g36070.1 | B9FJ61 | protein.degradation | AT2G03120 | ATSPP | C_2 | increased in CSA-Pi9 sample sets |
| LOC_Os12g40550.1 | Q2QMN7 | protein.degradation | AT5G43600 | ATAAH-2 | C_2 | increased in CSA-Pi9 sample sets |
| LOC_Os01g70780.1 | Q8LR61 | protein.degradation.autophagy | AT3G62770 | AtATG18a | C_2 | increased in CSA-Pi9 sample sets |
| LOC_Os03g52070.1 | Q10DG0 | protein.degradation.serine protease | AT1G33540 | scpl18 | C_2 | increased in CSA-Pi9 sample sets |
| LOC_Os03g52040.2 | Q851E7 | protein.degradation.serine protease | AT1G73280 | scpl3 | C_2 | increased in CSA-Pi9 sample sets |
| LOC_Os05g49380.1 | Q6AUN5 | protein.degradation.serine protease | AT3G27925 | DEGP1 | C_3 | increased in CSA-Pi9 sample sets |
| LOC_Os04g25550.1 | Q7X923 | protein.degradation.metalloprotease | AT4G10710 | SPT16 | C_4 | increased in CSA-Pi9 sample sets |
| LOC_Os09g24250.1 | Q69MX3 | protein.degradation.ubiquitin.ubiquitin | AT4G22285 | - | C_3 | increased in CSA-Pi9 sample sets |
| LOC_Os05g19970.1 | Q0DJH5 | protein.degradation.ubiquitin.E3.RING | AT3G62240 | - | C_4 | increased in CSA-Pi9 sample sets |
| LOC_Os05g38370.1 | Q65XG1 | protein.folding | AT3G55520 | - | C_4 | increased in CSA-Pi9 sample sets |
| LOC_Os05g47880.1 | Q6L4F9 | protein.glycosylation | AT5G57500 | - | C_2 | increased in CSA-Pi9 sample sets |
| LOC_Os08g29710.1 | Q6ZA16 | protein.glycosylation | AT5G53340 | - | C_2 | increased in CSA-Pi9 sample sets |
| LOC_Os09g14520.1 | Q69M22 | protein.glycosylation | AT5G19980 | - | C_2 | increased in CSA-Pi9 sample sets |
| LOC_Os09g26310.1 | Q69MI0 | protein.glycosylation | AT5G57500 | - | C_3 | increased in CSA-Pi9 sample sets |
| LOC_Os12g04220.1 | Q2QY12 | signaling.calcium | AT2G41560 | ACA4 | C_2 | increased in CSA-Pi9 sample sets |
| LOC_Os03g53670.1 | Q10CW7 | signaling.calcium | AT3G13460 | ECT2 | C_4 | increased in CSA-Pi9 sample sets |
| LOC_Os03g06240.1 | Q10RI3 | signaling.calcium | AT3G13460 | ECT2 | C_4 | increased in CSA-Pi9 sample sets |
| LOC_Os08g40530.1 | B9G1S8 | signaling.calcium | AT5G57110 | ACA8 | C_2 | increased in CSA-Pi9 sample sets |
| LOC_Os07g07490.1 | Q69LD0 | signaling.calcium | AT5G61020 | ECT3 | C_4 | increased in CSA-Pi9 sample sets |
| LOC_Os07g44040.1 | Q8LHP5 | signaling.G-proteins | AT5G47520 | AtRABA5a | C_3 | increased in CSA-Pi9 sample sets |
| LOC_Os03g60870.1 | Q852B4 | signaling.G-proteins | AT1G07410 | ATRABA2B | C_3 | increased in CSA-Pi9 sample sets |
| LOC_Os05g48980.1 | Q6AUF8 | signaling.G-proteins | AT5G60860 | AtRABA1f | C_3 | increased in CSA-Pi9 sample sets |
| LOC_Os05g01480.1 | Q75M17 | signaling.G-proteins | AT1G09630 | ATRAB11C | C_3 | increased in CSA-Pi9 sample sets |
| LOC_Os03g42770.1 | Q75LV5 | signaling.G-proteins | AT4G05410 | - | C_4 | increased in CSA-Pi9 sample sets |
| LOC_Os08g09350.1 | Q6Z1C0 | signaling.G-proteins | AT3G18610 | ATRANGAP1 | C_4 | increased in CSA-Pi9 sample sets |
| LOC_Os10g23100.1 | Q338Z5 | signaling.G-proteins | AT5G65270 | AtRABA4a | C_3 | increased in CSA-Pi9 sample sets |
| LOC_Os07g43470.1 | Q7XI68 | signaling.G-proteins | AT4G39520 | - | C_4 | increased in CSA-Pi9 sample sets |
| LOC_Os01g15010.1 | Q9SDK4 | signaling.G-proteins | AT4G02080 | ASAR1 | C_3 | increased in CSA-Pi9 sample sets |
| LOC_Os05g28290.1 | Q0DIY4 | signaling.G-proteins | AT5G58590 | RANBP1 | C_4 | increased in CSA-Pi9 sample sets |
| LOC_Os12g43550.2 | Q0ILQ6 | signaling.G-proteins | AT5G45130 | ATRAB5A | C_3 | increased in CSA-Pi9 sample sets |
| LOC_Os04g52960.1 | Q7XTT4 | signaling.G-proteins | AT1G48920 | ATNUC-L1 | C_4 | increased in CSA-Pi9 sample sets |
| LOC_Os06g35814.1 | P40393 | signaling.G-proteins | AT4G18800 | ATHSGBP | C_3 | increased in CSA-Pi9 sample sets |
| LOC_Os04g35100.1 | Q0JD56 | signaling.MAP kinases | AT1G49740 | - | C_2 | increased in CSA-Pi9 sample sets |
| LOC_Os03g30130.1 | Q75IX4 | signaling.MAP kinases | AT1G13680 | - | C_2 | increased in CSA-Pi9 sample sets |
| LOC_Os11g34450.1 | Q2R2W2 | signaling.14-3-3 proteins | AT3G02520 | GRF7 | C_3 | increased in CSA-Pi9 sample sets |
| LOC_Os08g37490.1 | Q84J55 | signaling.14-3-3 proteins | AT1G78300 | GRF2 | C_3 | increased in CSA-Pi9 sample sets |
| LOC_Os03g50290.1 | Q06967 | signaling.14-3-3 proteins | AT3G02520 | GRF7 | C_3 | increased in CSA-Pi9 sample sets |
| LOC_Os04g38870.1 | Q0JCM0 | signaling.14-3-3 proteins | AT3G02520 | GRF7 | C_3 | increased in CSA-Pi9 sample sets |
| LOC_Os02g36974.1 | Q6EUP4 | signaling.14-3-3 proteins | AT3G02520 | GRF7 | C_3 | increased in CSA-Pi9 sample sets |
| LOC_Os04g43580.1 | Q7XRS1 | signaling.light | AT2G31160 | LSH3 | C_3 | increased in CSA-Pi9 sample sets |
| LOC_Os11g03050.1 | A0A0N7KSC9 | cell.division | AT2G38670 | PECT1 | C_2 | increased in CSA-Pi9 sample sets |
| LOC_Os04g41300.1 | Q7X8U6 | cell.division | AT3G01800 | - | C_3 | increased in CSA-Pi9 sample sets |
| LOC_Os01g68710.1 | Q5N801 | cell.cycle.peptidylprolyl isomerase | AT5G48580 | FKBP15-2 | C_3 | increased in CSA-Pi9 sample sets |
| LOC_Os09g32526.1 | A3C0C6 | cell.cycle.peptidylprolyl isomerase | AT3G25220 | FKBP15-1 | C_4 | increased in CSA-Pi9 sample sets |
| LOC_Os09g24540.1 | Q6ESK5 | cell.cycle.peptidylprolyl isomerase | AT1G26550 | - | C_4 | increased in CSA-Pi9 sample sets |
| LOC_Os06g49480.1 | Q5Z9H8 | cell.cycle.peptidylprolyl isomerase | AT5G58710 | ROC7 | C_4 | increased in CSA-Pi9 sample sets |
| LOC_Os04g28420.1 | Q9XFE4 | cell.cycle.peptidylprolyl isomerase | AT3G25230 | ROF1 | C_4 | increased in CSA-Pi9 sample sets |
| LOC_Os07g08190.1 | B9FVT7 | cell.cycle.peptidylprolyl isomerase | AT3G63400 | - | C_4 | increased in CSA-Pi9 sample sets |
| LOC_Os09g04670.1 | Q6K456 | development.unspecified | AT3G15000 | - | C_4 | increased in CSA-Pi9 sample sets |
| LOC_Os10g02630.1 | Q33BE1 | development.unspecified | AT3G50670 | U1-70K | C_4 | increased in CSA-Pi9 sample sets |
| LOC_Os11g03050.1 | A0A0N7KSC9 | development.unspecified | AT2G38670 | PECT1 | C_2 | increased in CSA-Pi9 sample sets |
| LOC_Os06g08110.1 | Q8H613 | development.unspecified | AT1G80530 | - | C_2 | increased in CSA-Pi9 sample sets |
| LOC_Os09g03610.1 | Q6K271 | development.unspecified | AT4G16280 | FCA | C_4 | increased in CSA-Pi9 sample sets |
| LOC_Os11g11020.1 | Q2R8U1 | development.unspecified | AT1G11430 | - | C_4 | increased in CSA-Pi9 sample sets |
| LOC_Os07g25440.1 | Q0D6V7 | development.unspecified | AT2G40360 | - | C_4 | increased in CSA-Pi9 sample sets |
| LOC_Os01g69970.1 | Q5JJP8 | development.unspecified | AT4G18905 | - | C_4 | increased in CSA-Pi9 sample sets |
| LOC_Os04g50660.1 | Q0JAK5 | development.unspecified | AT5G16750 | TOZ | C_4 | increased in CSA-Pi9 sample sets |
| LOC_Os06g12820.1 | A0A0P0WV15 | development.unspecified | AT3G28150 | - | C_2 | increased in CSA-Pi9 sample sets |
| LOC_Os06g08310.1 | Q5SMI5 | transport.p- and v-ATPases | AT2G07560 | AHA6 | C_2 | increased in CSA-Pi9 sample sets |
| LOC_Os05g25550.1 | B9FNV9 | transport.p- and v-ATPases | AT2G07560 | AHA6 | C_2 | increased in CSA-Pi9 sample sets |
| LOC_Os01g40470.1 | Q8RU33 | transport.p- and v-ATPases.H+-transporting two-sector ATPase | AT3G28715 | - | C_3 | increased in CSA-Pi9 sample sets |
| LOC_Os05g49270.1 | Q688W0 | transport.sugars | AT1G75220 | - | C_2 | increased in CSA-Pi9 sample sets |
| LOC_Os10g39440.1 | Q336W8 | transport.sugars | AT4G35300 | - | C_2 | increased in CSA-Pi9 sample sets |
| LOC_Os05g50280.1 | Q688U6 | transport.sugars | AT1G75220 | - | C_2 | increased in CSA-Pi9 sample sets |
| LOC_Os01g51770.1 | Q0JJV1 | transport.sugars | AT3G01280 | VDAC1 | C_2 | increased in CSA-Pi9 sample sets |
| LOC_Os07g39350.1 | Q0D551 | transport.sugars | AT3G18830 | ATPLT5 | C_2 | increased in CSA-Pi9 sample sets |
| LOC_Os12g44380.1 | G3E4M9 | transport.sugars.sucrose | AT1G09960 | SUT4 | C_2 | increased in CSA-Pi9 sample sets |
| LOC_Os06g43660.1 | Q67WN5 | transport.amino acids | AT1G15690 | AVP1 | C_2 | increased in CSA-Pi9 sample sets |
| LOC_Os08g03350.1 | Q6ZK52 | transport.amino acids | AT5G40780 | LHT1 | C_2 | increased in CSA-Pi9 sample sets |
| LOC_Os02g55890.1 | Q75U53 | transport.amino acids | AT1G15690 | AVP1 | C_2 | increased in CSA-Pi9 sample sets |
| LOC_Os06g16420.1 | Q9FNZ8 | transport.amino acids | AT3G30390 | - | C_2 | increased in CSA-Pi9 sample sets |
| LOC_Os02g38230.1 | Q6ZI50 | transport.nitrate | AT5G50200 | WR3 | C_2 | increased in CSA-Pi9 sample sets |
| LOC_Os06g05160.1 | Q5VQ79 | transport.sulphate | AT3G15990 | SULTR3;4 | C_2 | increased in CSA-Pi9 sample sets |
| LOC_Os10g30790.1 | Q8H6G8 | transport.phosphate | AT2G38940 | ATPT2 | C_2 | increased in CSA-Pi9 sample sets |
| LOC_Os05g41480.1 | Q65X78 | transport.metabolite transporters at the envelope membrane | AT5G11230 | - | C_2 | increased in CSA-Pi9 sample sets |
| LOC_Os03g17740.1 | Q8GZZ6 | transport.metabolite transporters at the envelope membrane | AT5G05820 | - | C_2 | increased in CSA-Pi9 sample sets |
| LOC_Os08g25624.1 | Q84QU8 | transport.metabolite transporters at the envelope membrane | AT5G33320 | CUE1 | C_2 | increased in CSA-Pi9 sample sets |
| LOC_Os07g33954.1 | Q7F1T0 | transport.metabolite transporters at the envelope membrane | AT1G61800 | GPT2 | C_2 | increased in CSA-Pi9 sample sets |
| LOC_Os08g08840.1 | Q6YZC3 | transport.metabolite transporters at the envelope membrane | AT1G61800 | GPT2 | C_2 | increased in CSA-Pi9 sample sets |
| LOC_Os01g32980.1 | B9EX68 | transport.metabolite transporters at the mitochondrial membrane | AT1G25380 | - | C_2 | increased in CSA-Pi9 sample sets |
| LOC_Os05g23720.1 | Q0DJC0 | transport.metabolite transporters at the mitochondrial membrane | AT4G28390 | AAC3 | C_2 | increased in CSA-Pi9 sample sets |
| LOC_Os02g44680.1 | Q6ESG6 | transport.nucleotides | AT5G03555 | - | C_2 | increased in CSA-Pi9 sample sets |
| LOC_Os09g02214.1 | A0A0P0XJN2 | transport.metal | AT3G19490 | ATNHD1 | C_2 | increased in CSA-Pi9 sample sets |
| LOC_Os01g56420.1 | Q94EE4 | transport.metal | AT2G26975 | - | C_2 | increased in CSA-Pi9 sample sets |
| LOC_Os10g42870.1 | Q7XBS8 | transport.peptides and oligopeptides | AT2G02040 | ATPTR2-B | C_2 | increased in CSA-Pi9 sample sets |
| LOC_Os11g17970.1 | Q2R733 | transport.peptides and oligopeptides | AT5G01180 | PTR5 | C_2 | increased in CSA-Pi9 sample sets |
| LOC_Os02g46860.1 | B9F1X8 | transport.peptides and oligopeptides | AT5G64410 | ATOPT4 | C_2 | increased in CSA-Pi9 sample sets |
| LOC_Os10g33170.1 | Q7XDJ1 | transport.peptides and oligopeptides | AT5G46050 | ATPTR3 | C_2 | increased in CSA-Pi9 sample sets |
| LOC_Os08g05910.1 | Q6ZJR2 | transport.peptides and oligopeptides | AT1G12110 | NRT1.1 | C_2 | increased in CSA-Pi9 sample sets |
| LOC_Os09g21000.1 | Q6H4R6 | transport.potassium | AT1G60160 | - | C_2 | increased in CSA-Pi9 sample sets |
| LOC_Os07g47350.1 | Q8H3P9 | transport.potassium | AT3G02050 | KUP3 | C_2 | increased in CSA-Pi9 sample sets |
| LOC_Os05g35410.1 | H9BAN2 | transport.potassium | AT4G22200 | AKT2/3 | C_2 | increased in CSA-Pi9 sample sets |
| LOC_Os07g32530.1 | Q69RI8 | transport.potassium | AT5G09400 | KUP7 | C_2 | increased in CSA-Pi9 sample sets |
| LOC_Os01g70940.1 | Q942X8 | transport.potassium | AT3G02050 | KUP3 | C_2 | increased in CSA-Pi9 sample sets |
| LOC_Os02g09720.1 | Q6YUU5 | transport.ABC transporters and multidrug resistance systems | AT3G28345 | - | C_2 | increased in CSA-Pi9 sample sets |
| LOC_Os04g13220.1 | Q7XM41 | transport.ABC transporters and multidrug resistance systems | AT2G47800 | ATMRP4 | C_2 | increased in CSA-Pi9 sample sets |
| LOC_Os01g03144.1 | Q9ARU4 | transport.ABC transporters and multidrug resistance systems | AT2G01320 | - | C_2 | increased in CSA-Pi9 sample sets |
| LOC_Os01g50160.1 | Q7FMW3 | transport.ABC transporters and multidrug resistance systems | AT1G02520 | PGP11 | C_2 | increased in CSA-Pi9 sample sets |
| LOC_Os04g52900.1 | Q7XTT6 | transport.ABC transporters and multidrug resistance systems | AT2G34660 | ATMRP2 | C_2 | increased in CSA-Pi9 sample sets |
| LOC_Os03g14520.1 | Q10P28 | transport.unspecified anions | AT5G62290 | - | C_4 | increased in CSA-Pi9 sample sets |
| LOC_Os03g48940.1 | Q851D8 | transport.unspecified anions | AT5G26240 | CLC-D | C_2 | increased in CSA-Pi9 sample sets |
| LOC_Os02g57720.1 | Q9SXF8 | transport.Major Intrinsic Proteins.PIP | AT4G23400 | PIP1D | C_2 | increased in CSA-Pi9 sample sets |
| LOC_Os07g26690.1 | B7EKW3 | transport.Major Intrinsic Proteins.PIP | AT3G54820 | PIP2D | C_2 | increased in CSA-Pi9 sample sets |
| LOC_Os02g44080.1 | Q7XA61 | transport.Major Intrinsic Proteins.TIP | AT5G47450 | ATTIP2;3 | C_2 | increased in CSA-Pi9 sample sets |
| LOC_Os03g20750.1 | Q10M45 | transport.porins | AT3G20000 | TOM40 | C_2 | increased in CSA-Pi9 sample sets |
| LOC_Os01g48680.1 | Q5QM84 | transport.calcium | AT4G03560 | ATTPC1 | C_2 | increased in CSA-Pi9 sample sets |
| LOC_Os08g28170.1 | Q0J648 | transport.misc | AT2G34190 | - | C_2 | increased in CSA-Pi9 sample sets |
| LOC_Os05g27820.1 | Q5W6M0 | transport.misc | AT3G51670 | - | C_3 | increased in CSA-Pi9 sample sets |
| LOC_Os01g60230.1 | Q0JI87 | transport.misc | AT5G01990 | - | C_2 | increased in CSA-Pi9 sample sets |
| LOC_Os04g50890.1 | Q0JAI9 | transport.misc | AT1G32050 | - | C_3 | increased in CSA-Pi9 sample sets |
| LOC_Os02g39930.1 | Q6K623 | transport.misc | AT5G58070 | ATTIL | C_2 | increased in CSA-Pi9 sample sets |
| LOC_Os06g49810.1 | B9FQV2 | misc.UDP glucosyl and glucoronyl transferases | AT3G61130 | GAUT1 | C_4 | increased in CSA-Pi9 sample sets |
| LOC_Os07g37960.1 | Q84SM6 | misc.UDP glucosyl and glucoronyl transferases | AT2G35100 | ARAD1 | C_4 | increased in CSA-Pi9 sample sets |
| LOC_Os04g49960.1 | Q7XLZ9 | misc.UDP glucosyl and glucoronyl transferases | AT1G78800 | - | C_2 | increased in CSA-Pi9 sample sets |
| LOC_Os03g01760.1 | Q10SX7 | misc.UDP glucosyl and glucoronyl transferases | AT5G22940 | F8H | C_2 | increased in CSA-Pi9 sample sets |
| LOC_Os01g32364.1 | Q5QMT0 | misc.gluco-, galacto- and mannosidases | AT3G18080 | BGLU44 | C_2 | increased in CSA-Pi9 sample sets |
| LOC_Os10g17650.1 | Q339X2 | misc.gluco-, galacto- and mannosidases | AT1G26560 | BGLU40 | C_2 | increased in CSA-Pi9 sample sets |
| LOC_Os01g70520.1 | Q5JK35 | misc.gluco-, galacto- and mannosidases | AT1G02850 | BGLU11 | C_2 | increased in CSA-Pi9 sample sets |
| LOC_Os05g30350.1 | Q60DX8 | misc.gluco-, galacto- and mannosidases | AT1G02850 | BGLU11 | C_2 | increased in CSA-Pi9 sample sets |
| LOC_Os04g39900.1 | Q7XKV2 | misc.gluco-, galacto- and mannosidases | AT5G44640 | BGLU13 | C_2 | increased in CSA-Pi9 sample sets |
| LOC_Os06g21570.1 | Q5Z9Z0 | misc.gluco-, galacto- and mannosidases | AT2G44480 | BGLU17 | C_2 | increased in CSA-Pi9 sample sets |
| LOC_Os04g39880.1 | Q7XKV4 | misc.gluco-, galacto- and mannosidases | AT5G44640 | BGLU13 | C_2 | increased in CSA-Pi9 sample sets |
| LOC_Os05g01540.1 | Q65XI3 | misc.gluco-, galacto- and mannosidases | AT1G16610 | SR45 | C_4 | increased in CSA-Pi9 sample sets |
| LOC_Os06g46340.1 | Q653V4 | misc.gluco-, galacto- and mannosidases.alpha-galactosidase | AT5G11720 | - | C_2 | increased in CSA-Pi9 sample sets |
| LOC_Os04g40490.1 | Q7XUQ7 | misc.gluco-, galacto- and mannosidases.glycosyl hydrolase family 5 | AT1G13130 | - | C_2 | increased in CSA-Pi9 sample sets |
| LOC_Os07g35510.1 | Q6YVU4 | misc.beta 1,3 glucan hydrolases.glucan endo-1,3-beta-glucosidase | AT4G26830 | - | C_2 | increased in CSA-Pi9 sample sets |
| LOC_Os03g46660.1 | Q7Y179 | misc.beta 1,3 glucan hydrolases.glucan endo-1,3-beta-glucosidase | AT4G34480 | - | C_2 | increased in CSA-Pi9 sample sets |
| LOC_Os03g51240.1 | Q53K23 | misc.beta 1,3 glucan hydrolases.glucan endo-1,3-beta-glucosidase | AT4G34480 | - | C_2 | increased in CSA-Pi9 sample sets |
| LOC_Os03g14210.1 | Q10P58 | misc.beta 1,3 glucan hydrolases.glucan endo-1,3-beta-glucosidase | AT5G42100 | ATBG_PPAP | C_2 | increased in CSA-Pi9 sample sets |
| LOC_Os02g09220.1 | Q6ZIH6 | misc.cytochrome P450 | AT3G26300 | CYP71B34 | C_2 | increased in CSA-Pi9 sample sets |
| LOC_Os02g57290.1 | Q0DWE8 | misc.cytochrome P450 | AT1G31800 | CYP97A3 | C_2 | increased in CSA-Pi9 sample sets |
| LOC_Os05g33600.1 | A0A0P0WM73 | misc.cytochrome P450 | AT1G75130 | CYP721A1 | C_2 | increased in CSA-Pi9 sample sets |
| LOC_Os10g39930.1 | Q336V3 | misc.cytochrome P450 | AT3G53130 | LUT1 | C_2 | increased in CSA-Pi9 sample sets |
| LOC_Os02g09240.1 | Q6ZIH2 | misc.cytochrome P450 | AT3G26300 | CYP71B34 | C_2 | increased in CSA-Pi9 sample sets |
| LOC_Os01g08810.1 | Q9LGS7 | misc.cytochrome P450 | AT4G39490 | CYP96A10 | C_2 | increased in CSA-Pi9 sample sets |
| LOC_Os03g25480.1 | Q10KT5 | misc.cytochrome P450 | AT2G46950 | CYP709B2 | C_2 | increased in CSA-Pi9 sample sets |
| LOC_Os07g46950.2 | B9FUQ8 | misc.short chain dehydrogenase/reductase (SDR) | AT3G26760 | - | C_2 | increased in CSA-Pi9 sample sets |
| LOC_Os03g62740.1 | Q75LC6 | misc.GDSL-motif lipase | AT3G48460 | - | C_2 | increased in CSA-Pi9 sample sets |
| LOC_Os12g03470.1 | Q2QY88 | not assigned.unknown | AT3G10740 | ASD1 | C_2 | increased in CSA-Pi9 sample sets |
| LOC_Os08g40230.1 | Q6Z8N1 | not assigned.unknown | AT3G18240 | - | C_3 | increased in CSA-Pi9 sample sets |
| LOC_Os06g47310.1 | Q653F8 | not assigned.unknown | AT5G67210 | - | C_2 | increased in CSA-Pi9 sample sets |
| LOC_Os08g27840.1 | Q8H4V1 | not assigned.unknown | AT1G53310 | ATPPC1 | C_2 | increased in CSA-Pi9 sample sets |
| LOC_Os07g40930.1 | Q8H594 | not assigned.unknown | AT5G15550 | - | C_4 | increased in CSA-Pi9 sample sets |
| LOC_Os12g37720.1 | Q2QNI2 | not assigned.unknown | AT5G49930 | emb1441 | C_4 | increased in CSA-Pi9 sample sets |
| LOC_Os06g17840.1 | Q5VMT5 | not assigned.unknown | AT5G47690 | - | C_4 | increased in CSA-Pi9 sample sets |
| LOC_Os05g13950.1 | Q0DJU1 | not assigned.unknown | AT5G25757 | - | C_3 | increased in CSA-Pi9 sample sets |
| LOC_Os02g03470.1 | B9F287 | not assigned.unknown | AT3G04830 | - | C_3 | increased in CSA-Pi9 sample sets |
| LOC_Os07g43290.1 | Q7XIG9 | not assigned.unknown | - | - | C_3 | increased in CSA-Pi9 sample sets |
| LOC_Os07g19494.1 | Q6YVE2 | not assigned.unknown | AT1G24310 | - | C_4 | increased in CSA-Pi9 sample sets |
| LOC_Os07g41190.1 | Q6YW01 | not assigned.unknown | AT5G14050 | - | C_4 | increased in CSA-Pi9 sample sets |
| LOC_Os03g56390.1 | Q8H8N3 | not assigned.unknown | AT1G75200 | - | C_2 | increased in CSA-Pi9 sample sets |
| LOC_Os03g63250.1 | Q84LH7 | not assigned.unknown | AT4G24840 | - | C_3 | increased in CSA-Pi9 sample sets |
| LOC_Os01g45830.1 | Q9ASA0 | not assigned.unknown | AT1G80310 | - | C_2 | increased in CSA-Pi9 sample sets |
| LOC_Os03g15700.1 | Q10NP6 | not assigned.unknown | AT2G32600 | - | C_4 | increased in CSA-Pi9 sample sets |
| LOC_Os09g21470.1 | Q6H611 | not assigned.unknown | - | - | C_2 | increased in CSA-Pi9 sample sets |
| LOC_Os11g36060.1 | Q2R2E7 | not assigned.unknown | AT5G12410 | - | C_4 | increased in CSA-Pi9 sample sets |
| LOC_Os09g38060.1 | Q0IZS0 | not assigned.unknown | AT2G01350 | QPT | C_2 | increased in CSA-Pi9 sample sets |
| LOC_Os09g14670.1 | A0A0P0XLG1 | not assigned.unknown | AT1G53310 | ATPPC1 | C_2 | increased in CSA-Pi9 sample sets |
| LOC_Os03g05390.1 | Q10RS7 | not assigned.unknown | AT1G02260 | - | C_2 | increased in CSA-Pi9 sample sets |
| LOC_Os12g19040.1 | Q2QTN6 | not assigned.unknown | AT1G13030 | - | C_4 | increased in CSA-Pi9 sample sets |
| LOC_Os01g72049.1 | Q5JKX9 | not assigned.unknown | AT4G20010 | PTAC9 | C_4 | increased in CSA-Pi9 sample sets |
| LOC_Os07g43020.1 | Q8LHL4 | not assigned.unknown | AT1G78190 | - | C_4 | increased in CSA-Pi9 sample sets |
| LOC_Os12g12370.1 | Q2QVL8 | not assigned.unknown | AT2G43950 | OEP37 | C_2 | increased in CSA-Pi9 sample sets |
| LOC_Os05g44170.1 | Q75II2 | not assigned.unknown | AT4G18930 | - | C_2 | increased in CSA-Pi9 sample sets |
| LOC_Os10g19880.1 | Q339Q0 | not assigned.unknown | AT4G34260 | FUC95A | C_2 | increased in CSA-Pi9 sample sets |
| LOC_Os01g02050.1 | Q657S3 | not assigned.unknown | AT1G68750 | ATPPC4 | C_2 | increased in CSA-Pi9 sample sets |
| LOC_Os03g20420.1 | Q10M79 | not assigned.unknown | AT3G10740 | ASD1 | C_2 | increased in CSA-Pi9 sample sets |
| LOC_Os03g50070.1 | Q10E47 | not assigned.unknown | AT1G73650 | - | C_2 | increased in CSA-Pi9 sample sets |
| LOC_Os01g25600.1 | Q5ZBW3 | not assigned.unknown | AT1G02910 | LPA1 | C_3 | increased in CSA-Pi9 sample sets |
| LOC_Os01g07100.1 | B9ET10 | not assigned.unknown | AT5G63220 | - | C_4 | increased in CSA-Pi9 sample sets |
| LOC_Os03g02380.1 | Q8GZW6 | not assigned.unknown | AT1G64650 | - | C_2 | increased in CSA-Pi9 sample sets |
| LOC_Os03g61560.1 | Q850Z4 | not assigned.unknown | AT3G16810 | APUM24 | C_4 | increased in CSA-Pi9 sample sets |
| LOC_Os02g44690.1 | Q6EU87 | not assigned.unknown | AT2G23940 | - | C_4 | increased in CSA-Pi9 sample sets |
| LOC_Os02g23890.1 | Q67U13 | not assigned.unknown | AT3G20920 | - | C_4 | increased in CSA-Pi9 sample sets |
| LOC_Os01g49760.1 | Q0JK71 | not assigned.unknown | AT1G15060 | - | C_2 | increased in CSA-Pi9 sample sets |
| LOC_Os10g37520.1 | Q9FWD6 | not assigned.unknown | AT1G64650 | - | C_2 | increased in CSA-Pi9 sample sets |
| LOC_Os02g05370.1 | Q0E3Y8 | not assigned.unknown | AT5G24630 | BIN4 | C_4 | increased in CSA-Pi9 sample sets |
| LOC_Os04g42320.1 | Q0JBZ3 | not assigned.unknown | AT4G31880 | - | C_4 | increased in CSA-Pi9 sample sets |
| LOC_Os01g03750.1 | Q5ZEG0 | not assigned.unknown | AT1G67080 | ABA4 | C_2 | increased in CSA-Pi9 sample sets |
| LOC_Os03g08480.1 | Q0DUJ6 | not assigned.unknown | AT1G06190 | - | C_4 | increased in CSA-Pi9 sample sets |
| LOC_Os06g41020.1 | Q69WY3 | not assigned.unknown | AT5G12960 | - | C_2 | increased in CSA-Pi9 sample sets |
| LOC_Os11g03730.1 | Q2RAZ7 | not assigned.unknown | AT3G10740 | ASD1 | C_2 | increased in CSA-Pi9 sample sets |
| LOC_Os11g03780.1 | Q2RAZ2 | not assigned.unknown | AT3G10740 | ASD1 | C_2 | increased in CSA-Pi9 sample sets |
| LOC_Os06g06370.1 | B9FRL5 | not assigned.unknown | AT1G08030 | - | C_4 | increased in CSA-Pi9 sample sets |
| LOC_Os05g02820.1 | B9FGI8 | not assigned.unknown | AT1G65010 | - | C_4 | increased in CSA-Pi9 sample sets |
| LOC_Os07g34730.1 | Q8GVN6 | not assigned.unknown | AT5G60620 | - | C_3 | increased in CSA-Pi9 sample sets |
| LOC_Os01g62650.1 | Q0JHT2 | not assigned.unknown | AT2G29190 | APUM2 | C_4 | increased in CSA-Pi9 sample sets |
| LOC_Os07g37250.1 | Q84PB7 | not assigned.unknown | AT2G20890 | PSB29 | C_2 | increased in CSA-Pi9 sample sets |
| LOC_Os02g10190.1 | Q6H7P9 | not assigned.unknown | AT5G12960 | - | C_2 | increased in CSA-Pi9 sample sets |
| LOC_Os03g06720.1 | Q10RD6 | not assigned.unknown | AT5G43745 | - | C_2 | increased in CSA-Pi9 sample sets |
| LOC_Os02g51470.1 | Q6Z8K7 | PS.lightreaction.ATP synthase.delta chain | AT4G09650 | ATPD | C_5 | increased in DJ sample sets |
| LOC_Os08g39300.1 | Q6ZFI6 | PS.photorespiration.aminotransferases peroxisomal | AT2G13360 | AGT | C_6 | increased in DJ sample sets |
| LOC_Os06g40640.2 | Q69V57 | PS.calvin cycle.aldolase | AT4G26530 | - | C_6 | increased in DJ sample sets |
| LOC_Os02g47020.1 | Q6Z8F4 | PS.calvin cycle.PRK | AT1G32060 | PRK | C_6 | increased in DJ sample sets |
| LOC_Os01g69030.1 | Q0JGK4 | major CHO metabolism.synthesis.sucrose.SPS | AT1G04920 | ATSPS3F | C_6 | increased in DJ sample sets |
| LOC_Os06g51084.1 | Q0D9D0 | major CHO metabolism.synthesis.starch.starch branching | AT5G03650 | SBE2.2 | C_6 | increased in DJ sample sets |
| LOC_Os01g66940.1 | Q0JGZ6 | major CHO metabolism.degradation.sucrose.fructokinase | AT1G06020 | - | C_6 | increased in DJ sample sets |
| LOC_Os04g33740.1 | B5KNJ4 | major CHO metabolism.degradation.sucrose.invertases.cell wall | AT2G36190 | AtcwINV4 | C_6 | increased in DJ sample sets |
| LOC_Os03g09250.1 | O64437 | minor CHO metabolism.myo-inositol.InsP Synthases | AT2G22240 | ATMIPS2 | C_6 | increased in DJ sample sets |
| LOC_Os05g07130.1 | Q0DKG9 | glycolysis.cytosolic branch.fructose-2,6-bisphosphatase (Fru2,6BisPase) | AT1G07110 | F2KP | C_6 | increased in DJ sample sets |
| LOC_Os08g33440.1 | Q6ZKB1 | TCA / org. transformation.TCA.pyruvate DH.E2 | AT3G25860 | LTA2 | C_5 | increased in DJ sample sets |
| LOC_Os01g61410.1 | B9EU97 | mitochondrial electron transport / ATP synthesis.NADH-DH.type II.internal matrix | AT1G07180 | ATNDI1 | C_6 | increased in DJ sample sets |
| LOC_Os04g52370.1 | B7EA04 | cell wall.precursor synthesis | AT1G31070 | - | C_6 | increased in DJ sample sets |
| LOC_Os05g29990.1 | Q6I683 | cell wall.precursor synthesis.UXS | AT3G62830 | UXS2 | C_6 | increased in DJ sample sets |
| LOC_Os08g03670.1 | B9FYX6 | cell wall.hemicellulose synthesis | AT4G21060 | - | C_5 | increased in DJ sample sets |
| LOC_Os12g36810.1 | Q2QNT4 | cell wall.degradation.pectate lyases and polygalacturonases | AT3G16850 | - | C_6 | increased in DJ sample sets |
| LOC_Os02g02700.1 | Q6YUT4 | lipid metabolism.FA synthesis and FA elongation.acyl coa ligase | AT1G20560 | AAE1 | C_6 | increased in DJ sample sets |
| LOC_Os01g16960.1 | Q5NBQ0 | lipid metabolism.FA synthesis and FA elongation.pyruvate kinase | AT5G56350 | - | C_6 | increased in DJ sample sets |
| LOC_Os01g08760.1 | Q0JQ41 | lipid metabolism.Phospholipid synthesis.choline kinase | AT1G71697 | ATCK1 | C_6 | increased in DJ sample sets |
| LOC_Os10g31820.1 | Q0IX96 | N-metabolism.ammonia metabolism.glutamine synthase | AT3G53180 | - | C_6 | increased in DJ sample sets |
| LOC_Os04g56400.1 | P14655 | N-metabolism.ammonia metabolism.glutamine synthase | AT5G35630 | GS2 | C_6 | increased in DJ sample sets |
| LOC_Os08g39300.1 | Q6ZFI6 | amino acid metabolism.synthesis.central amino acid metabolism.alanine.alanine-glyoxylate aminotransferase | AT2G13360 | AGT | C_6 | increased in DJ sample sets |
| LOC_Os03g42110.1 | Q6AV34 | amino acid metabolism.synthesis.aspartate family.misc.homoserine.aspartate semialdehyde dehydrogenase | AT2G19940 | - | C_6 | increased in DJ sample sets |
| LOC_Os05g48450.1 | Q6AT43 | amino acid metabolism.synthesis.branched chain group.common.branched-chain amino acid aminotransferase | AT5G65780 | ATBCAT-5 | C_6 | increased in DJ sample sets |
| LOC_Os06g44460.1 | A3BE72 | amino acid metabolism.synthesis.serine-glycine-cysteine group.serine.phosphoglycerate dehydrogenase | AT1G17745 | PGDH | C_6 | increased in DJ sample sets |
| LOC_Os08g39300.1 | Q6ZFI6 | amino acid metabolism.synthesis.serine-glycine-cysteine group.glycine.serine glyoxylate aminotransferase | AT2G13360 | AGT | C_6 | increased in DJ sample sets |
| LOC_Os06g42560.1 | Q67VM1 | amino acid metabolism.synthesis.aromatic aa.tryptophan.tryptophan synthase | AT5G38530 | - | C_6 | increased in DJ sample sets |
| LOC_Os10g37340.1 | Q7XCS3 | amino acid metabolism.degradation.aspartate family.methionine.methionine gamma-lyase | AT1G64660 | ATMGL | C_6 | increased in DJ sample sets |
| LOC_Os02g54254.1 | E3WF37 | amino acid metabolism.degradation.aspartate family.lysine | AT4G33150 | - | C_6 | increased in DJ sample sets |
| LOC_Os02g07160.1 | Q6H4V1 | secondary metabolism.isoprenoids.tocopherol biosynthesis.hydroxyphenylpyruvate dioxygenase | AT1G06570 | PDS1 | C_6 | increased in DJ sample sets |
| LOC_Os12g33610.1 | A0A0P0YAL3 | secondary metabolism.phenylpropanoids.lignin biosynthesis.PAL | AT2G37040 | pal1 | C_6 | increased in DJ sample sets |
| LOC_Os03g16610.1 | Q10ND7 | secondary metabolism.simple phenols | AT5G60020 | LAC17 | C_6 | increased in DJ sample sets |
| LOC_Os09g07460.1 | Q6ZXK6 | hormone metabolism.jasmonate.induced-regulated-responsive-activated | AT3G07720 | - | C_6 | increased in DJ sample sets |
| LOC_Os03g04060.1 | Q10S66 | stress.biotic | AT3G12500 | ATHCHIB | C_6 | increased in DJ sample sets |
| LOC_Os09g32080.1 | Q0J0L1 | stress.biotic | AT1G05850 | POM1 | C_6 | increased in DJ sample sets |
| LOC_Os08g40690.1 | O49825 | stress.biotic | AT5G24090 | - | C_6 | increased in DJ sample sets |
| LOC_Os02g39330.1 | Q6K8R2 | stress.biotic | AT3G54420 | ATEP3 | C_6 | increased in DJ sample sets |
| LOC_Os10g18820.1 | Q8LMF1 | stress.biotic | - | - | C_6 | increased in DJ sample sets |
| LOC_Os07g44250.1 | Q7XIX5 | stress.biotic.PR-proteins | AT1G64160 | - | C_6 | increased in DJ sample sets |
| LOC_Os01g13760.1 | Q9FTT4 | stress.abiotic.heat | AT2G20560 | - | C_5 | increased in DJ sample sets |
| LOC_Os01g08860.1 | E5D3J5 | stress.abiotic.heat | AT5G12020 | HSP17.6II | C_5 | increased in DJ sample sets |
| LOC_Os05g48810.1 | Q688X8 | stress.abiotic.heat | AT2G20560 | - | C_5 | increased in DJ sample sets |
| LOC_Os09g31486.1 | B9G4B3 | stress.abiotic.heat | AT5G09590 | MTHSC70-2 | C_5 | increased in DJ sample sets |
| LOC_Os01g23740.1 | Q942L2 | redox.thioredoxin | AT2G47470 | ATPDIL2-1 | C_5 | increased in DJ sample sets |
| LOC_Os03g17860.1 | Q10N04 | redox.thioredoxin | AT1G07960 | ATPDIL5-1 | C_5 | increased in DJ sample sets |
| LOC_Os05g01440.1 | B9FM04 | nucleotide metabolism.synthesis.purine.FGAR amidotransferase | AT1G74260 | PUR4 | C_6 | increased in DJ sample sets |
| LOC_Os03g49220.1 | Q851S8 | nucleotide metabolism.synthesis.purine.adenylosuccinate synthase | AT3G57610 | ADSS | C_5 | increased in DJ sample sets |
| LOC_Os02g48390.1 | Q6ZFT5 | nucleotide metabolism.synthesis.PRS-PP | AT2G42910 | - | C_6 | increased in DJ sample sets |
| LOC_Os05g08950.1 | A0A0P0WIZ3 | nucleotide metabolism.salvage.phosphoribosyltransferases.hgprt | AT1G71750 | - | C_5 | increased in DJ sample sets |
| LOC_Os07g43170.1 | Q7XI40 | nucleotide metabolism.phosphotransfer and pyrophosphatases.uridylate kinase | AT5G26667 | PYR6 | C_5 | increased in DJ sample sets |
| LOC_Os12g36194.1 | Q0IMS5 | nucleotide metabolism.phosphotransfer and pyrophosphatases.nucleoside diphosphate kinase | AT5G63310 | NDPK2 | C_5 | increased in DJ sample sets |
| LOC_Os10g41410.2 | Q7XC37 | nucleotide metabolism.phosphotransfer and pyrophosphatases.nucleoside diphosphate kinase | AT4G09320 | NDPK1 | C_5 | increased in DJ sample sets |
| LOC_Os03g46640.1 | Q10FF9 | nucleotide metabolism.deoxynucleotide metabolism.dUTP diphosphatase | AT3G46940 | - | C_5 | increased in DJ sample sets |
| LOC_Os09g39270.1 | Q0IZJ8 | RNA.processing | AT4G04885 | PCFS4 | C_5 | increased in DJ sample sets |
| LOC_Os07g12320.1 | Q8LH68 | RNA.processing | AT2G47990 | SWA1 | C_5 | increased in DJ sample sets |
| LOC_Os02g13990.1 | Q6EUK2 | RNA.processing | AT1G09760 | U2A' | C_5 | increased in DJ sample sets |
| LOC_Os11g03570.1 | Q2RB13 | RNA.processing | AT5G65180 | - | C_5 | increased in DJ sample sets |
| LOC_Os05g04940.1 | Q75KG9 | RNA.processing | AT4G15520 | - | C_5 | increased in DJ sample sets |
| LOC_Os02g30624.1 | Q6K2E9 | RNA.processing | AT2G43810 | - | C_5 | increased in DJ sample sets |
| LOC_Os11g45590.1 | B9G8T5 | RNA.processing.splicing | AT4G36690 | ATU2AF65A | C_5 | increased in DJ sample sets |
| LOC_Os02g39890.1 | Q84L14 | RNA.processing.splicing | AT5G44200 | CBP20 | C_5 | increased in DJ sample sets |
| LOC_Os03g06440.1 | Q10RG1 | RNA.processing.RNA helicase | AT1G05460 | SDE3 | C_5 | increased in DJ sample sets |
| LOC_Os01g07740.1 | B7EQA6 | RNA.processing.RNA helicase | AT5G14610 | - | C_5 | increased in DJ sample sets |
| LOC_Os07g42395.1 | Q8GSD5 | RNA.transcription | AT3G16980 | NRPB9A | C_5 | increased in DJ sample sets |
| LOC_Os08g25460.1 | Q6ZB67 | RNA.transcription | AT1G03280 | - | C_5 | increased in DJ sample sets |
| LOC_Os10g10990.1 | Q8LMN8 | RNA.transcription | AT4G12610 | RAP74 | C_5 | increased in DJ sample sets |
| LOC_Os08g06070.1 | Q6ZD92 | RNA.regulation of transcription | AT1G79730 | ELF7 | C_5 | increased in DJ sample sets |
| LOC_Os08g06240.1 | Q84PV0 | RNA.regulation of transcription.MYB domain transcription factor family | AT5G45420 | - | C_5 | increased in DJ sample sets |
| LOC_Os07g28430.1 | Q6AWX8 | RNA.regulation of transcription.General Transcription | AT3G13960 | AtGRF5 | C_5 | increased in DJ sample sets |
| LOC_Os01g34330.1 | Q5QLH2 | RNA.regulation of transcription.putative transcription regulator | AT4G11560 | - | C_5 | increased in DJ sample sets |
| LOC_Os03g44900.1 | Q7XZZ2 | RNA.regulation of transcription.putative transcription regulator | AT5G18230 | - | C_5 | increased in DJ sample sets |
| LOC_Os09g38970.1 | A0A0P0XQE0 | RNA.regulation of transcription.unclassified | AT4G15080 | - | C_5 | increased in DJ sample sets |
| LOC_Os12g23180.1 | Q2QSR7 | RNA.regulation of transcription.unclassified | AT1G09340 | CRB | C_6 | increased in DJ sample sets |
| LOC_Os02g39700.1 | Q6K6X2 | RNA.regulation of transcription.unclassified | AT4G32720 | AtLa1 | C_5 | increased in DJ sample sets |
| LOC_Os02g07070.1 | B9F385 | RNA.RNA binding | AT3G54230 | - | C_5 | increased in DJ sample sets |
| LOC_Os12g12360.1 | Q2QVL9 | RNA.RNA binding | AT2G43970 | - | C_5 | increased in DJ sample sets |
| LOC_Os04g39629.1 | Q7XR25 | RNA.RNA binding | AT3G12640 | - | C_5 | increased in DJ sample sets |
| LOC_Os08g39440.1 | Q6ZK85 | RNA.RNA binding | AT3G12640 | - | C_5 | increased in DJ sample sets |
| LOC_Os05g04850.1 | B7FAL5 | RNA.RNA binding | AT1G51510 | Y14 | C_5 | increased in DJ sample sets |
| LOC_Os04g02000.1 | B9FD24 | RNA.RNA binding | AT2G17975 | - | C_5 | increased in DJ sample sets |
| LOC_Os11g41890.1 | Q2R0P4 | RNA.RNA binding | AT3G07810 | - | C_5 | increased in DJ sample sets |
| LOC_Os02g52250.1 | Q6K8D9 | DNA.synthesis/chromatin structure | AT1G77180 | - | C_5 | increased in DJ sample sets |
| LOC_Os03g43800.1 | Q10G93 | DNA.unspecified | AT3G21430 | ATALY3 | C_5 | increased in DJ sample sets |
| LOC_Os01g21180.1 | Q8RZU5 | protein.aa activation | AT1G09150 | - | C_5 | increased in DJ sample sets |
| LOC_Os02g04700.1 | Q6Z2Y0 | protein.aa activation.aspartate-tRNA ligase | AT4G31180 | - | C_6 | increased in DJ sample sets |
| LOC_Os03g62630.1 | Q75LD8 | protein.synthesis.ribosomal protein.prokaryotic.chloroplast.30S subunit.S6 | AT1G64510 | - | C_5 | increased in DJ sample sets |
| LOC_Os07g46440.1 | Q7XAM9 | protein.synthesis.ribosomal protein.prokaryotic.chloroplast.30S subunit.S9 | AT3G49080 | - | C_5 | increased in DJ sample sets |
| LOC_Os02g04460.1 | Q0E446 | protein.synthesis.ribosomal protein.prokaryotic.chloroplast.50S subunit.L3 | AT2G43030 | - | C_5 | increased in DJ sample sets |
| LOC_Os03g15870.1 | Q10NM5 | protein.synthesis.ribosomal protein.prokaryotic.chloroplast.50S subunit.L4 | AT1G07320 | RPL4 | C_5 | increased in DJ sample sets |
| LOC_Os03g12020.1 | Q10PV6 | protein.synthesis.ribosomal protein.prokaryotic.chloroplast.50S subunit.L15 | AT3G25920 | RPL15 | C_5 | increased in DJ sample sets |
| LOC_Os05g48410.1 | Q6I600 | protein.synthesis.ribosomal protein.prokaryotic.chloroplast.50S subunit.L21 | AT4G30930 | NFD1 | C_5 | increased in DJ sample sets |
| LOC_Os02g15900.1 | Q6K241 | protein.synthesis.ribosomal protein.prokaryotic.chloroplast.50S subunit.L21 | AT1G35680 | - | C_5 | increased in DJ sample sets |
| LOC_Os03g20100.1 | Q10MB2 | protein.synthesis.ribosomal protein.prokaryotic.unknown organellar.30S subunit.S1 | AT5G30510 | RPS1 | C_5 | increased in DJ sample sets |
| LOC_Os08g21840.1 | Q6UUH5 | protein.synthesis.ribosomal protein.prokaryotic.unknown organellar.50S subunit.L15 | AT5G64670 | - | C_5 | increased in DJ sample sets |
| LOC_Os01g01060.1 | Q93VC6 | protein.synthesis.ribosomal protein.eukaryotic.40S subunit.S5 | AT2G37270 | ATRPS5B | C_5 | increased in DJ sample sets |
| LOC_Os02g28810.1 | P49199 | protein.synthesis.ribosomal protein.eukaryotic.40S subunit.S8 | AT5G59240 | - | C_5 | increased in DJ sample sets |
| LOC_Os08g02410.1 | Q69UI1 | protein.synthesis.ribosomal protein.eukaryotic.40S subunit.S13 | AT4G00100 | ATRPS13A | C_5 | increased in DJ sample sets |
| LOC_Os03g58430.1 | Q851P5 | protein.synthesis.ribosomal protein.eukaryotic.40S subunit.S15 | AT5G09500 | - | C_5 | increased in DJ sample sets |
| LOC_Os10g27190.1 | Q7XEQ3 | protein.synthesis.ribosomal protein.eukaryotic.40S subunit.S17 | AT3G10610 | - | C_5 | increased in DJ sample sets |
| LOC_Os03g31090.1 | P40978 | protein.synthesis.ribosomal protein.eukaryotic.40S subunit.S19 | AT5G61170 | - | C_5 | increased in DJ sample sets |
| LOC_Os12g38000.1 | Q2QNF3 | protein.synthesis.ribosomal protein.eukaryotic.60S subunit.L8 | AT4G36130 | - | C_5 | increased in DJ sample sets |
| LOC_Os03g22180.1 | Q10LN7 | protein.synthesis.ribosomal protein.eukaryotic.60S subunit.L18 | AT5G27850 | - | C_5 | increased in DJ sample sets |
| LOC_Os03g21940.1 | Q10LR6 | protein.synthesis.ribosomal protein.eukaryotic.60S subunit.L19 | AT3G16780 | - | C_5 | increased in DJ sample sets |
| LOC_Os07g47710.1 | Q6YSX0 | protein.synthesis.ribosomal protein.eukaryotic.60S subunit.L22 | AT3G05560 | - | C_5 | increased in DJ sample sets |
| LOC_Os05g40820.1 | Q75KZ3 | protein.synthesis.ribosomal protein.eukaryotic.60S subunit.L24 | AT2G36620 | RPL24A | C_5 | increased in DJ sample sets |
| LOC_Os05g46430.1 | Q5TKP3 | protein.synthesis.ribosomal protein.eukaryotic.60S subunit.L28 | AT4G29410 | - | C_5 | increased in DJ sample sets |
| LOC_Os05g37330.1 | Q65WT5 | protein.synthesis.ribosomal protein.eukaryotic.60S subunit.P2 | - | RLA2B | C_5 | increased in DJ sample sets |
| LOC_Os01g13080.1 | Q5NB69 | protein.synthesis.ribosomal protein.eukaryotic.60S subunit.P3 | AT4G25890 | - | C_5 | increased in DJ sample sets |
| LOC_Os01g09510.1 | Q5SNH7 | protein.synthesis.ribosomal protein.eukaryotic.60S subunit.unknown | - | RLA2A | C_5 | increased in DJ sample sets |
| LOC_Os07g08330.1 | Q6ZLB8 | protein.synthesis.ribosomal protein.eukaryotic.60S subunit.L4/L1 | AT3G09630 | - | C_5 | increased in DJ sample sets |
| LOC_Os02g54470.1 | Q6K8U8 | protein.synthesis.ribosomal protein.eukaryotic.60S subunit.L35A | AT1G74270 | - | C_5 | increased in DJ sample sets |
| LOC_Os02g37870.1 | Q69L77 | protein.synthesis.misc ribosomal protein.BRIX | AT3G15460 | - | C_5 | increased in DJ sample sets |
| LOC_Os07g36940.1 | B9FXV5 | protein.synthesis.initiation | AT3G60240 | EIF4G | C_5 | increased in DJ sample sets |
| LOC_Os07g34589.2 | B7F683 | protein.synthesis.initiation | AT1G54290 | - | C_5 | increased in DJ sample sets |
| LOC_Os01g09890.1 | Q5SMX7 | protein.synthesis.initiation | AT5G11900 | - | C_5 | increased in DJ sample sets |
| LOC_Os07g46750.1 | P29545 | protein.synthesis.elongation | AT5G19510 | - | C_5 | increased in DJ sample sets |
| LOC_Os11g43600.1 | Q2R068 | protein.synthesis.release | AT3G62910 | APG3 | C_5 | increased in DJ sample sets |
| LOC_Os07g29320.1 | Q84RU7 | protein.synthesis.ribosomal RNA | AT3G22660 | - | C_5 | increased in DJ sample sets |
| LOC_Os12g15420.1 | Q2QUQ9 | protein.synthesis.misc | AT1G80930 | - | C_5 | increased in DJ sample sets |
| LOC_Os04g36859.1 | Q7XUX4 | protein.targeting.secretory pathway.ER | AT2G39960 | - | C_5 | increased in DJ sample sets |
| LOC_Os01g71080.1 | Q8S1V1 | protein.degradation.aspartate protease | AT1G03230 | - | C_6 | increased in DJ sample sets |
| LOC_Os10g01134.1 | Q10A76 | protein.degradation.serine protease | AT1G73270 | scpl6 | C_6 | increased in DJ sample sets |
| LOC_Os04g09720.1 | Q7XSG1 | protein.degradation.serine protease | AT2G23000 | scpl10 | C_6 | increased in DJ sample sets |
| LOC_Os02g46260.1 | B9F1V9 | protein.degradation.serine protease | AT5G09640 | SNG2 | C_6 | increased in DJ sample sets |
| LOC_Os12g17910.1 | Q2QU06 | protein.folding | AT2G28000 | CPN60A | C_5 | increased in DJ sample sets |
| LOC_Os09g11250.1 | Q6EPR4 | protein.folding | AT5G55200 | - | C_5 | increased in DJ sample sets |
| LOC_Os01g66980.1 | Q5JLV2 | protein.folding | AT2G30695 | - | C_5 | increased in DJ sample sets |
| LOC_Os08g25090.1 | Q6Z0G0 | protein.folding | AT4G26780 | AR192 | C_5 | increased in DJ sample sets |
| LOC_Os02g14929.1 | Q6K3R8 | protein.folding | AT5G26360 | - | C_5 | increased in DJ sample sets |
| LOC_Os02g54060.1 | Q6K826 | protein.folding | AT5G20720 | CPN20 | C_5 | increased in DJ sample sets |
| LOC_Os02g02620.1 | Q6ZH45 | protein.glycosylation | AT3G48820 | - | C_5 | increased in DJ sample sets |
| LOC_Os03g11980.1 | Q10PW1 | protein.glycosylation.alpha-1,3-mannosyl-glycoprotein-beta-1,2-N-acetylglucosaminyltransferase(GnTI) | AT1G05590 | HEXO2 | C_6 | increased in DJ sample sets |
| LOC_Os03g11980.1 | Q10PW1 | protein.glycosylation.alpha-1,3-mannosyl-glycoprotein-beta-1,2-N-acetylglucosaminyltransferase(GnTI) | AT1G05590 | HEXO2 | C_6 | increased in DJ sample sets |
| LOC_Os01g22550.1 | Q5ZDR9 | protein.glycosylation.beta-1,3-galactosyltransferase(beta-1,3-GalT) | AT3G06440 | - | C_5 | increased in DJ sample sets |
| LOC_Os12g07290.1 | Q2QX39 | protein.glycosylation.alpha-1,4-fucosyltransferase(alpha-1,4-FucT) | AT1G71990 | FUT13 | C_6 | increased in DJ sample sets |
| LOC_Os01g61310.1 | Q941W1 | signaling.light | AT1G07090 | LSH6 | C_5 | increased in DJ sample sets |
| LOC_Os04g30890.1 | B9FES7 | cell.division | AT3G11450 | - | C_5 | increased in DJ sample sets |
| LOC_Os02g52360.1 | Q6Z6H8 | cell.cycle.peptidylprolyl isomerase | AT2G15790 | SQN | C_5 | increased in DJ sample sets |
| LOC_Os03g49210.1 | Q851S7 | development.unspecified | AT5G14520 | - | C_5 | increased in DJ sample sets |
| LOC_Os07g12320.1 | Q8LH68 | development.unspecified | AT2G47990 | SWA1 | C_5 | increased in DJ sample sets |
| LOC_Os03g60340.1 | Q10BH4 | development.unspecified | AT2G40320 | - | C_6 | increased in DJ sample sets |
| LOC_Os03g18140.1 | Q10MX2 | development.unspecified | AT3G55990 | ESK1 | C_6 | increased in DJ sample sets |
| LOC_Os03g21250.1 | Q10LZ0 | misc.UDP glucosyl and glucoronyl transferases | AT2G38650 | GAUT7 | C_6 | increased in DJ sample sets |
| LOC_Os11g03160.1 | Q2RB54 | misc.UDP glucosyl and glucoronyl transferases | AT2G38650 | GAUT7 | C_5 | increased in DJ sample sets |
| LOC_Os08g03670.1 | B9FYX6 | misc.UDP glucosyl and glucoronyl transferases | AT4G21060 | - | C_5 | increased in DJ sample sets |
| LOC_Os01g70190.1 | Q8S1X8 | misc.UDP glucosyl and glucoronyl transferases | AT5G61840 | GUT1 | C_5 | increased in DJ sample sets |
| LOC_Os03g15020.1 | B7EDJ0 | misc.gluco-, galacto- and mannosidases.beta-galactosidase | AT2G28470 | BGAL8 | C_6 | increased in DJ sample sets |
| LOC_Os04g40510.1 | A0A0P0WBG8 | misc.gluco-, galacto- and mannosidases.glycosyl hydrolase family 5 | AT1G13130 | - | C_6 | increased in DJ sample sets |
| LOC_Os07g35520.1 | A3BKR0 | misc.beta 1,3 glucan hydrolases.glucan endo-1,3-beta-glucosidase | AT4G26830 | - | C_6 | increased in DJ sample sets |
| LOC_Os07g35480.1 | Q6YVU7 | misc.beta 1,3 glucan hydrolases.glucan endo-1,3-beta-glucosidase | AT5G55180 | - | C_6 | increased in DJ sample sets |
| LOC_Os01g71810.1 | Q0JG30 | misc.beta 1,3 glucan hydrolases.glucan endo-1,3-beta-glucosidase | AT4G16260 | - | C_6 | increased in DJ sample sets |
| LOC_Os02g58070.1 | Q6K7Q8 | misc.oxidases - copper, flavone etc. | AT1G20270 | - | C_6 | increased in DJ sample sets |
| LOC_Os08g02230.1 | Q6YXT5 | misc.oxidases - copper, flavone etc. | AT2G46740 | - | C_6 | increased in DJ sample sets |
| LOC_Os06g39780.1 | Q69X58 | misc.cytochrome P450 | AT2G45550 | CYP76C4 | C_6 | increased in DJ sample sets |
| LOC_Os03g13560.1 | Q10PC8 | misc.oxygenases | AT1G14710 | - | C_5 | increased in DJ sample sets |
| LOC_Os09g07460.1 | Q6ZXK6 | misc.myrosinases-lectin-jacalin | AT3G07720 | - | C_6 | increased in DJ sample sets |
| LOC_Os09g31310.1 | Q5U9F2 | misc.GCN5-related N-acetyltransferase | AT5G15770 | AtGNA1 | C_6 | increased in DJ sample sets |
| LOC_Os02g17330.1 | Q0E243 | misc.aminotransferases.aminotransferase class IV family protein | AT5G57850 | - | C_6 | increased in DJ sample sets |
| LOC_Os11g03390.1 | Q2RB31 | not assigned.unknown | AT2G21530 | - | C_5 | increased in DJ sample sets |
| LOC_Os03g62110.2 | Q851M0 | not assigned.unknown | AT4G36940 | NAPRT1 | C_6 | increased in DJ sample sets |
| LOC_Os11g13870.1 | Q53N85 | not assigned.unknown | AT4G09990 | - | C_6 | increased in DJ sample sets |
| LOC_Os12g18880.1 | P49030 | not assigned.unknown | AT1G02140 | MEE63 | C_5 | increased in DJ sample sets |
| LOC_Os06g02210.1 | Q5VRZ8 | not assigned.unknown | AT4G24340 | - | C_6 | increased in DJ sample sets |
| LOC_Os01g41190.1 | B7FAK7 | not assigned.unknown | AT1G80000 | - | C_5 | increased in DJ sample sets |
| LOC_Os10g22520.1 | Q8RU06 | not assigned.unknown | - | - | C_6 | increased in DJ sample sets |
| LOC_Os02g02560.1 | Q6ZGL5 | not assigned.unknown | AT5G17310 | - | C_6 | increased in DJ sample sets |
| LOC_Os01g08960.1 | Q9LGR1 | not assigned.unknown | AT5G14240 | - | C_5 | increased in DJ sample sets |
| LOC_Os10g28080.1 | Q9FYR9 | not assigned.unknown | - | - | C_6 | increased in DJ sample sets |
| LOC_Os02g06380.1 | A0A0P0VFA5 | not assigned.unknown | AT5G67210 | - | C_6 | increased in DJ sample sets |
| LOC_Os08g01840.1 | Q6ZC63 | not assigned.unknown | AT2G02570 | - | C_5 | increased in DJ sample sets |
| LOC_Os05g01910.1 | B7F8F5 | not assigned.unknown | AT2G29190 | APUM2 | C_5 | increased in DJ sample sets |
| LOC_Os02g02360.1 | Q6Z7B7 | not assigned.unknown | AT5G41190 | - | C_5 | increased in DJ sample sets |
| LOC_Os01g56300.1 | Q5ZAQ3 | not assigned.unknown | AT3G22270 | - | C_5 | increased in DJ sample sets |
| LOC_Os09g34190.1 | Q69MT1 | not assigned.unknown | AT5G48370 | - | C_6 | increased in DJ sample sets |
| LOC_Os03g02450.1 | Q10SP1 | not assigned.unknown | AT2G46580 | - | C_6 | increased in DJ sample sets |
| LOC_Os11g13810.1 | Q2R859 | not assigned.unknown | AT5G49900 | - | C_6 | increased in DJ sample sets |
| LOC_Os03g08810.1 | Q10QR1 | not assigned.unknown | AT5G62440 | - | C_5 | increased in DJ sample sets |
| LOC_Os06g21560.1 | Q5Z9Z1 | not assigned.unknown | AT5G06830 | - | C_5 | increased in DJ sample sets |
| LOC_Os10g34380.1 | Q337J6 | not assigned.unknown | AT3G19840 | - | C_5 | increased in DJ sample sets |
| LOC_Os03g63670.1 | Q84T85 | not assigned.unknown | AT5G08550 | ILP1 | C_5 | increased in DJ sample sets |
| LOC_Os04g01690.1 | Q7XRA1 | not assigned.unknown | AT2G16500 | ADC1 | C_6 | increased in DJ sample sets |
| LOC_Os04g56760.1 | Q7XM15 | not assigned.unknown | AT2G30000 | - | C_5 | increased in DJ sample sets |
| LOC_Os12g42150.1 | Q2QM63 | not assigned.unknown | AT1G65030 | - | C_5 | increased in DJ sample sets |
